# Confinement Effect in Metal–Organic Framework Cu_3_(**BTC**)_2_ for Enhancing Shape Selectivity of Radical Difunctionalization of Alkenes

**DOI:** 10.1021/acsomega.3c09911

**Published:** 2024-03-11

**Authors:** Mochen Li, Zhi Feng, Chunying Duan, Tiexin Zhang, Yusheng Shi

**Affiliations:** † State Key Laboratory of Fine Chemicals, School of Chemical Engineering, School of Chemistry, Dalian University of Technology, Dalian 116024, P. R. China; ‡ State Key Laboratory of Coordination Chemistry, Nanjing University, Nanjing 210023, P. R. China; § Jiangsu Yangnong Chemical Group Co., Ltd., Yangzhou 225001, P. R. China

## Abstract

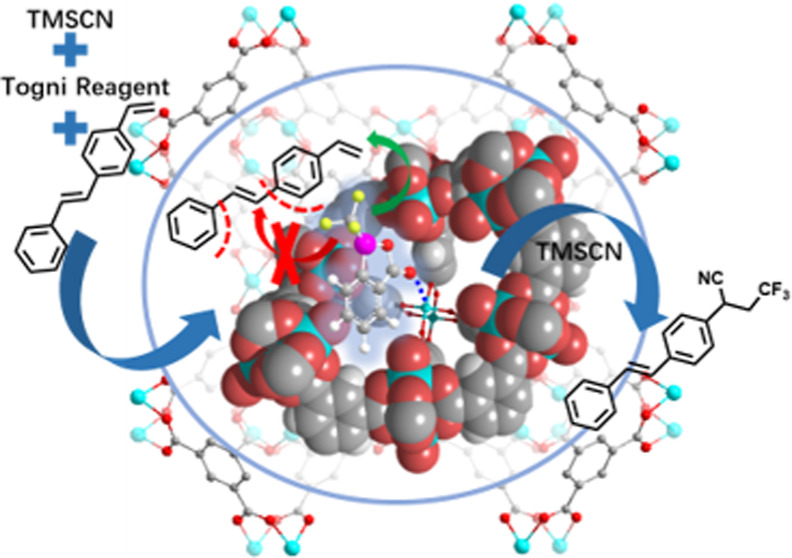

The radical difunctionalization of alkenes plays a vital role in pharmacy, but the conventional homogeneous catalytic systems are challenging in selectivity and sustainability to afford the target molecules. Herein, the famous readily available metal–organic framework (MOF), Cu_3_(**BTC**)_2_, has been applied to cyano-trifluoromethylation of alkenes as a high-performance and recyclable heterogeneous catalyst, which possesses copper(II) active sites residing in funnel-like cavities. Under mild conditions, styrene derivatives and various unactivated olefins could be smoothly transformed into the corresponding cyano-trifluoromethylation products. Moreover, the transformation brought about by the active copper center in confined environments achieved regio- and shape selectivity. To understand the enhanced selectivity, the activation manner of the MOF catalyst was studied with control catalytic experiments such as FT-IR and UV–vis absorption spectroscopy of substrate-incorporated Cu_3_(**BTC**)_2_, which elucidated that the catalyst underwent a radical transformation with the intermediates confined in the MOF cavity, and the confinement effect endowed the method with pronounced selectivities.

## 1 Introduction

Compounds bearing trifluoromethyl (CF_3_) have been used as pharmaceuticals, agrochemicals, and functional materials.^1^ Introducing trifluoromethyl often enhances the chemical and metabolic stability, lipophilicity, and binding selectivity.^2^ Cyanide (CN), versatile building blocks in pharmaceutical intermediates, can be smoothly transformed into functional groups like carboxylic acids, amines, amides, ketones, and aldehydes.^3^ The one-step introduction of trifluoromethyl and cyanide to alkenes, noted as cyano-trifluoromethylation, thus became a hotspot and drew general attention.^4−9^

With pioneering efforts on trifluoromethylation using the combination of transition-metal and trifluoromethylation reagents, represented by Umemoto’s reagent and Togni’s reagent, several nucleophiles were successfully involved in difunctionalization triggered by trifluoromethylation.^10−12^ To activate the CF_3_ precursors, stoichiometric Cu(I) and Fe(II) were applied as reductants. This pathway is initiated by the electron transfer from metal to CF_3_ precursors, generating highly active CF_3_ radical as a key intermediate.^13,14^ The transition-metal mediators/catalysts also could be replaced by photocatalysts, of which a broader redox-potential range enabled cheaper reagents to be the trifluoromethyl radical sources.^4,15−17^ Given the highly active and unstable nature of the CF_3_ radical,^15^ it is difficult to discriminate the alkenyl groups with different steric environments, leading to poor regio- and shape selectivities.

Lewis acids were found to affect the Togni reagent as an active CF_3_ source.^18^ Further research found that the dynamic coordination between the Togni II reagent and Cu(I) species activates Togni reagent II without extruding the free CF_3_ radical.^19,20^ The methodology using Togni reagent II, **TMS**CN (**TMS**, trimethylsilyl), and a catalytic amount of Cu(II) salt as the catalyst conducting cyano-trifluoromethylation was discovered, in which the free trifluoromethyl radical could not be detected.^21,22^ It was postulated that the coordination interaction enabled the active CF_3_ species to dock in the coordination site of the copper center, offering chances for imposing steric control upon the accessibility of active CF_3_ species toward variant olefinic moieties within substrates.

**Figure 1:**
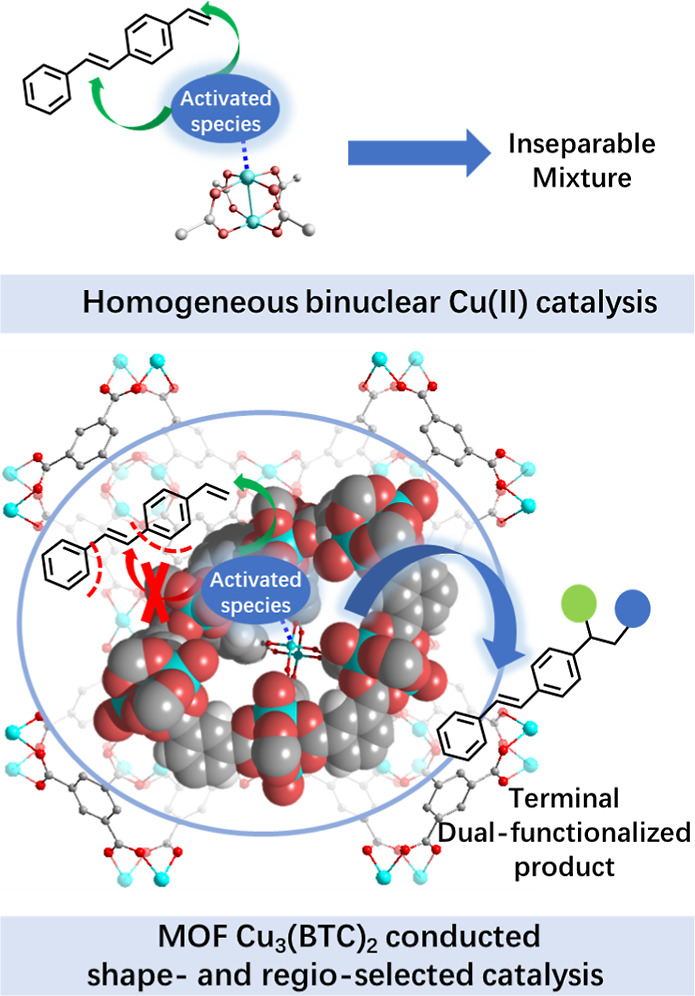
Schematic illustration of designing shape- and regioselective cyano-trifluoromethylation of alkenes by MOF Cu_3_(**BTC**)_2_ endowed by Cu(II) sites in funnel-like cavities.

Metal–organic frameworks (**MOF**s) have emerged as crystalline, porous materials with well-defined and tailorable structures,^23^ providing a platform for understanding and modulating catalysis in confined pore environments. A classic MOF Cu_3_(**BTC**)_2_ (also known as HKUST-1,^24^**BTC**, 1,3,5-benzenetricarboxylic acid) bearing the stable and accessible copper coordination sites with sterically hindered surroundings held the potential to be an effective catalyst for achieving the confinement effect within pores. In Cu_3_(**BTC**)_2_, the binuclear Cu(II) paddle-wheel nodes were densely located in the rigid 3D reticular framework. The axial coordination vacancies of copper nodes in the bottom of funnel-like cavities (Figure 1) possibly endowed the MOF catalyst with moderate Lewis acidity and radical-binding ability.^25−29^ As a result, a pronounced regio- and shape-selective cyano-trifluoromethylation was found to occur at the less steric alkenyl moieties of the substrate in this work. The examinations of the docked active CF_3_ species and other intermediates were performed to illustrate the confinement effect therein, demonstrating the origin of enhanced selectivity. The methodology could be further extended to difunctionalization reactions of alkenes with other nucleophiles besides cyanide; an azido(N_3_)-trifluoromethylation method was successfully disclosed using similar reaction conditions with Togni reagent II and **TMS**N_3_.

## 2 Results and Discussion

We began our investigation by reacting styrene **1a** with Togni reagent II and **TMS**CN. The target product, (1-cyano-3,3,3-trifluoropropyl) butylbenzene **2a**, was attained in 28% yield after 24 h under a preliminary condition (Table 1, entry 1). Considering the solubility and polarity, a set of solvent candidates was tested (entries 1–6). The nonpolar or weak polar solvents, such as 1,2-dichloride ethane (DCE), toluene, and tetrahydrofuran (THF), afforded poor yields (entries 1–3), possibly since they disfavored the cleavage of **TMS**CN, implying that the nucleophilic or ion exchange process might be involved. The polar protic solvent methanol would attack hypervalent iodine reagents as a nucleophile,^30^ thus affording an inferior yield (entry 4). Acetonitrile gave a superior yield among the polar solvents (entries 6). When checking the temperature effect, it was found that mild heating at 45 °C was necessary for initiating the reaction process (entry 7), while other higher or lower temperatures did not bring any improvement (entries 6, 8, and 9). Then, the equivalence of the **TMS**CN reagent was examined. An excess amount of **TMS**CN (1.5 equiv) was needed to reach the complete conversion and to suppress undesired nucleophiles, like a trace amount of water and 4-iodobenzoic acid, the metabolite of Togni reagent II (entries 11–13). Although a good yield of 80% was obtained in entry 7, the reaction still afforded an almost identical 79% yield of **2a** in 1 day with only a 2.5% equiv of Cu_3_(**BTC**)_2_ (entry 10). Further prolonging the reaction time to 36 h afforded a slightly elevated yield (entry 16). In comparison, more significant loadings or even substoichiometric amounts of copper salts were usually required to catalyze the trifluoromethylations in the previously reported homogeneous cases.^31−34^ The high performance of Cu_3_(**BTC**)_2_ might be attributed to the structural and functional persistence of the corresponding active sites.

**Table 1:**
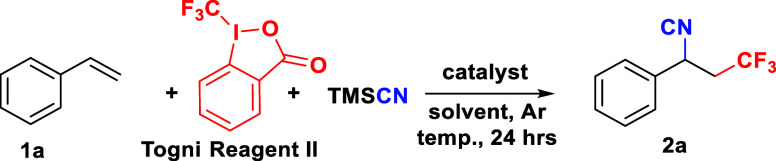
Optimization of the Reaction Conditions and Control Experiments^a^

entry	catalyst (mol %)	solvent	**TMS**CN (equiv)	temp. (°C)	yield (%)^b^
1	Cu_3_(**BTC**)_2_ (5)	DCE	1.5	65	28
2	Cu_3_(**BTC**)_2_ (5)	toluene	1.5	65	22
3	Cu_3_(**BTC**)_2_ (5)	THF	1.5	65	39
4	Cu_3_(**BTC**)_2_ (5)	CH_3_OH	1.5	65	43
5	Cu_3_(**BTC**)_2_ (5)	Et**OAc**	1.5	65	52
6	Cu_3_(**BTC**)_2_ (5)	CH_3_CN	1.5	65	65
7	Cu_3_(**BTC**)_2_ (5)	CH_3_CN	1.5	45	80
8	Cu_3_(**BTC**)_2_ (5)	CH_3_CN	1.5	25	14
9	Cu_3_(**BTC**)_2_ (5)	CH_3_CN	1.5	85	42
**10**	**Cu**_**3**_**(BTC)**_**2**_**(2.5)**	**CH**_**3**_**CN**	**1.5**	**45**	**79**
11	Cu_3_(**BTC**)_2_ (1.25)	CH_3_CN	1.5	45	61
12	Cu_3_(**BTC**)_2_ (2.5)	CH_3_CN	1.0	45	57
13	Cu_3_(**BTC**)_2_ (2.5)	CH_3_CN	2.0	45	68
variants of binuclear Cu catalyst species					
14	Cu(**OAc**)_2_ (2.5)	CH_3_CN	1.5	15	35
15	Cu(**BDC**)^c^ (2.5)	CH_3_CN	1.5	15	52
16^c^	Cu_3_(**BTC**)_2_ (2.5)	CH_3_CN	1.5	15	83

a Reaction conditions: **1a** (0.4 mmol, 1.0 equiv), Togni reagent II (0.48 mmol, 1.2 equiv), **TMS**CN (specified amount), catalyst (specified amount calculated based on Cu), solvent (5.0 mL), under specified temperature and argon atmosphere, 24 h.

b Isolated yields.

c Reacted for 36 h.

Then, we examined several compounds with binuclear Cu(II) motifs to illustrate the structure–activity relationship of catalysts. Cu(**OAc**)_2_ (cupric acetate), a single-molecular catalyst bearing a similar binuclear copper paddle-wheel structure, was used as a homogeneous control for Cu_3_(**BTC**)_2_. 2.5% equiv of Cu(**OAc**)_2_ loading led to a 35% yield of **2a** (entry 14). Next, we adopted the coordination polymer Cu(**BDC**) (**BDC**, phthalic acid) with a stacked two-dimensional layered structure, giving a moderate yield of 52% (entry 15). However, the Cu(**BDC**) particles gradually decomposed during the reaction and could not be recovered. These results demonstrated that the rigid 3D reticular and stable coordination environment of Cu sites was essential for the activity and durability of catalysts.

Under the optimized conditions, the substrate scope of MOF-catalyzed cyano-trifluoromethylation was investigated. The results are summarized in Table 2. In the presence of 2.5 mol % of Cu_3_(**BTC**)_2_, styrenes **1a** to **1e** bearing various substituents on the *para*-positions of aromatic rings were smoothly transformed into the corresponding difunctionalized products in good to excellent yields (up to 92%), regardless of their electron-donating or -withdrawing effects (**2a–2e**). Besides the aryl alkene, the terminal alkyl alkenes **1l** and **1m** were also compatible with the conditions and converted to the corresponding products in excellent yields. Inner-ring alkene (**1n**) afforded a medium yield (56% yield). 2,3-Dimethyl-2-butene (**1o**) with four methyl groups in the vicinity did not furnish separable target products. To certify its potential application in the pharmaceutical field, an estrone derivative, 3-deoxy-3-vinylestrone (**1k**), was tested as the substrate, producing the cyano-trifluoromethylation product with a good yield (**2k**).

**Table 2:**
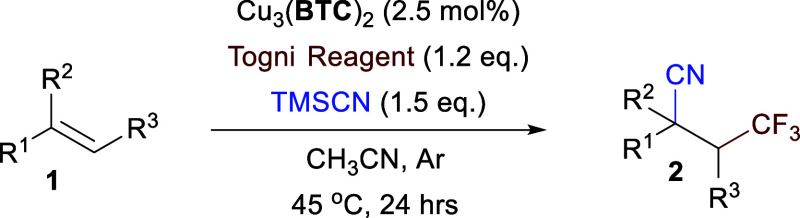

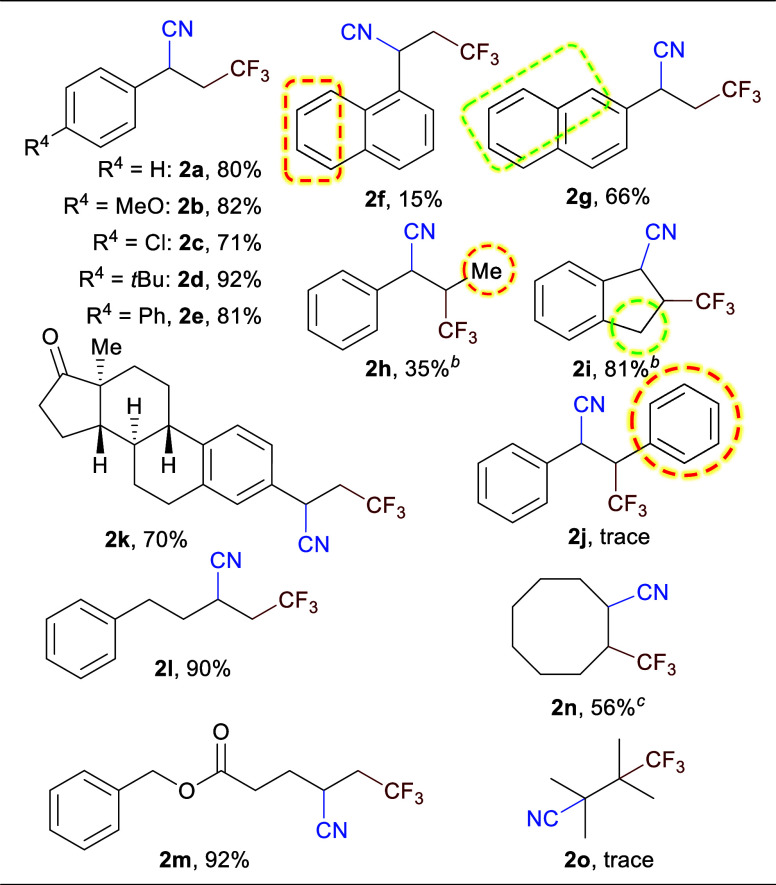
Investigations on Substrate Scope of the Reaction^a^

a Under the optimal reaction conditions shown in Table 1, entry 10. Isolated yields. Diastereoselectivity (diastereo ratio, dr) was determined by ^19^F NMR of crude products.

b dr > 20:1.

c dr = 2.25:1.

**Figure 2:**
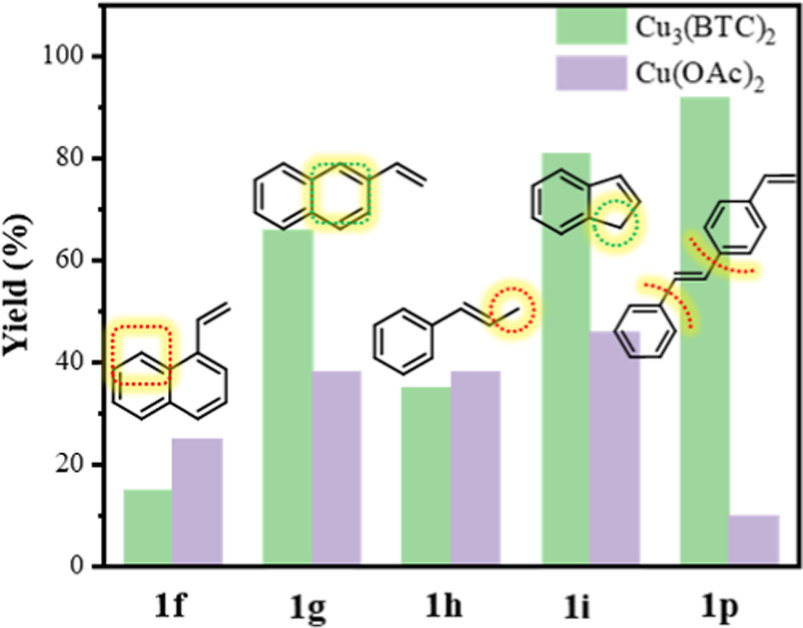
Correlation of catalyst activity with the steric environment around the alkene moieties. The dashed frames mark the surrounding steric moieties.

**Figure 3:**
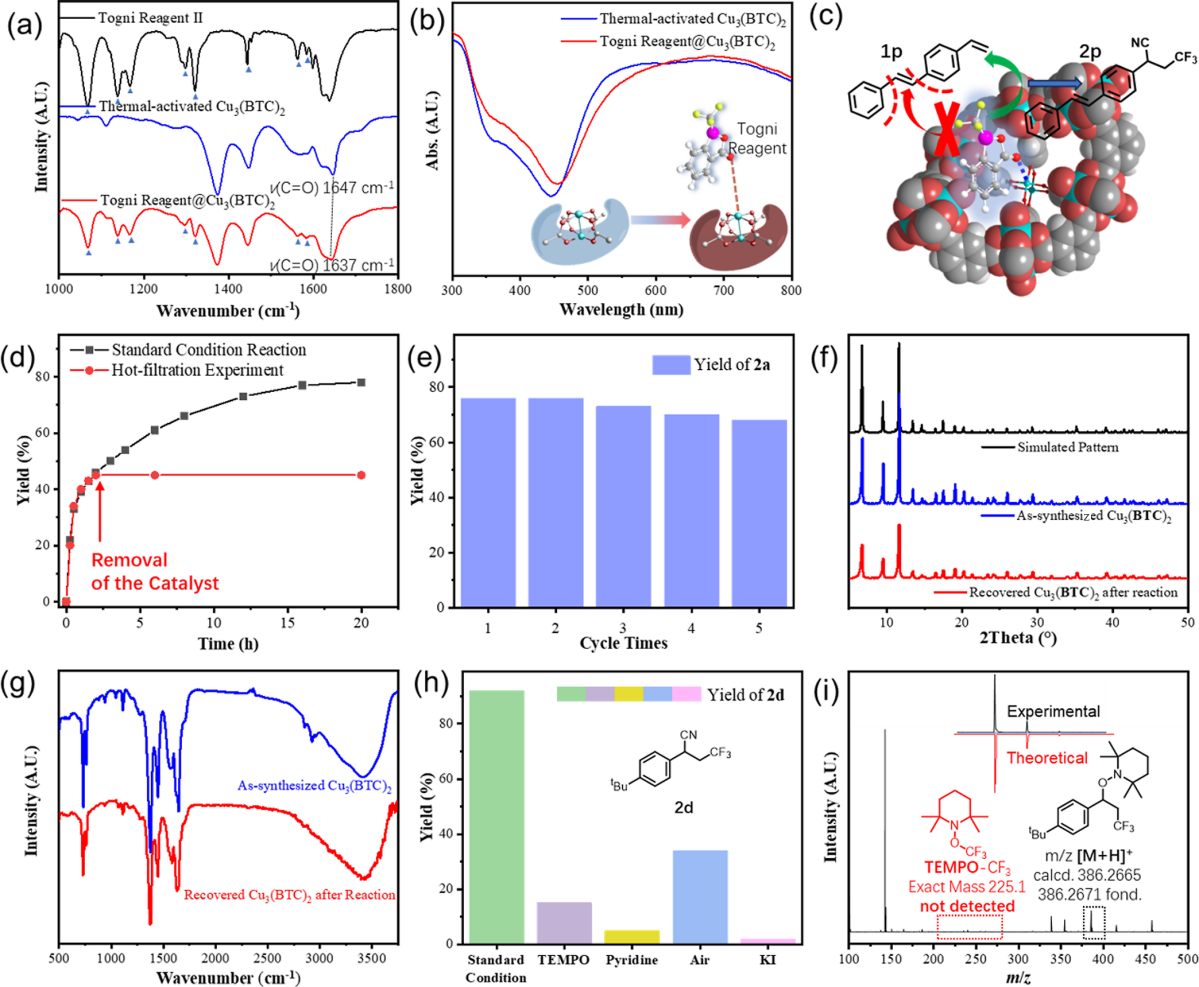
(a) FT-IR spectra of Togni reagent II, thermal-activated Cu_3_(**BTC**)_2_, and Togni reagent@Cu_3_(**BTC**)_2_. (b) UV–vis absorption spectra of thermally activated Cu_3_(**BTC**)_2_ and Togni reagent@Cu_3_(**BTC**)_2_. (c) Illustration of the origin of regio- and shape selectivities. (d) Time-coursed reaction kinetics and catalyst hot-filtration experiment. (e) Catalyst recycle experiment. (f) Powder X-ray diffractogram (PXRD) spectra of simulated, as-synthesized, and recovered Cu_3_(**BTC**)_2_ samples. (g) Comparative FT-IR spectra of as-synthesized and recovered Cu_3_(**BTC**)_2_. (h) Interference experiments in the presence of additives. (i) HRMS spectrum of the **TEMPO**-trapping experiment.

To validate the shape selectivity of Cu_3_(**BTC**)_2_ catalysis, alkene substrates bearing substituent groups with different steric effects were examined (Figure 2). Interestingly, 1-vinyl naphthalene (**1f**) and 2-vinyl naphthalene (**1g**) underwent the reactions with yields that varied dramatically from 15% (**2f**) to 66% (**2g**). Given the hindrance on the 1-position of the naphthalene ring, it was speculated that the activity of the catalyst was susceptible to the steric hindrance around the vinyl group of substrates. When *trans*-β-methylstyrene (**1h**) was used as the substrate, a 35% yield (**2h**) was obtained, which was obviously lower in comparison with the nonsubstituted styrene (**1a**, 80% yield) and indene (**1i**, 81% yield) bearing the inner-ring alkenyl groups. The inner-ring methylene (−CH_2_−) of indene (**1i**) was evidently less bulky than the freely rotating methyl group in **1h**. When *trans*-1,2-diphenyl ethene (**1j**), of which bulky phenyl groups on both sides shielded the alkene group, was used as a substrate, only a trace amount of the target product was detected. The copper centers settled on the bottom of funnel-like cavities in Cu_3_(**BTC**)_2_, which was visualized in Figure 3c, much like the binding pocket of an enzyme, favoring the accessibility of hindrance-free terminal alkenes. To utilize this property and realize regio- and shape-selective difunctionalization, 1-ethenyl-4-[(1*E*)-2-phenylethenyl] benzene (**1p**) was chosen as the model substrate for the proof of concept, which possessed both terminal and internal alkenyl moieties. A single terminal cyano-trifluoromethylation product was obtained with a good yield (**2p**, 84%), manifesting that the Cu_3_(**BTC**)_2_ conducted the reaction in a specific manner. The heterogeneous Cu_3_(**BTC**)_2_ exposing only axial coordination sites with moderate Lewis acidity mildly promoted the transition of the less bulky terminal alkenes but was more inert for those with more significant steric hindrance, which showed remarkable regio- and shape selectivity for the cyno-trifluoromethylation of alkenes and demonstrated the confined effect in the MOF cavity, further supplying clues to a deeper insight into the reaction mechanism. In comparison, the reactivity of homogeneous Cu(**OAc**)_2_ bearing isostructural Cu(II) sites but no steric surroundings was also checked by using the dual olefinic model substrate, and the resulting chaotic reaction revealed their indiscriminate accessibility toward the olefinic sites with varied steric factors (Figure 2, purple column).

The precedent research on the reaction involving Cu(II) catalysts implied that the activation of Togni reagent II occurred during its coordination with Cu(II) sites.^19,35^ However, there has not been direct observation of the activation mode. Thanks to the intrinsic porosity and the structural persistence of the coordination environment of the Cu(II) center in Cu_3_(**BTC**)_2_, we performed the in situ study on the activation of the Togni II reagent. The FT-IR spectra of the Togni II reagent-encapsulated Cu_3_(**BTC**)_2_ showed a 10 cm^–1^ red shift of the C=O stretching vibration adsorption band compared with the thermal-activated pristine Cu_3_(**BTC**)_2_ (Figures 3a and S1), indicating a dynamic coordinative equilibrium of the Togni reagent to the vacant coordination site of the paddle-wheel Cu(II) center in Cu_3_(**BTC**)_2_. The *d*–*d* transition UV–vis absorption band of Cu(II) could represent the coordination environment of the Cu(II) species.^36,37^ Thus, the UV–vis absorption spectra of thermally activated and Togni reagent II-encapsulated Cu_3_(**BTC**)_2_ were compared (Figure 3b), and the *d*–*d* transition peak of Togni reagent II-encapsulated Cu_3_(**BTC**)_2_ red-shifted compared with the thermal-activated catalyst. These facts demonstrated that the Togni II reagent occupied and was activated by the axial coordination site of the Cu(II) sites. In a classic electron-transfer activation process, Togni reagent II decomposed and sequentially released a free CF_3_ radical.^18^ However, the activation of Cu_3_(**BTC**)_2_ did not generate the CF_3_ radical that would undermine the selectivity, as all the characteristic signals of the Togni reagent were well-retained in the FT-IR spectrum of the Togni reagent-encapsulated Cu_3_(**BTC**)_2_ sample (Figure 3a, depicted by blue triangles). This docking-and-activating manner of the CF_3_ precursor could impose remarkable steric effects on the CF_3_-addition step, to endow the reaction with the regio- and shape selectivities (Figure 3c).

To prove that Cu_2_(**BTC**)_3_ was an authentic heterogeneous catalyst, control experiments were performed to evaluate the Cu_3_(**BTC**)_2_ catalyst before and after the reaction, as shown in Figure 3d–g. Removal of MOF particles by hot filtration after 2 h shut down the reaction immediately. A negligible amount of copper residue in the filtrate did not drive further reaction (Figure 3d). The catalyst could be collected and reused for recycling experiments up to 5 times without significant activity loss, further disclosing the critical role of structural rigidity in the persistent catalytic performance (Figure 3e). X-ray diffraction patterns and FT-IR spectra of catalyst samples before and after the reactions were almost identical (Figure 3f,g), indicating the maintained structural integrity of Cu_3_(**BTC**)_2_.

Control experiments were implemented to gain further insights into the mechanism of this heterogeneous cyano-trifluoromethylation (Figure 3h). When 1.2 equiv of 2,2,6,6-tetramethyl-1-piperidinyloxy (**TEMPO**) was added, the target product **2d** was still obtained in an attenuated yield. However, no radical-trapping adduct **TEMPO**-CF_3_ could be detected by HRMS and ^19^F NMR; instead, a carbon-centered radical intermediate from CF_3_-alkene addition was trapped by **TEMPO**, as shown in the HRMS spectrum (Figure 3i). These results led us to rethink the real intermediates in this reaction. When a coordination-competitive inhibitor, pyridine, was added, the reaction was seriously aggravated, suggesting the significance of coordination sites of Cu(II) centers for triggering the reaction. The air-atmosphere control experiment manifested that the existence of oxygen might disturb the radical process. The addition of potassium iodide (KI) also deteriorated the reaction, possibly because KI would be transformed into iodine under the oxidizing conditions, which was a radical pathway inhibitor.^38^ In the pioneering results on trifluoromethylation concerning Cu(II) catalysts and the Togni reagent, similar phenomena were also observed, for which a copper-assisted radical or ionized mechanism was proposed because the key intermediate of the free-radical mechanism, CF_3_ radical, that can be detected could not sufficiently promote the reaction in those systems.^21,39^ In the Cu_3_(**BTC**)_2_ catalyzed system, the results suggested that the reaction was majorly brought about via radical intermediates.

**Scheme 1:**
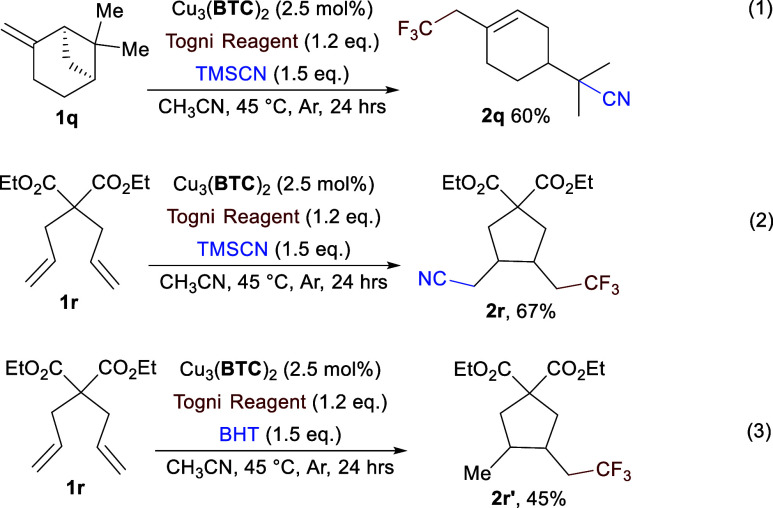
Control Experiments (1) Ring-opening reaction of (−)-β-Pinene **1q**. (2) Radical-clock reaction of **1r**. (3) **BHT** as hydrogen donor in trifluoromethylation–cyclization reaction of **1r** (isolated yields).

Given the optimized reaction conditions, (−)-β-pinene (**1q**) afforded the difunctionalization product accompanied by the ring-opening rearrangement in a moderate yield (**2q**, Scheme 1, entry 1). When intramolecular diene **1r** was employed as the substrate, the cyclized radical-clock difunctionalization was furnished in a good yield (Scheme 1, entry 2). These facts suggested that unlike the ionized mechanism, the CF_3_ group attached to the alkene to form radical intermediates, which was in accordance with the result of the **TEMPO**-trapping experiment (Figure 3i). Considering that the cation intermediates would undergo similar rearrangements, 2,6-di-*tert*-butyl-4-methyl phenol (**BHT**) was used as a hydrogen atom donor to probe the possibly existing radical intermediates. When 1.5 equiv of **BHT** instead of **TMS**CN was added in the **1r** reaction, cyclized hydrogen-trifluoromethylation product **2r′** was yielded (Scheme 1, entry 3). These results further demonstrated that the reaction majorly experienced the radical addition from the Cu-activated Togni reagent II and sequential radical cascades to form the target products.

**Scheme 2:**
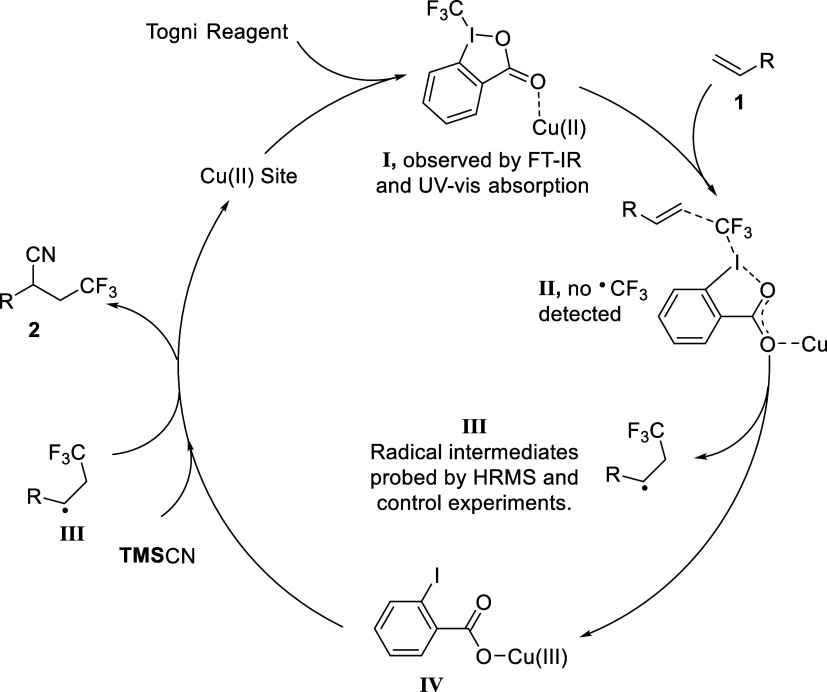
Proposed Mechanism of Cyano-Trifluoromethylation of Alkene Catalyzed by Cu_3_(**BTC**)_2_

By comparing our results with former works in similar catalytic conditions, a proposed mechanism was outlined in Scheme 2. The carbonyl group of Togni II dynamically attached to the axial coordination vacancy of the paddle-wheel Cu(II) center in Cu_3_(**BTC**)_2_ (Scheme 2, intermediate **I**), leading the reaction to proceed in an enzyme-like confined environment, endowing the catalyst with steric effect-dependent regio- and shape selectivity. When the alkene substrate (**1**) was added, the coordination interaction with carboxyl, activating Togni reagent II as a docked CF_3_ precursor (intermediate **II**), led to CF_3_ addition to the alkene, forming a carbon-centered radical mediate (intermediate **III**) and a coordinated 2-iodobenzoic acid,^18^ simultaneously, with minimal leakage of the free CF_3_ radical. At the same time, the Cu(II) center was oxidized to Cu(III) (intermediate **IV**). The CF_3_-incorporated radical mediates were sequentially oxidized and attacked by the nucleophilic cyano moiety of **TMS**CN, resulting in the final cyano-trifluoromethylation product **2**, with trimethylsilyl 2-iodobenzoate as a side product. The perfect monodispersion and structural persistence of dinuclear paddle-wheel Cu(II) nodes were maintained by the rigid 3D framework of Cu_3_(**BTC**)_2_, which avoided the irreversible ligandolysis with deteriorated reactivities to depress the non-shape-selective radical pathway. Moreover, the bottom of the funnel-shaped cavities around the copper sites imposed an additional steric demand on substrates and reaction intermediates, exhibiting the confinement effect.

**Scheme 3:**
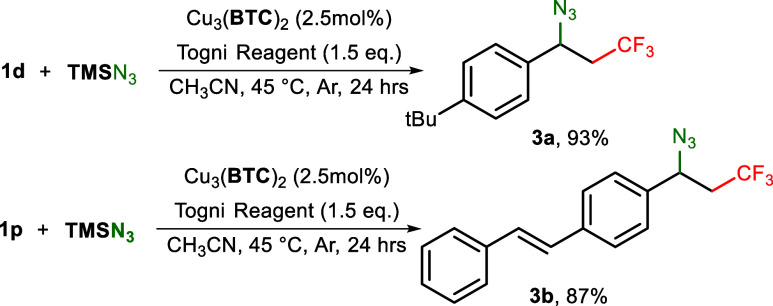
Cu_3_(**BTC**)_2_ Promoted Azido-Trifluoromethylation (Isolated Yields)

Furthermore, we extended our MOF-catalyzed difunctionalization method to azide-trifluoromethylation of alkenes. With **TMS**N_3_ as an alternative source of nucleophile, styrene derivative **1d** smoothly transformed into target product **3a** (Scheme 3, entry 1). When **1p** was tested as a substrate, a single terminal difunctionalized product **3b** was obtained with a good yield (Scheme 3, entry 2), manifesting that the regio- and shape selectivity was maintained in the Cu_3_(**BTC**)_2_ when promoting azido-trifluoromethylation.

## 3 Conclusions

In this work, we developed a heterogeneous and sustainable MOF-catalyzed cyano-trifluoromethylation method of alkenes. The MOF Cu_3_(**BTC**)_2_ with a stable 3D network and Cu(II) sites in funnel-like cavities promoted the reaction with high performance and regio- and shape selectivity. The Cu(II) sites activated the CF_3_ precursor and Togni reagent, imposed steric control on the addition of docked CF_3_ to the alkenes, and initiated sequential radical transformations without leaking CF_3_ free radicals that would undermine the selectivity. The catalyst was recyclable with little activity loss for up to five cycles. The method could be extended to other nucleophiles; here we showed **TMS**CN and **TMS**N_3_ as two examples, making the method applicable to broader-scoped radical difunctionalization of alkenes with potential pharmaceutical interests.

## 4 Experimental Section

### 4.1 Materials and Methods

All reagents were obtained from commercial sources and used without further purification. All of the solvents involved were dehydrated and degassed before use. Cu_3_(**BTC**)_2_^40^ and Cu(**BDC**)^41^ were prepared and activated with a vacuum-heating procedure according to the literature.

NMR spectra were measured on Bruker ADVANCE 500 WB and Bruker ADVANCE 400 WB spectrometers, and chemical shifts were recorded in parts per million (ppm, δ). PXRD measurements were performed with a PANalytical Empyrean powder X-ray diffractometer (Cu Kα radiation, 40 kV, 40 mA). FT-IR spectra were recorded as KBr pellets on a JASCO FT/IR-430. Solid-phase UV–vis adsorption spectra were recorded on a HITACHI U-4100 spectrophotometer.

### 4.2 Experimental Procedures and Characterization of Data

#### 4.2.1 General Procedure for Heterogeneous Catalysis by Cu_3_(**BTC**)_2_

Togni reagent II (0.48 mmol, 1.2 equiv) and Cu_3_(**BTC**)_2_ (0.01 mmol) were added to a Schlenk tube. The tube was evacuated and back-filled with argon three times. Then anhydrous CH_3_CN (5.0 mL), alkenes (0.4 mmol, 1.0 equiv), and **TMS**CN (0.6 mmol, 1.5 equiv) were injected successively. The reaction mixture was stirred at 45 °C for 24 h. The catalyst was removed by filtration and washed with CH_3_CN. The filtrate was concentrated under vacuum and purified by flash column chromatography on silica gel. The yields were determined by the isolated products.

The reactions catalyzed by other kinds of (or other specified amounts of) copper-containing heterogeneous/homogeneous species were conducted in a similar manner.

## Supporting Information Available

Instructions on material synthesis, experiment details, additional figures, characterization data of compounds, and NMR spectra (PDF)

## Abbreviations

TMS: trimethylsilyl
MOFs: metal–organic frameworks
**BTC**: 1,3,5-benzenetricarboxylic acid
**BDC**: phthalic acid
BHT: 2,6-di-*tert*-butyl-4-methyl phenol
