# Supplementary material for: Confinement Effect in Metal–Organic Framework Cu3(BTC)2 for Enhancing Shape Selectivity of Radical Difunctionalization of Alkenes
Source: ACS Omega. 2024 Mar 11;9(12):14233–40. doi: 10.1021/acsomega.3c09911 (PMC10976352; doi:10.1021/acsomega.3c09911)
Supplement: Supplementary file 1 — ao3c09911_si_001.pdf [file ao3c09911_si_001.pdf]

Supporting Information

Confinement effect in metal-organic framework $Cu_3(BTC)_2$ for enhancing shape-selectivity of radical difunctionalization of alkenes

Mochen Li,[a] Zhi Feng,[a] Chunying Duan,[a,b] Tiexin Zhang*[a], and Yusheng Shi*[c].

[a]State Key Laboratory of Fine Chemicals, School of Chemical Engineering, School of Chemistry, Dalian University of Technology, Dalian 116024, China.

[b]State Key Laboratory of Coordination Chemistry, Nanjing University, Nanjing 210023, P. R. China.

[c]Jiangsu Yangnong Chemical Group Co., Ltd., Yangzhou 225001, P. R. China.

*Corresponding author;

E-mail address: zhangtiexin@dlut.edu.cn; yushengshi@dlut.edu.cn.

Contents

Characteristic Spectra of the Material 2

Experiment section 2

Materials and methods 2

Synthetic procedure of Cu_3(**BTC**)$_2$ 2

Synthetic procedure of Cu(**BDC**) 2

General procedure (GP) for heterogeneous catalysis by Cu_3(**BTC**)$_2$ 3

Characterization data of compounds 3

NMR Spectra of Compounds. 8

References. 36

Characteristic Spectra of the Material

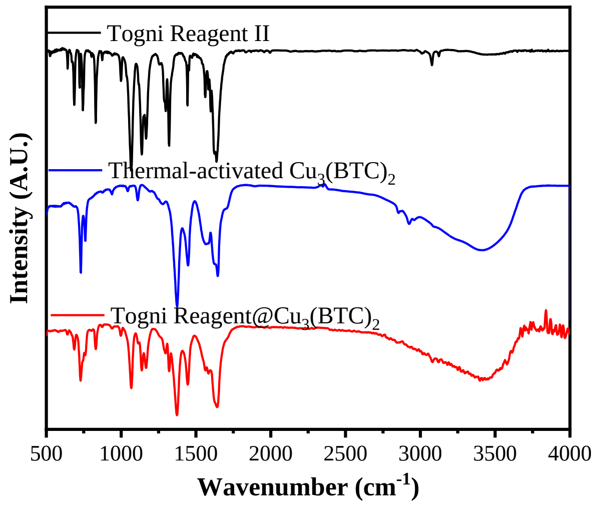

Figure S1 Comparative FT-IR spectra of Togni reagent II, Thermal activated Cu_3(**BTC**)$_2$, and Togni Reagent@Cu_3(**BTC**)$_2$.

Experiment section

Materials and methods

All reagents were obtained from commercial sources and used without further purification. All the solvents involved were dehydrated and degassed before use. Cu_3(**BTC**)$_2$ and Cu(**BDC**) were prepared according to literature.[1,2] The MOFs were activated by heating at 80°C for 24 hrs under vacuum.

NMR spectra were measured on Bruker Advance 500 WB, and Bruker Advance 400 WB spectrometers and chemical shifts were recorded in parts per million (ppm, δ). Powder X-ray diffractograms (PXRD) measurements were performed with a PANalytical Empyrean X-ray powder diffractometer (Cu Kα radiation, 40 kV, 40 mA). FT-IR spectra were recorded as KBr pellets on JASCO FT/IR-430. Solid-phase UV-vis adsorption spectra were recorded on a HITACHI U-4100 spectrophotometer.

Synthetic procedure of Cu_3(BTC)$_2$

The synthesis of Cu_3(**BTC**)$_2$ was slightly deviated from the reported procedure.[1] Copper acetate hydrate (Cu(O**Ac**)$_2$·H_2O; 34.5 mg, 0.17 mmol) and dodecanoic acid (1.4276 g, 7.125 mmol) were dissolved in 5 mL butanol. The mixture was stirred to obtain a clear solution. Benzene-1,3,5-tricarboxylic acid (20 mg, 0.095 mmol) was added at room temperature, and the sealed reaction mixture was then placed in the oven and heated to 140 °C at an increasing rate of 20°C/h and maintained at 140 °C for 5 hrs. The resulting blue powder was isolated by centrifugation and washed with ethanol by three dispersion−sonication−centrifugation cycles. The resulting light blue solid was dried under vacuum at 80 °C before use.

Synthetic procedure of Cu(BDC)

The synthesis of Cu(**BDC**) was in reference to a former literature.[2] Copper nitrate trihydrate (Cu(NO_3)$_2$·3H_2O; 1.053 g, 4.36 mmol) and terephthalic acid (0.724 g, 4.36 mmol) were dissolved in 87 mL DMF. This solution was placed in an oven at 110 °C for 36 h. The resulting blue powder was isolated by centrifugation and washed with ethanol by three dispersion−sonication−centrifugation cycles. The resulting blue solid was dried under vacuum at 80 °C before use.

General procedure (GP) for heterogeneous catalysis by $Cu_3(BTC)_2$

Togni reagent II (151.6 mg, 0.48 mmol, 1.2 equiv.) and Cu_3(**BTC**)$_2$ (2.0 mg, 0.01 mmol, 2.5 mol%; calculated according to the amount of Cu sites) were added to a Schlenk tube. The tube was evacuated and backfilled with Argon (repeated three times). Then anhydrous CH_3CN (5.0 mL) and alkenes (0.4 mmol, 1.0 equiv.), and **TMS**CN (59.4 mg, 76 μL, 0.6 mmol, 1.5 equiv.) were injected successively. The reaction mixture was stirred at 45 °C for 24 hrs. The catalyst was removed by filtration and washed with CH_3CN. The filtrate was concentrated under vacuum and purified by flash column chromatography on silica gel. The yields were determined by the isolated products.

The reactions catalyzed by other kinds of (or other specified amounts of) copper-containing heterogeneous/homogeneous species were conducted in similar manners.

Characterization data of compounds

4,4,4-trifluoro-2-phenylbutanenitrile (2a)

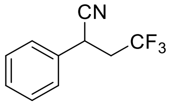

This compound was synthesized according to the GP and isolated by column chromatography as colorless oil (80%) using petroleum ether as the eluent. ^{1}H NMR (500 MHz, $CDCl_3$) δ 7.46 – 7.35 (m, 5H), 4.10 (dd, J = 9.4, 5.1 Hz, 1H), 2.91 – 2.76 (m, 1H), 2.60 (ddd, J = 15.0, 9.9, 5.2 Hz, 1H). ^{13}C NMR (101 MHz, $CDCl_3$) δ 133.6, 129.7, 129.2, 127.3, 124.8 (q, J = 277.9 Hz), 118.7, 40.0 (q, J = 29.6 Hz), 31.4 (q, J = 2.9 Hz). ^{19}F NMR (470 MHz, $CDCl_3$) δ -65.08 (t, J = 10.6 Hz). The NMR data were in accordance with the data in the literature.[3] HRMS (EI): $[M]^+$: Calculated: 199.0609; Found: 199.0602.

4,4,4-trifluoro-2-(4-methoxyphenyl)butanenitrile (2b)

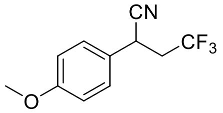

This compound was synthesized according to the GP and isolated by column chromatography as colorless oil (82%) using petroleum ether/ethyl acetate (V:V = 50:1) as the eluent. ^{1}H NMR (400 MHz, $CDCl_3$) δ 7.28 (d, J = 8.7 Hz, 2H), 6.93 (d, J = 8.7 Hz, 2H), 4.05 (dd, J = 9.3, 5.3 Hz, 1H), 3.82 (s, 3H), 2.81 (dp, J = 15.1, 9.6 Hz, 1H), 2.56 (dqd, J = 15.1, 9.9, 5.4 Hz, 1H). ^{13}C NMR (101 MHz, $CDCl_3$) δ 160.1 , 128.5 , 125.5 , 124.8 (d, J = 276.8 Hz), 119.0, 115.0, 55.6 , 40.0 (q, J = 29.4 Hz), 30.7 (q, J = 3.2 Hz). ^{19}F NMR (470 MHz, $CDCl_3$) δ -65.04 (t, J = 9.8 Hz). The NMR data were in accordance with the data in the literature.[3] HRMS (EI): $[M]^+$: Calculated: 229.0714; Found: 229.0711.

2-(4-chlorophenyl)-4,4,4-trifluorobutanenitrile (2c)

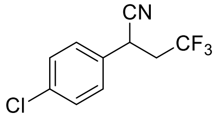

This compound was synthesized according to the GP and isolated by column chromatography as colorless oil (71%) using petroleum ether/ethyl acetate (V:V = 50:1) as the eluent. ^{1}H NMR (500 MHz, $CDCl_3$) δ 7.42 (d, J = 8.6 Hz, 2H), 7.32 (d, J = 8.5 Hz, 2H), 4.09 (dd, J = 9.1, 5.5 Hz, 1H), 2.83 (dp, J = 15.1, 9.5 Hz, 1H), 2.59 (dqd, J = 15.1, 9.7, 5.4 Hz, 1H). ^{13}C NMR (126 MHz, $CDCl_3$) δ 135.4, 132.0, 130.0, 128.8, 124.6 (d, J = 277.5 Hz), 118.3, 39.8 (q, J = 29.9 Hz), 31.0 (q, J = 3.1 Hz). ^{19}F NMR (470 MHz, $CDCl_3$) δ -64.93 (t, J = 9.6 Hz). The NMR data were in accordance with the data in the literature.[3] HRMS (EI): $[M]^+$: Calculated: 233.0219; Found: 233.0225.

2-(4-(*tert*-butyl)phenyl)-4,4,4-trifluorobutanenitrile (2d)

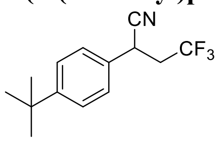

This compound was synthesized according to the GP and isolated by column chromatography as colorless oil (92%) using petroleum ether as the eluent. ^{1}H NMR (400 MHz, $CDCl_3$) δ 7.44 (d, J = 8.3 Hz, 2H), 7.30 (d, J = 8.3 Hz, 2H), 4.08 (dd, J = 9.8, 4.9 Hz, 1H), 2.82 (dp, J = 15.1, 9.7 Hz, 1H), 2.59 (dqd, J = 14.9, 9.9, 4.9 Hz, 1H), 1.33 (s, 9H). ^{13}C NMR (126 MHz, $CDCl_3$) δ 152.4, 130.6, 127.0, 126.6, 124.8 (q, J = 277.8 Hz), 118.9, 39.9 (q, J = 29.5 Hz), 34.8, 31.3,

30.9 (q, J = 3.2 Hz). ^{19}F NMR (470 MHz, $CDCl_3$) δ -65.17 (t, J = 9.8 Hz). The NMR data were in accordance with the data in the literature.[3] HRMS (EI): $[M]^+$: Calculated: 255.1235; Found: 255.1236.

2-([1,1'-biphenyl]-4-yl)-4,4,4-trifluorobutanenitrile (2e)

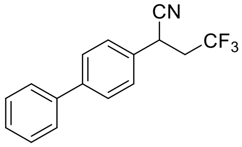

This compound was synthesized according to GP and isolated by column chromatography as colorless oil (81%) using petroleum ether/ethyl acetate (V:V = 50:1) as the eluent. ^{1}H NMR (400 MHz, $CDCl_3$) δ 7.65 (d, J = 8.2 Hz, 2H), 7.59 (d, J = 7.4 Hz, 2H), 7.49 – 7.43 (m, 4H), 7.39 (t, J = 7.2 Hz, 1H), 4.15 (dd, J = 9.5, 5.1 Hz, 1H), 2.88 (dp, J = 15.1, 9.6 Hz, 1H), 2.73 – 2.56 (m, 1H). ^{13}C NMR (126 MHz, $CDCl_3$) δ 142.3, 140.0, 132.5, 129.1, 128.4, 128.0, 127.8, 127.3, 124.8 (q, J = 277.9 Hz), 118.7, 39.9 (q, J = 29.7 Hz), 31.1 (q, J = 3.3 Hz). ^{19}F NMR (470 MHz, $CDCl_3$) δ -65.00 (t, J = 9.8 Hz). The NMR data were in accordance with the data in the literature.[3] HRMS (EI): $[M]^+$: Calculated: 275.0922; Found: 275.0927.

4,4,4-trifluoro-2-(naphthalen-1-yl)butanenitrile (2f)

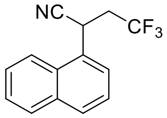

This compound was synthesized according to the GP and isolated by column chromatography as colorless oil (15%) using petroleum ether/ethyl acetate (V:V = 50:1) as the eluent. ^{1}H NMR (400 MHz, $CDCl_3$) δ 7.97 – 7.93 (m, 1H), 7.91 (d, J = 8.3 Hz, 1H), 7.87 (d, J = 8.5 Hz, 1H), 7.76 – 7.74 (m, 1H), 7.65 (ddd, J = 8.4, 6.9, 1.4 Hz, 1H), 7.58 (ddd, J = 8.0, 6.9, 1.1 Hz, 1H), 7.52 (dd, J = 8.2, 7.3 Hz, 1H), 4.85 (dd, J = 10.5, 3.6 Hz, 1H), 2.92 (ddq, J = 19.3, 15.2, 9.6 Hz, 1H), 2.80 – 2.67 (m, 1H). ^{13}C NMR (126 MHz, $CDCl_3$) δ 134.4, 130.2, 129.8, 129.4, 129.1, 127.8, 126.7, 126.1, 125.7, 125.0 (q, J = 277.9 Hz), 121.4, 119.0, 39.0 (q, J = 29.5 Hz), 28.4 (q, J = 3.3 Hz). ^{19}F NMR (470 MHz, $CDCl_3$) δ -65.51 (t, J = 9.8 Hz). The NMR data were in accordance with the data in the literature.[3] HRMS (EI): $[M]^+$: Calculated: 249.0765; Found: 249.0768.

4,4,4-trifluoro-2-(naphthalen-2-yl)butanenitrile (2g)

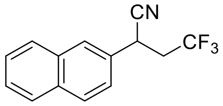

This compound was synthesized according to the GP and isolated by column chromatography as colorless oil (66%) using petroleum ether/ethyl acetate (V:V = 50:1) as the eluent. ^{1}H NMR (400 MHz, $CDCl_3$) δ 7.92 (d, J = 8.5 Hz, 1H), 7.90 – 7.84 (m, 3H), 7.61 – 7.52 (m, 2H), 7.42 (dd, J = 8.5, 2.0 Hz, 1H), 4.27 (dd, J = 9.6, 5.1 Hz, 1H), 2.92 (dp, J = 15.1, 9.6 Hz, 1H), 2.70 (dqd, J = 15.0, 9.9, 5.1 Hz, 1H). ^{13}C NMR (126 MHz, $CDCl_3$) δ 133.4, 133.3, 130.7, 129.9, 128.1, 128.0, 127.3, 127.2, 126.8, 124.8 (q, J = 277.7 Hz), 124.2, 118.7, 39.9 (q, J = 29.6 Hz), 31.6 (q, J = 3.2 Hz). ^{19}F NMR (470 MHz, $CDCl_3$) δ -65.01 (t, J = 9.7 Hz). The NMR data were in accordance with the data in the literature.[3] HRMS (EI): $[M]^+$: Calculated: 249.0765; Found: 249.0759.

4,4,4-trifluoro-3-methyl-2-phenylbutanenitrile (2h)

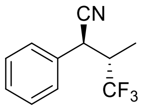

This compound was synthesized according to the GP and isolated as by column chromatography as colorless oil (35%) using petroleum ether as the eluent. Only one set of diastereoisomer could be obtained. ^{1}H NMR (400 MHz, $CDCl_3$) δ 7.55 – 7.30 (m, 5H), 4.32 (d, J = 3.3 Hz, 1H), 2.65-2.46 (m, 1H), 1.27 (d, J = 7.1 Hz, 3H). ^{13}C NMR (101 MHz, $CDCl_3$) δ 133.0, 129.5, 129.0, 127.7, 126.5 (q, J = 280.4 Hz), 117.1, 43.8 (q, J = 27.1 Hz), 37.2 (q, J = 2.8 Hz), 9.2 (q, J = 2.4 Hz). ^{19}F NMR (377 MHz, $CDCl_3$) δ -71.93 (d, J = 8.4 Hz). The NMR data were in accordance with the data in the literature.[4] HRMS (EI): $[M]^+$: Calculated: 213.0765; Found: 213.0763.

2-(trifluoromethyl)-2,3-dihydro-1*H*-indene-1-carbonitrile (2i)

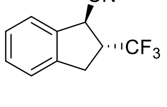

This compound was synthesized according to the GP and isolated by column chromatography as colorless oil (81%) using petroleum ether as the eluent. Only one set of diastereoisomer could be obtained. ^{1}H NMR (400 MHz, $CDCl_3$) δ 7.49 – 7.43 (m, 1H), 7.38 – 7.32 (m, 2H), 7.29 (qd, J = 4.9, 1.1 Hz, 1H), 4.35 (d, J = 8.9 Hz, 1H), 3.48 (dtt, J = 17.0, 9.0, 8.1 Hz, 1H), 3.33 (dd, J = 16.2, 8.9 Hz, 1H), 3.18 (dd, J = 16.2, 9.1 Hz, 1H). ^{13}C NMR (126 MHz, $CDCl_3$) δ 139.4, 135.1, 129.6, 128.4, 126.4 (q, J = 277.7 Hz), 125.2, 124.5, 119.0, 48.4 (q, J = 29.1 Hz), 35.7 (q, J = 3.0 Hz), 32.0 (q, J = 2.5 Hz). ^{19}F NMR (470 MHz, $CDCl_3$) δ -70.96 (d, J = 8.2 Hz). The NMR data were in accordance with the data in the literature.[5] HRMS (EI): $[M]^+$: Calculated: 211.0609; Found: 211.0606.

4,4,4-trifluoro-2,3-diphenylbutanenitrile (2j)

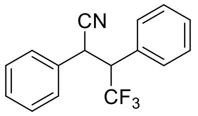

The product **2j** was not separable from the substrate (*E*)-1,2-diphenylethene (**1j**), and the NMR yield was determined from ^{1}H NMR spectrum of crude mixture, according the reported data of a similar compound.[6]

(8*R*,9*S*,13*S*,14*S*)-13-methyl-3-vinyl-6,7,8,9,11,12,13,14,15,16-decahydro-17*H*-cyclopenta[*a*]phenanthren-17-one 3-deoxy-3-vinylestrone (2k)

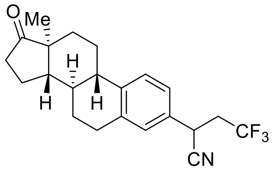

This compound was synthesized according to the GP and isolated by column chromatography as white powder (70%) using petroleum ether/ethyl acetate (V:V = 25:1) as the eluent. ^{1}H NMR (400 MHz, $CDCl_3$) δ 7.33 (d, J = 8.0 Hz, 1H), 7.16 – 7.08 (m, 2H), 4.03 (dd, J = 9.8, 4.9 Hz, 1H), 3.01 – 2.88 (m, 2H), 2.81 (dp, J = 15.1, 9.8 Hz, 1H), 2.67 – 2.47 (m, 2H), 2.47 – 2.38 (m, 1H), 2.30 (td, J = 10.4, 4.3 Hz, 1H), 2.22 – 1.92 (m, 4H), 1.73 – 1.40 (m, 6H), 0.91 (s, 3H). ^{13}C NMR (101 MHz, $CDCl_3$) δ 220.6, 141.0, 138.2, 131.0, 127.8 (d, J = 3.1 Hz), 126.7, 124.8 (q, J = 277.8 Hz), 124.6 (d, J = 3.8 Hz), 118.8 (d, J = 1.3 Hz), 50.6, 48.0, 44.4, 39.9 (qd, J = 29.4, 2.8 Hz), 38.0, 35.9, 31.6, 30.9, 29.4 (d, J = 2.7 Hz), 26.4, 25.8, 21.7, 13.9. ^{19}F NMR (377 MHz, $CDCl_3$) δ -65.12 (td, J = 9.9, 1.7 Hz,). The NMR data were in accordance with the data in the literature.[4] HRMS (EI): $[M]^+$: Calculated: 375.1810; Found: 375.1814.

4,4,4-trifluoro-2-phenethylbutanenitrile (2l)

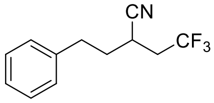

This compound was synthesized according to the GP and isolated by column chromatography as colorless oil (90%) using petroleum ether as the eluent system. ^{1}H NMR (400 MHz, $CDCl_3$) δ 7.33 (t, J = 7.4 Hz, 2H), 7.26 (d, J = 7.0 Hz, 1H), 7.20 (d, J = 7.4 Hz, 2H), 2.94 (ddd, J = 14.1, 8.7, 5.5 Hz, 1H), 2.90 – 2.73 (m, 2H), 2.54 (app dp, J = 15.2, 9.9 Hz, 1H), 2.34 (dqd, J = 15.2, 10.0, 5.3 Hz, 1H), 2.12 – 1.89 (m, 2H). ^{13}C NMR (101 MHz, $CDCl_3$) δ 139.2, 129.0, 128.5, 126.9, 125.2 (q, J = 277.3 Hz), 119.6, 36.6 (q, J = 30.1 Hz), 33.8, 32.9, 25.1 (q, J = 3.4 Hz). ^{19}F NMR (470 MHz, $CDCl_3$) δ -64.74 (t, J = 10.0 Hz). The NMR data were in accordance with the data in the literature.[7] HRMS (EI): $[M]^+$: Calculated: 227.0922; Found: 227.0920.

benzyl 4-cyano-6,6,6-trifluorohexanoate (2m)

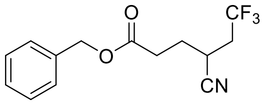

This compound was synthesized according to the GP and isolated by column chromatography as colorless oil (92%) using petroleum ether/ethyl acetate (V:V = 30:1) the eluent. ^{1}H NMR (400 MHz, $CDCl_3$) δ 7.42 – 7.32 (m, 5H), 5.15 (d, J = 2.6 Hz, 2H), 3.17 – 2.94 (m, 1H), 2.76 – 2.46 (m, 3H), 2.35 (dqd, J = 15.1, 10.0, 5.1 Hz, 1H), 2.17 – 2.03 (m, 1H), 2.02 – 1.90 (m, 1H). ^{13}C NMR (101 MHz, $CDCl_3$) δ 171.6, 135.5, 128.8, 128.6, 128.5, 125.0 (q, J = 277.3 Hz), 119.1, 67.0, 36.5 (q, J = 30.1 Hz), 31.1, 27.3, 25.0 (q, J = 3.0 Hz). ^{19}F NMR (377 MHz, $CDCl_3$) δ -64.78 (t, J = 9.9 Hz). HRMS (EI): $[M]^+$: Calculated: 285.0977; Found: 285.0982.

2-(trifluoromethyl)cyclooctane-1-carbonitrile (2n)

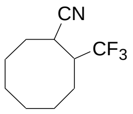

This compound was synthesized according to the GP and isolated by column chromatography as colorless oil (56%) using petroleum ether as the eluent. The mixture of diastereoisomers was obtained, with the mole ratio of the major isomer to minor isomer (d.r.) as 2.25:1. ^{1}H NMR (400 MHz, $CDCl_3$, major and minor isomers) δ 2.81 (tt, J = 8.3, 4.0 Hz, 1H), 2.33 – 2.19 (m, 1H), 2.16 – 2.05 (m, 2H), 2.02 – 1.87 (m, 2H), 1.90 – 1.71 (m, 5H), 1.68 – 1.56 (m, 1H), 1.58 – 1.39 (m, 2H). ^{13}C NMR (101 MHz, $CDCl_3$, major and minor isomers) δ 128.3 (q, J = 279.6 Hz), 122.8, 122.4, 42.3 (q, J = 25.4 Hz), 42.4 (q, J = 25.1 Hz), 29.7, 29.6, 28.9, 28.8, 27.4, 26.3, 25.9, 25.6, 24.5, 24.2, 24.0, 23.9 (q, J = 2.5 Hz), 23.1 (q, J = 2.6 Hz), 22.4. ^{19}F NMR (376 MHz, $CDCl_3$, major and minor isomers) δ -73.28 (d, J = 8.9 Hz), -73.35 (d, J = 9.3 Hz). HRMS (EI): $[M]^+$: Calculated: 205.1078; Found: 205.1071.

4,4,4-trifluoro-2,2,3,3-tetramethylbutanenitrile (2o)

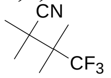

The product 2o was not separable from the substrate, and the NMR yield was determined from ^{1}H NMR spectrum of crude mixture, according the reported data of a similar compound.[8]

(*E*)-4,4,4-trifluoro-2-(4-styrylphenyl)butanenitrile (2p)

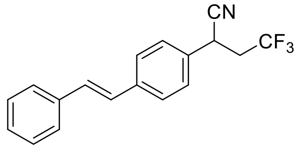

This compound was synthesized according to the GP and isolated by column chromatography as white powder (84%) using petroleum ether/ethyl acetate (V:V = 50:1) as the eluent system. ^{1}H NMR (500 MHz, $CDCl_3$) δ 7.56 (d, J = 8.2 Hz, 2H), 7.52 (d, J = 7.4 Hz, 2H), 7.42 – 7.34 (m, 4H), 7.29 (t, J = 7.3 Hz, 1H), 7.15 (d, J = 16.4 Hz, 1H), 7.09 (d, J = 16.3 Hz, 1H), 4.11 (dd, J = 9.4, 5.1 Hz, 1H), 2.85 (app dp, J = 15.2, 9.6 Hz, 1H), 2.61 (app ddp, J = 15.0, 9.8, 4.9 Hz, 1H). ^{13}C NMR (101 MHz, $CDCl_3$) δ 138.4, 137.0, 132.5, 130.4, 128.9, 128.2, 127.7, 127.6, 127.4, 126.8, 124.8 (d, J = 278.4 Hz), 118.6, 39.9 (q, J = 29.8 Hz), 31.2 (q, J = 2.7 Hz). ^{19}F NMR (470 MHz, $CDCl_3$) δ -64.98 (t, J = 9.8 Hz). HRMS (EI): $[M]^+$: Calculated: 301.1078; Found: 301.1085.

2-methyl-2-(4-(2,2,2-trifluoroethyl)cyclohex-3-en-1-yl) propanenitrile (2q)

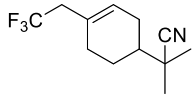

This compound was synthesized according to the GP and isolated by column chromatography as colorless oil (60%) using petroleum ether as the eluent. ^{1}H NMR (400 MHz, $CDCl_3$) δ 5.69 – 5.65 (m, 1H), 2.84 – 2.64 (m, 2H), 2.30 – 2.13 (m, 3H), 2.04 – 1.94 (m, 2H), 1.63 – 1.54 (m, 1H; overlapped with water peak in $CDCl_3$), 1.50 – 1.39 (m, 1H), 1.37 (s, 3H), 1.34 (s, 3H). ^{13}C NMR (101 MHz, $CDCl_3$) δ 134.4, 127.7, 126.2 (q, J = 277.7 Hz), 124.5, 41.7, 41.7 (q, J = 29.0 Hz), 36.0, 29.4 (q, J = 1 Hz), 27.5, 24.8, 24.6, 24.3. ^{19}F NMR (377 MHz, $CDCl_3$) δ -64.84 (t, J = 11.1 Hz). HRMS (EI): $[M]^+$: Calculated: 231.1235; Found: 231.1243.

diethyl 3-(cyanomethyl)-4-(2,2,2-trifluoroethyl)cyclopentane-1,1-dicarboxylate (2r)

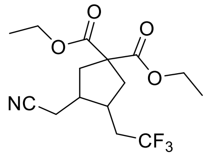

This product was obtained as a mixture of major and minor diastereoisomers (major:minor = 100:12) according to the GP and isolated by column chromatography as white powder (67%) using petroleum ether as the eluent. Characterization data for the major isomer: ^{1}H NMR (400 MHz, $CDCl_3$) δ 4.27 – 4.13 (m, 4H), 3.17 – 3.01 (m, 2H), 2.64 – 2.35 (m, 4H), 2.35 – 1.95 (m, 4H), 1.29 – 1.22 (m, 6H). ^{13}C NMR (101 MHz, $CDCl_3$) δ 172.45, 172.14, 126.87 (q, J = 277.0 Hz), 122.84, 62.06, 61.97, 58.48, 45.29, 39.80, 37.97, 36.70 (q, J = 2.2 Hz), 33.15 (q, J = 28.2 Hz), 14.17, 14.14, 5.58. ^{19}F NMR (377 MHz, $CDCl_3$) δ -64.31 (t, J = 10.8 Hz, 3F). HRMS (EI): $[M]^+$: Calculated: 335.1344; Found: 335.1349.

diethyl 3-methyl-4-(2,2,2-trifluoroethyl)cyclopentane-1,1-dicarboxylate (2r')

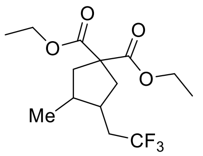

This product was obtained as a mixture of major and minor diastereoisomers (major:minor = 100:16) according to the procedure in **scheme 2**, entry 3, and isolated by column chromatography as white powder (45%) using petroleum ether as the eluent. Characterization data for the major isomer: ^{1}H NMR (400 MHz, $CDCl_3$) δ 4.22 – 4.14 (m, 4H), 2.52 – 2.40 (m, 2H), 2.37 – 2.22 (m, 2H), 2.23 – 1.77 (m, 4H), 1.27 – 1.19 (m, 6H), 0.87 (d, J = 6.3 Hz, 3H). ^{13}C NMR (101 MHz, $CDCl_3$) δ 172.74, 172.71, 127.3 (q, J = 276.9 Hz), 61.72, 61.70, 58.8, 41.3, 38.1, 36.6 (q, J = 2.2 Hz), 36.1, 34.1 (q, J = 28.0 Hz), 15.0, 14.2. ^{19}F NMR (377 MHz, $CDCl_3$) δ -64.63 (t, J = 11.0 Hz). The NMR data were in accordance with the data in the literature.[8] HRMS (EI): $[M]^+$: Calculated: 310.1392; Found: 310.1388.

1-(1-azido-3,3,3-trifluoropropyl)-4-(*tert*-butyl)benzene (3a)

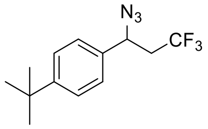

This compound was synthesized according to the GP and isolated by column chromatography as colorless oil (93%) using petroleum ether as the eluent. ^{1}H NMR (400 MHz, $CDCl_3$) δ 7.43 (d, J = 8.4 Hz, 2H), 7.25 (d, J = 8.3 Hz, 2H), 4.75 (dd, J = 8.9, 4.7 Hz, 1H), 2.62 (dqd, J = 15.2, 10.0, 8.9 Hz, 1H), 2.55 – 2.40 (m, 1H), 1.33 (s, 9H). ^{13}C NMR (101 MHz, $CDCl_3$) δ 152.3, 134.9, 126.5, 126.2, 125.5 (q, J = 277.4 Hz), 59.8 (q, J = 3.0 Hz), 40.5 (q, J = 28.2 Hz), 34.8, 31.4. ^{19}F NMR (377 MHz, $CDCl_3$) δ -64.15 (t, J = 10.3 Hz). The NMR data were in accordance with the data in the literature.[5] HRMS (EI): $[M]^+$: Calculated: 271.1296; Found: 271.1299.

(*E*)-1-(1-azido-3,3,3-trifluoropropyl)-4-styrylbenzene (3b)

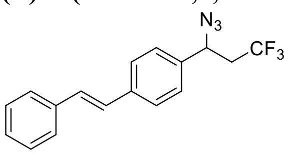

This compound was synthesized according to the GP and isolated by column chromatography as white powder (87%) using petroleum ether/ethyl acetate (V:V = 30:1) as the eluent. ^{1}H NMR (500 MHz, $CDCl_3$) δ 7.56 (d, J = 8.2 Hz, 2H), 7.53 (dd, J = 7.6, 1.6 Hz, 2H), 7.38 (t, J = 7.6 Hz, 2H), 7.34 – 7.31 (m, 2H), 7.31 – 7.27 (m, 1H), 7.15 (d, J = 16.4 Hz, 1H), 7.10 (d, J = 16.4 Hz, 1H), 4.79 (dd, J = 8.4, 5.3 Hz, 1H), 2.65 (dqd, J = 15.1, 10.1, 8.4 Hz, 1H), 2.52 (dqd, J = 15.4, 10.4, 5.3 Hz, 1H). ^{13}C NMR (126 MHz, $CDCl_3$) δ 138.4, 137.2, 136.9, 130.0, 128.9, 128.1, 127.8, 127.3, 127.2, 126.8, 125.4 (q, J = 277.4 Hz), 59.9 (q, J = 3.0 Hz), 40.5 (q, J = 28.3 Hz). ^{19}F NMR (377 MHz, $CDCl_3$) δ -63.99 (t, J = 10.2 Hz). HRMS (EI): $[M]^+$: Calculated: 317.1140; Found: 317.1144.

NMR Spectra of Compounds.

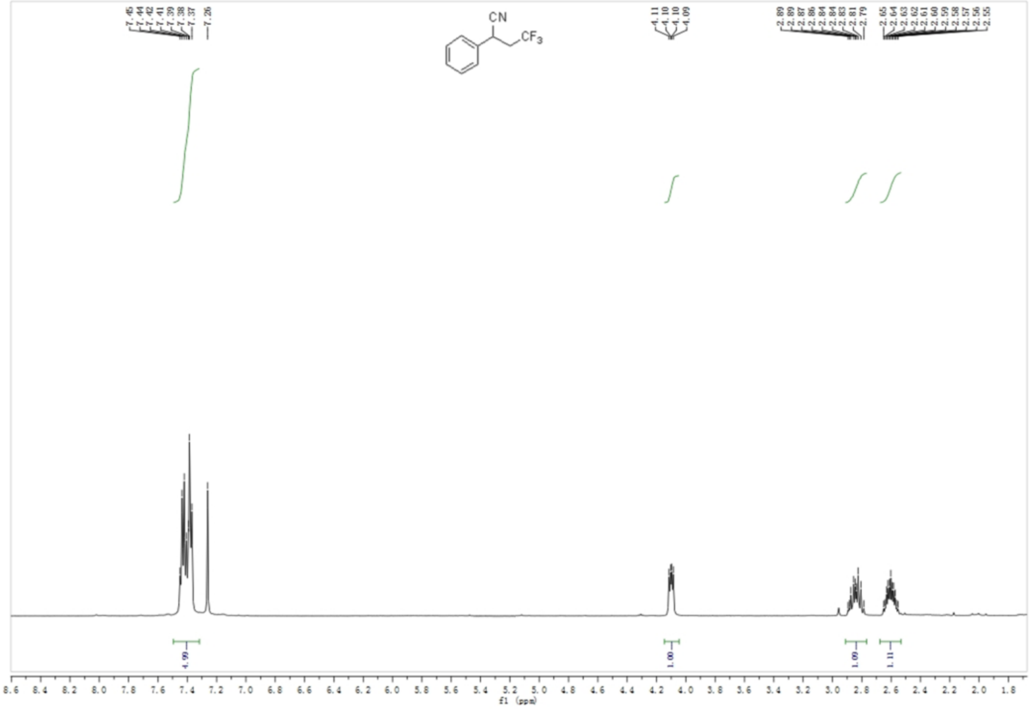

Figure S2 2a-^{1}H NMR

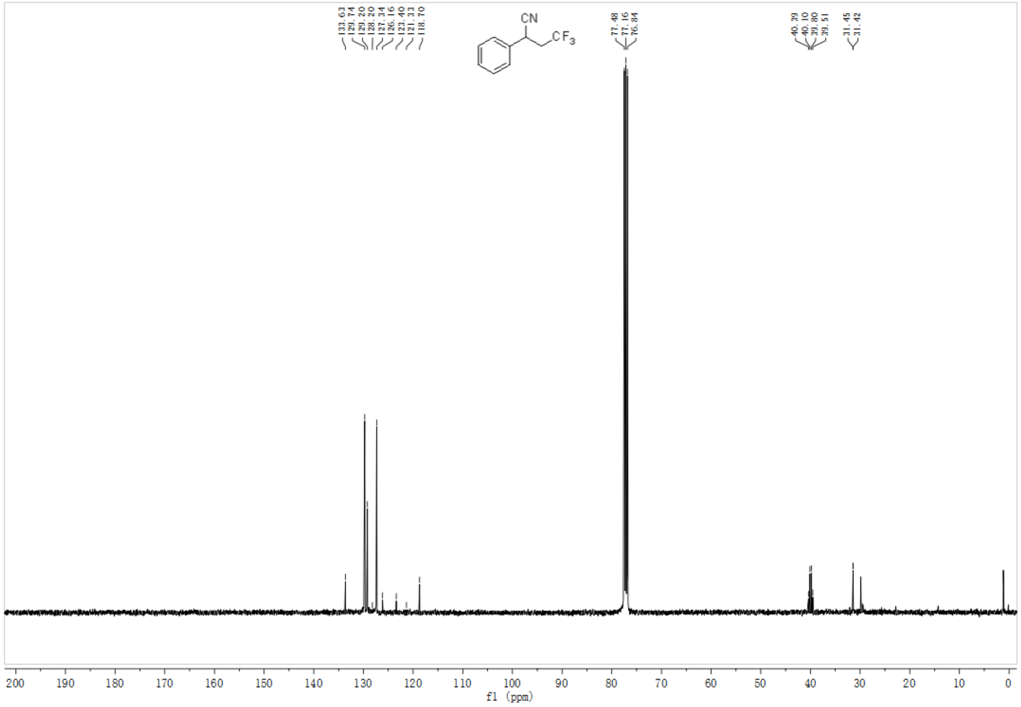

Figure S3 2a-^{13}C NMR

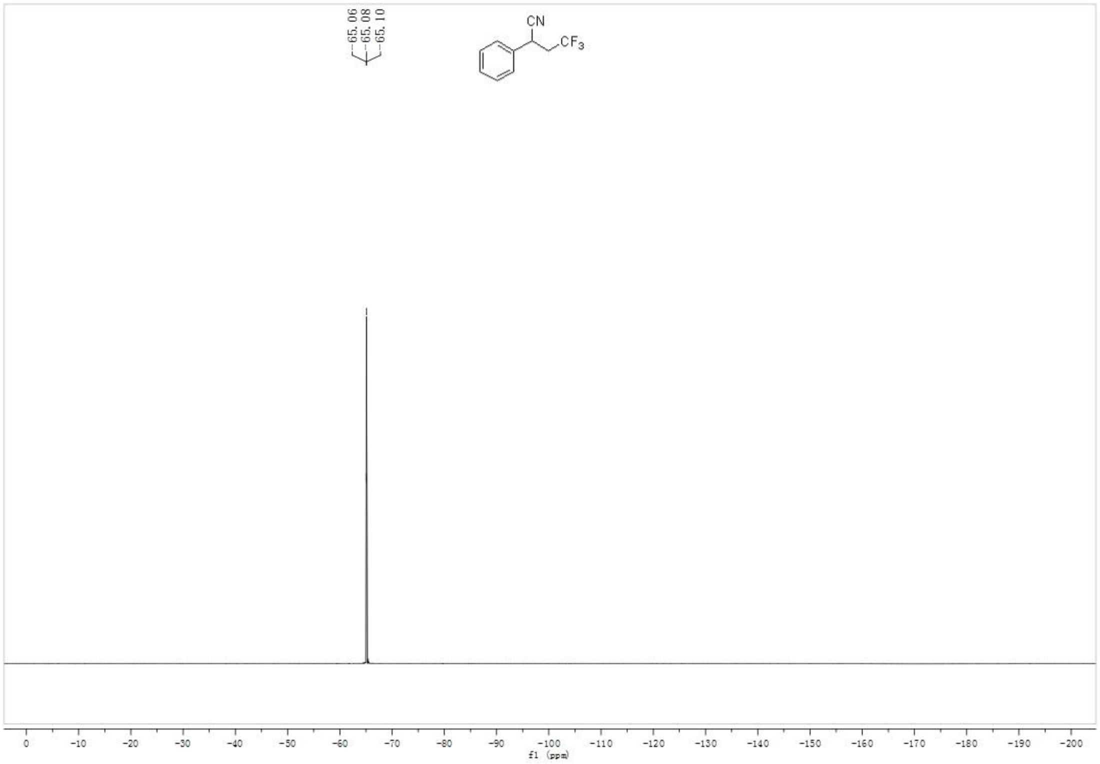

Figure S4 2a-^{19}F NMR

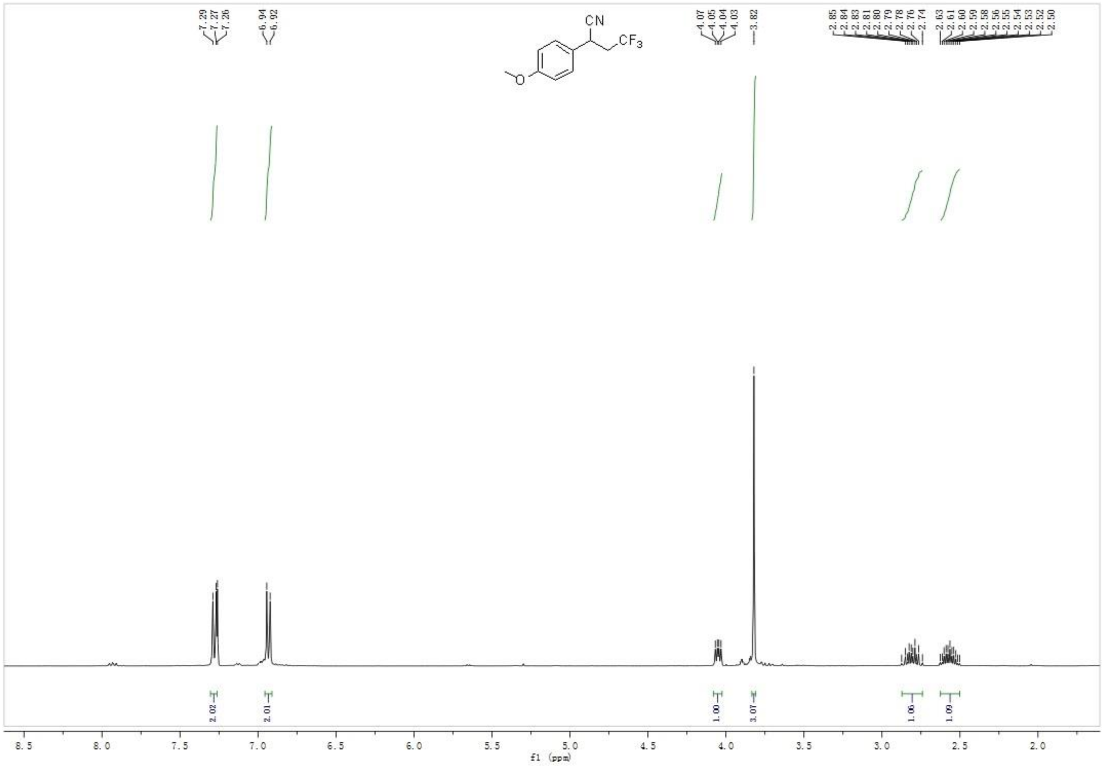

Figure S5 2b-^{1}H NMR

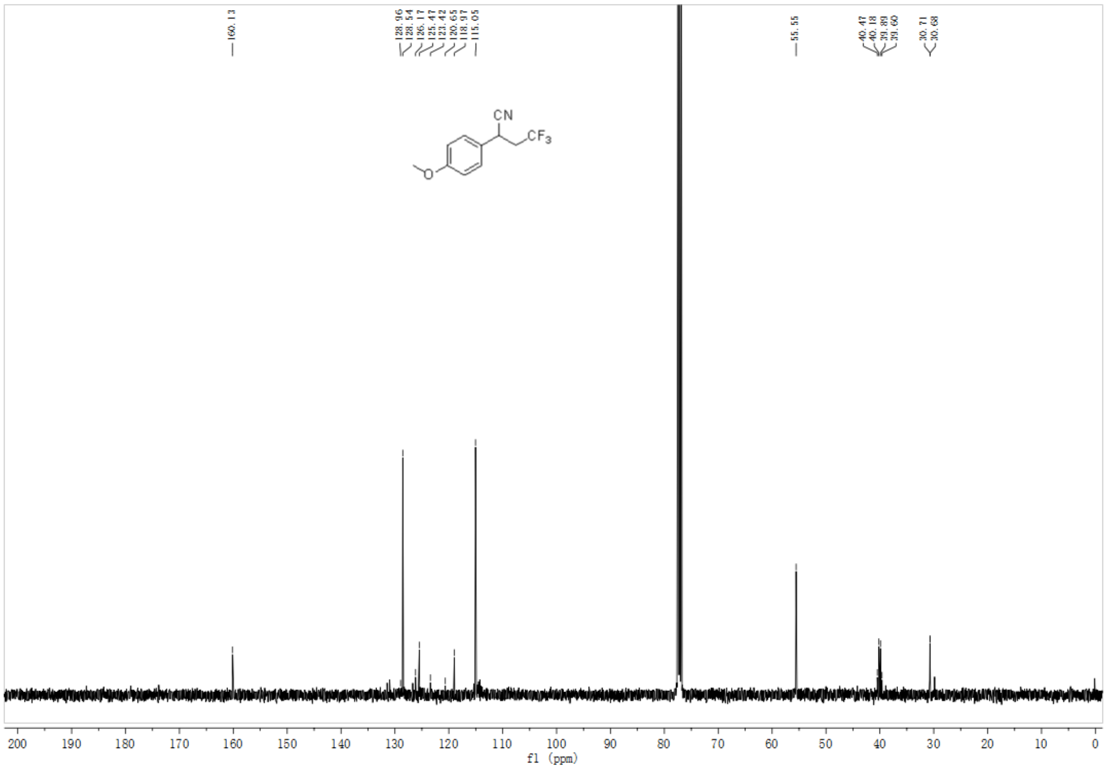

Figure S6 2b-^{13}C NMR

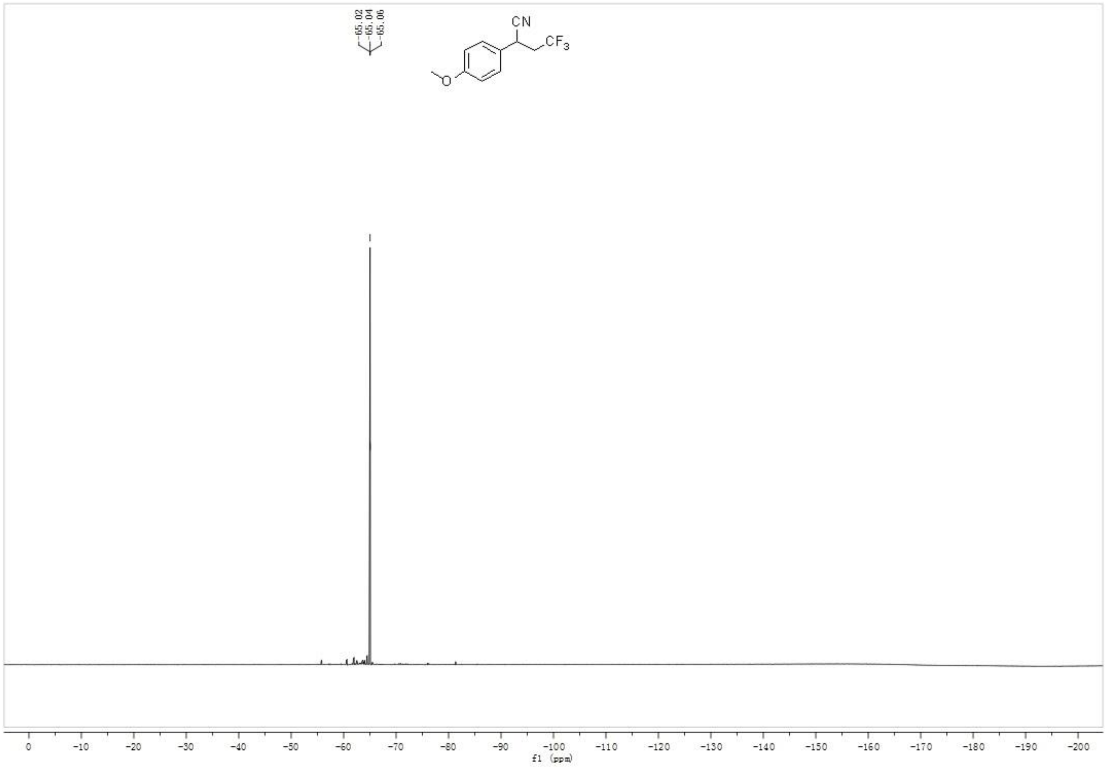

Figure S7 2b-^{19}F NMR

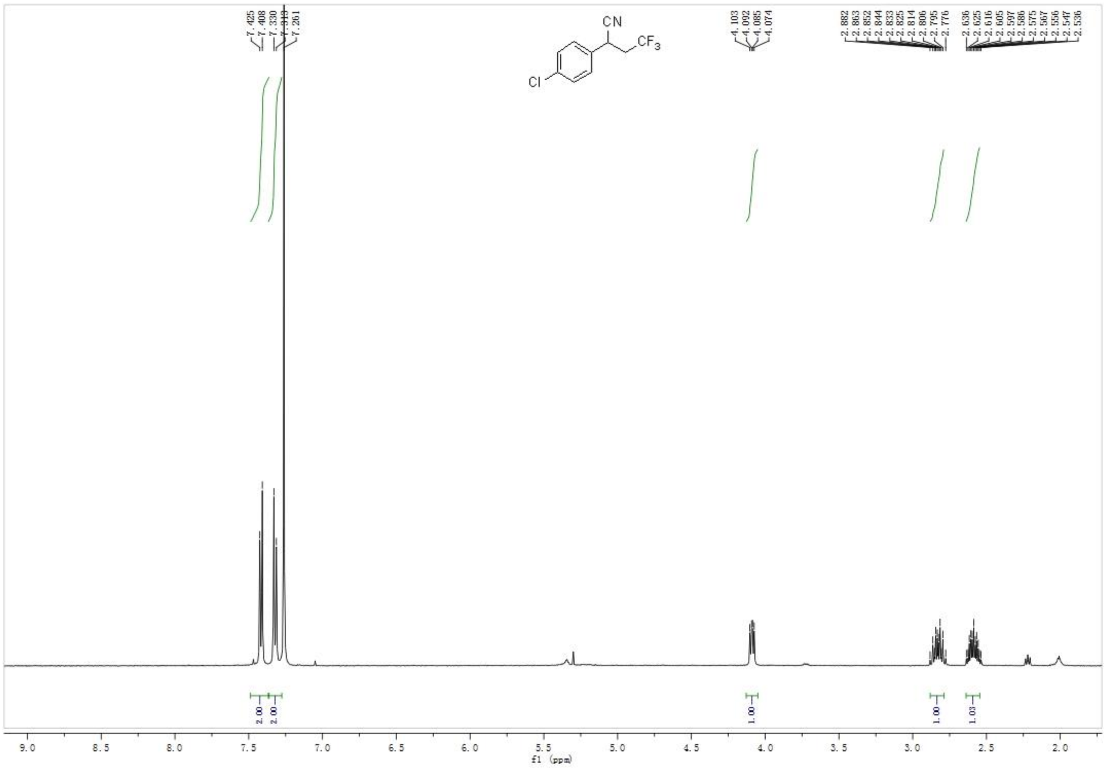

Figure S8 2c-^{1}H NMR

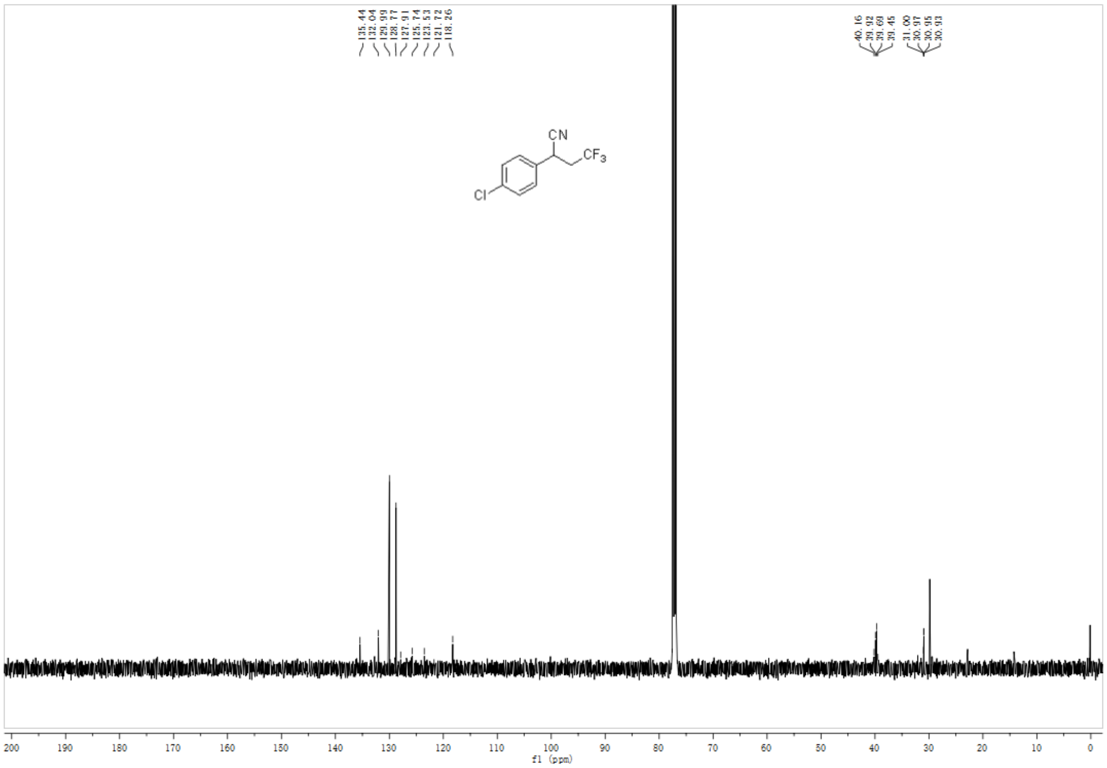

Figure S9 2c-^{13}C NMR

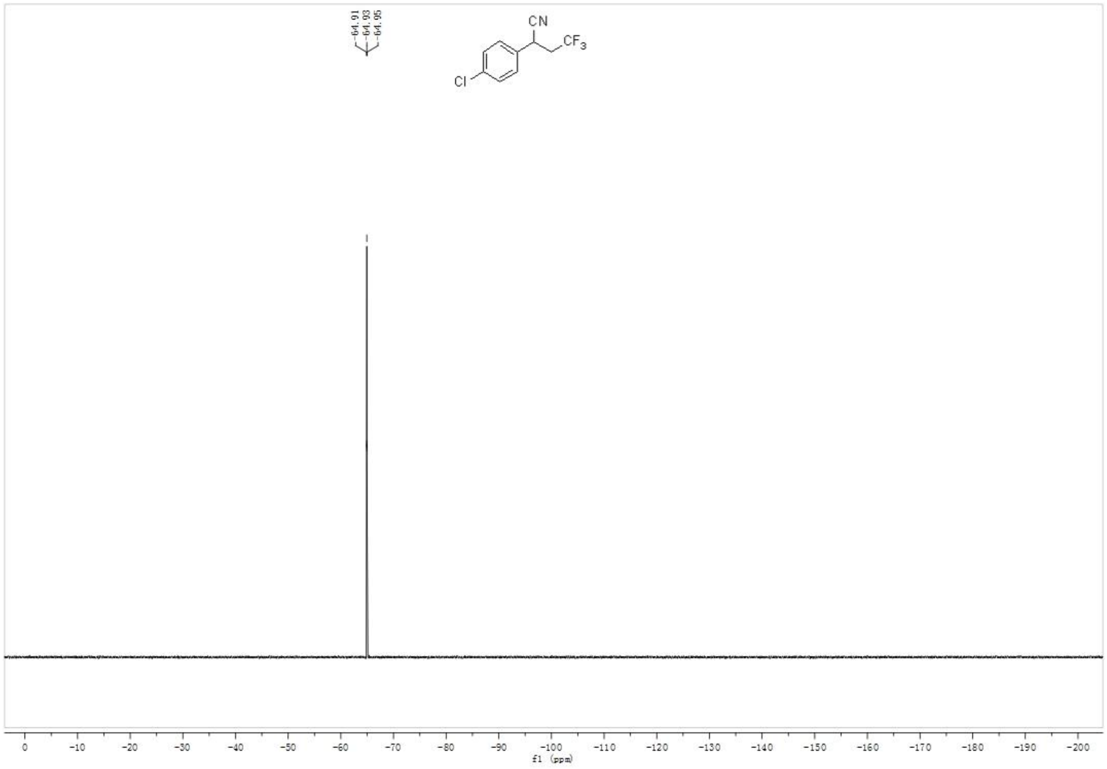

Figure S10 2c-^{19}F NMR

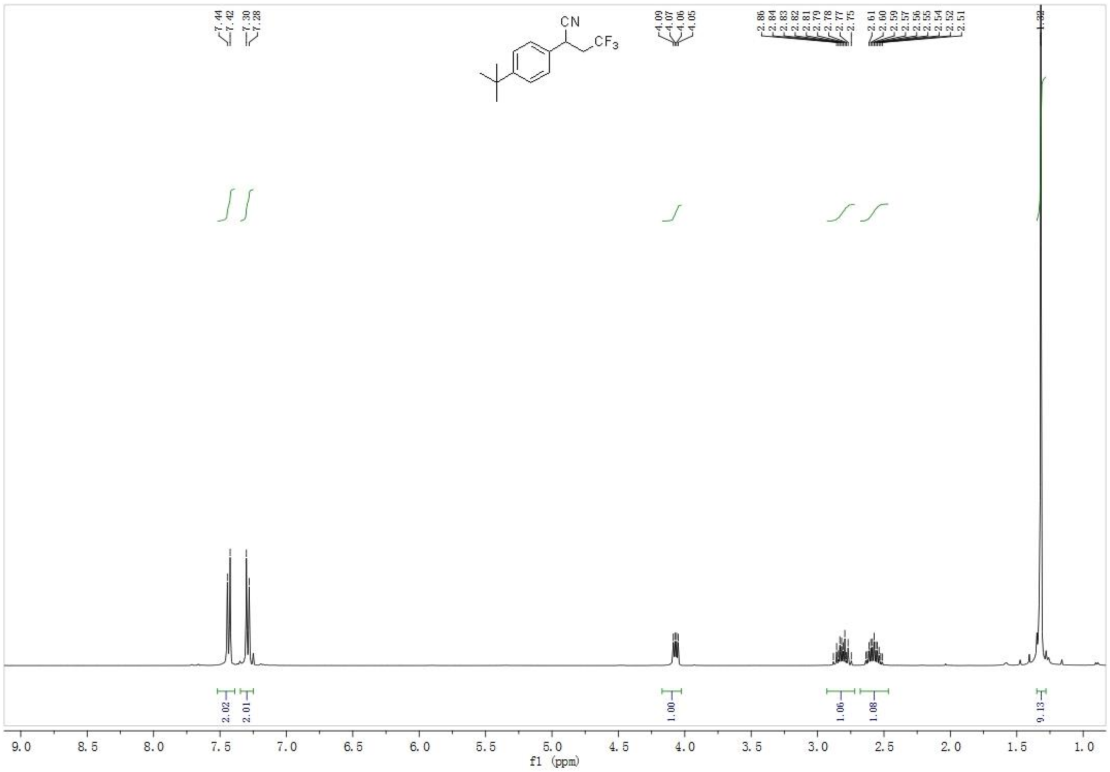

Figure S11 2d-^{1}H NMR

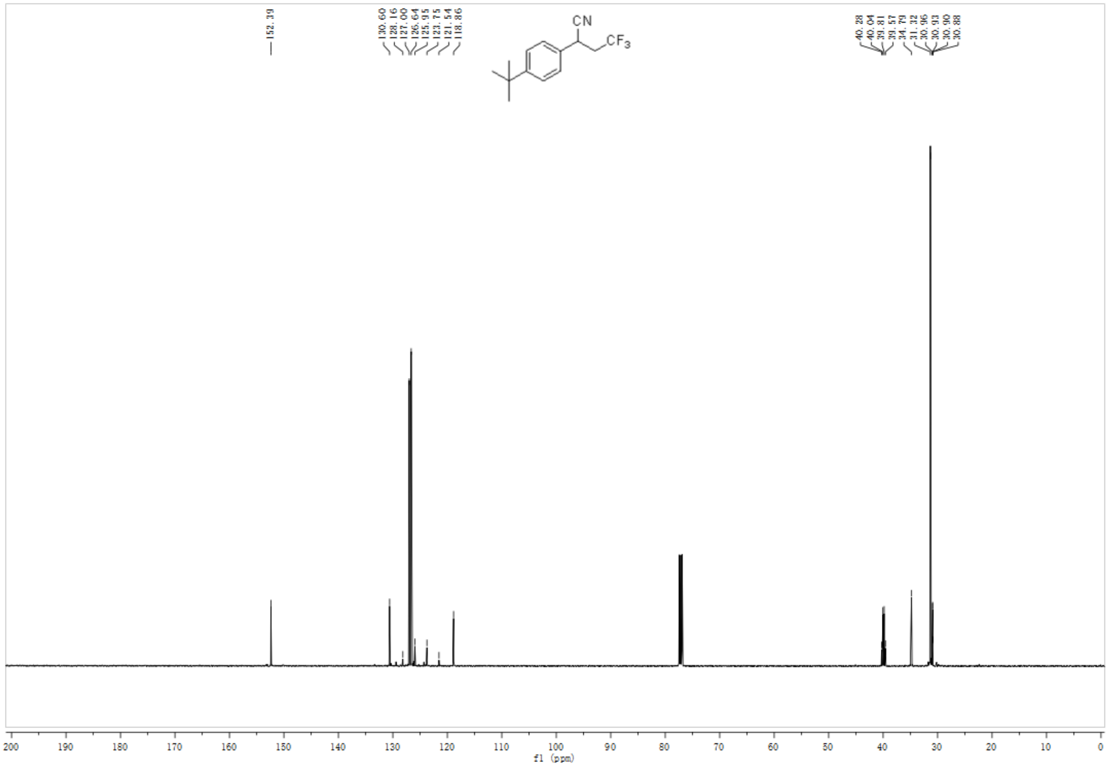

Figure S12 2d-^{13}C NMR

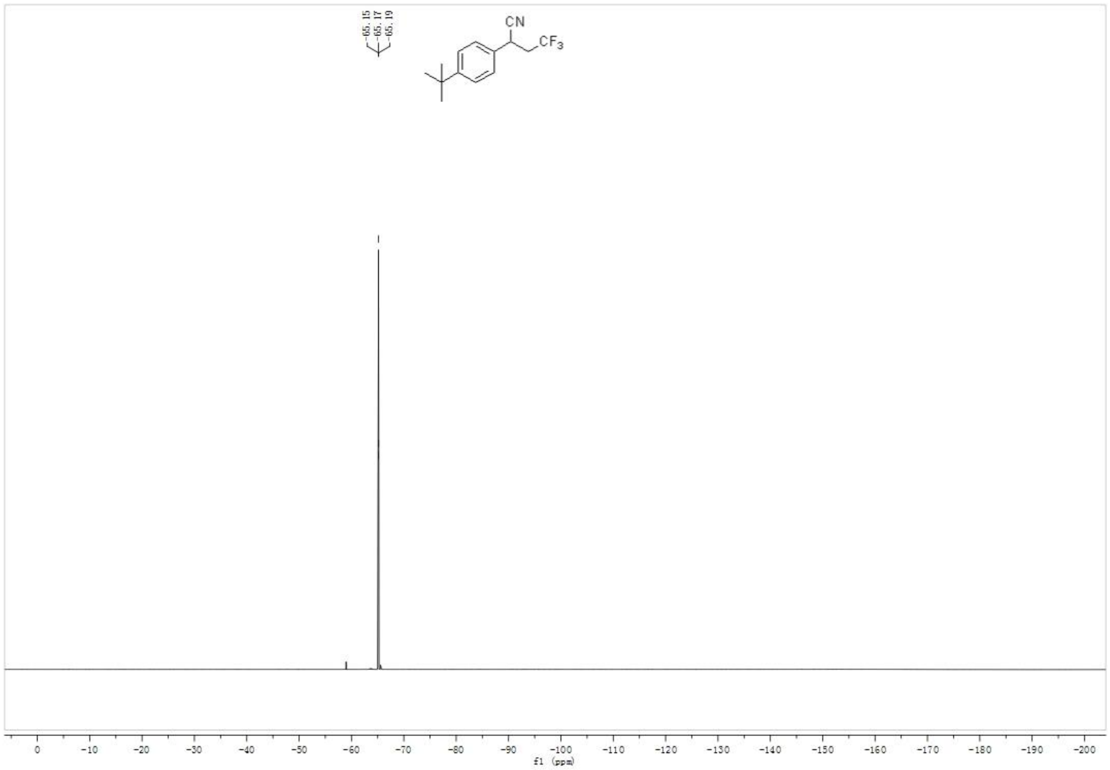

Figure S13 2d-^{13}F NMR

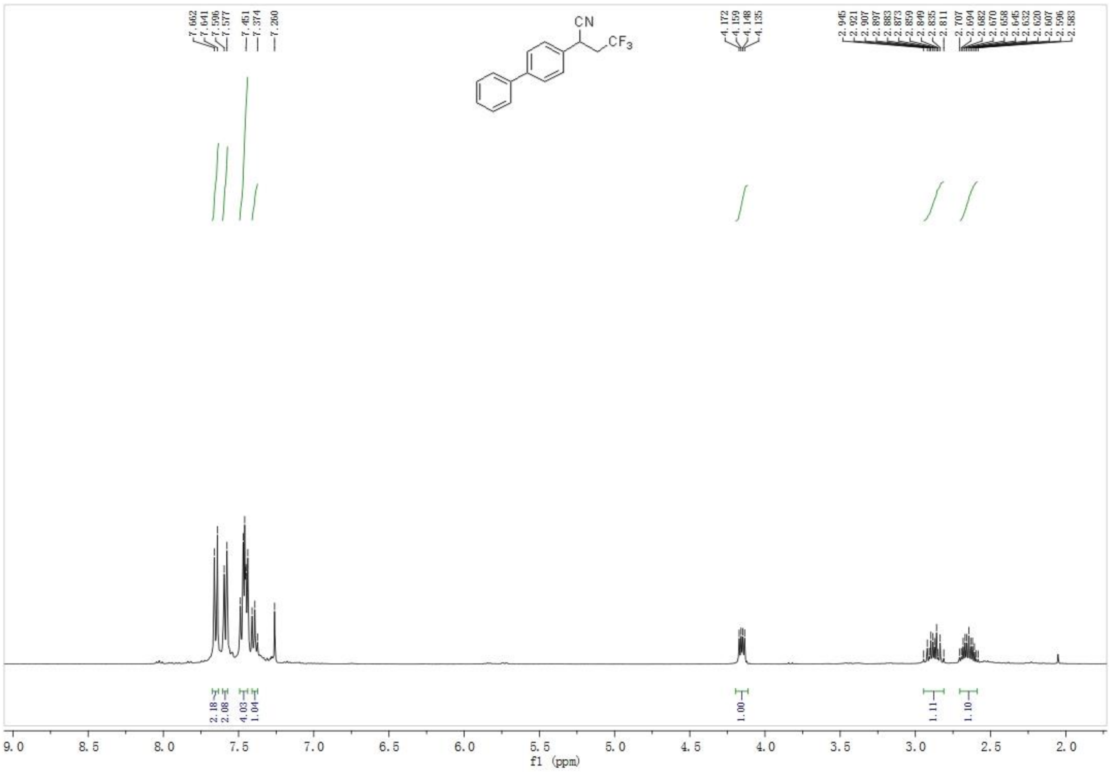

Figure S14 2e-^{1}H NMR

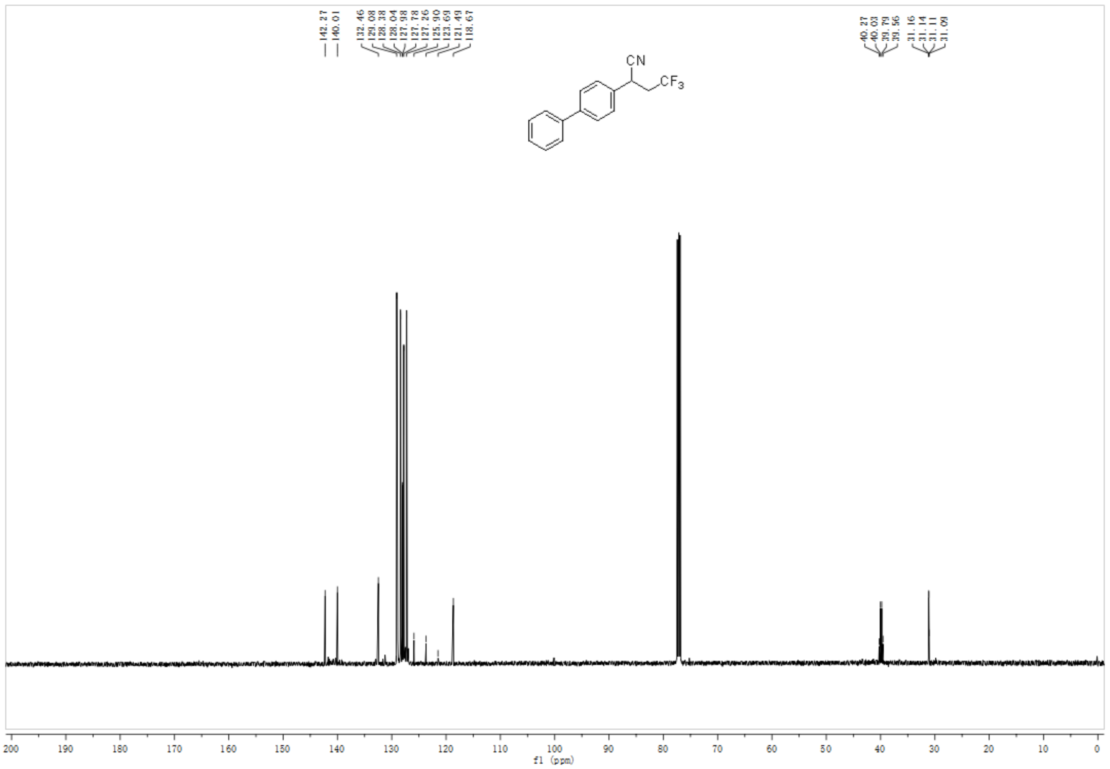

Figure S15 2e-^{13}C NMR

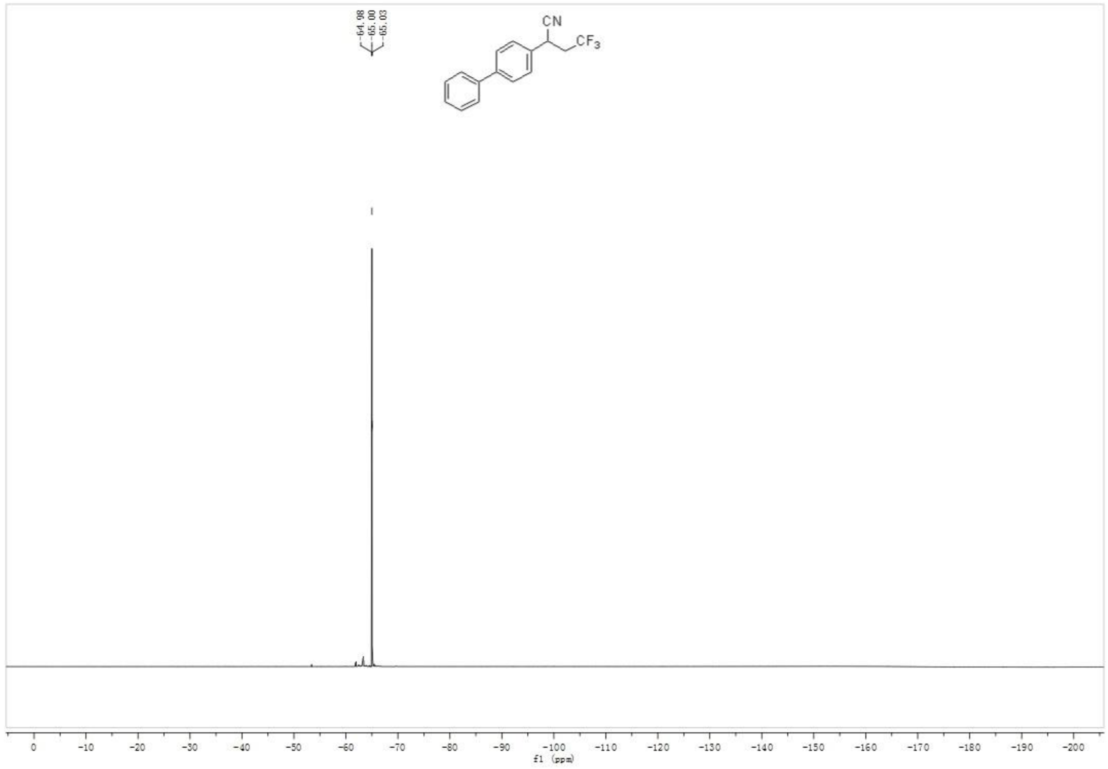

Figure S16 2e-^{19}F NMR

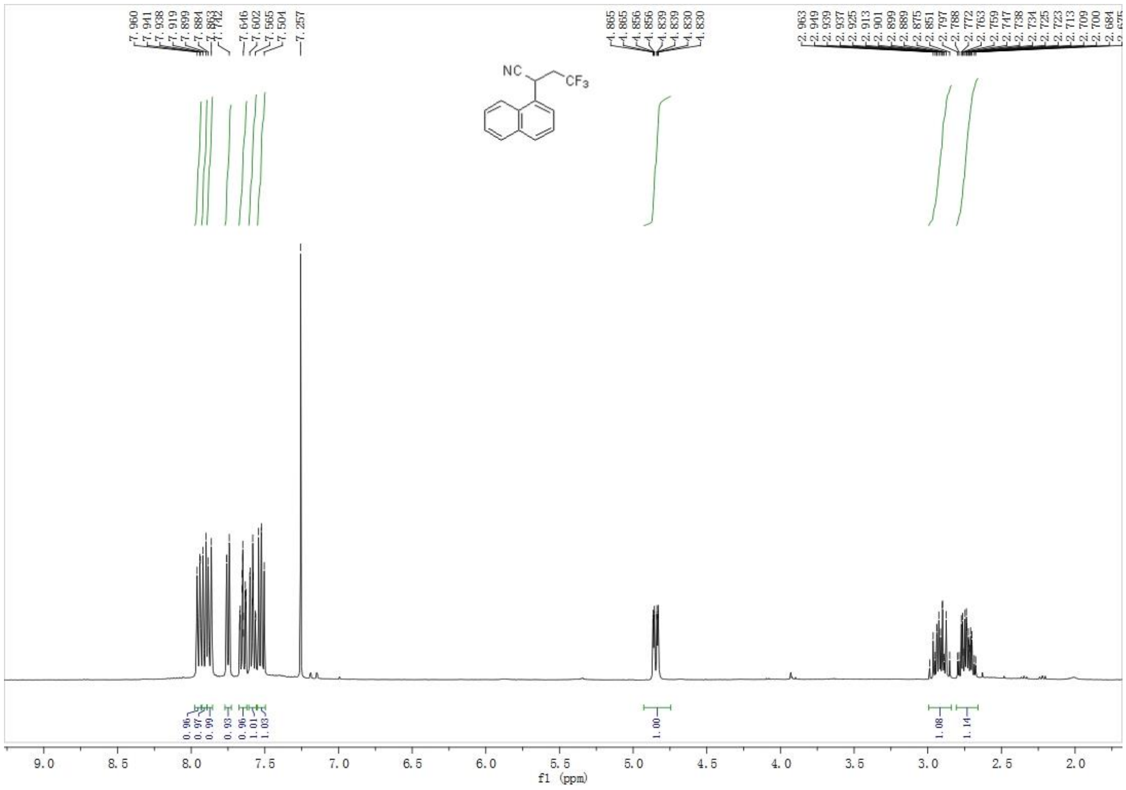

Figure S17 2f-^{1}H NMR

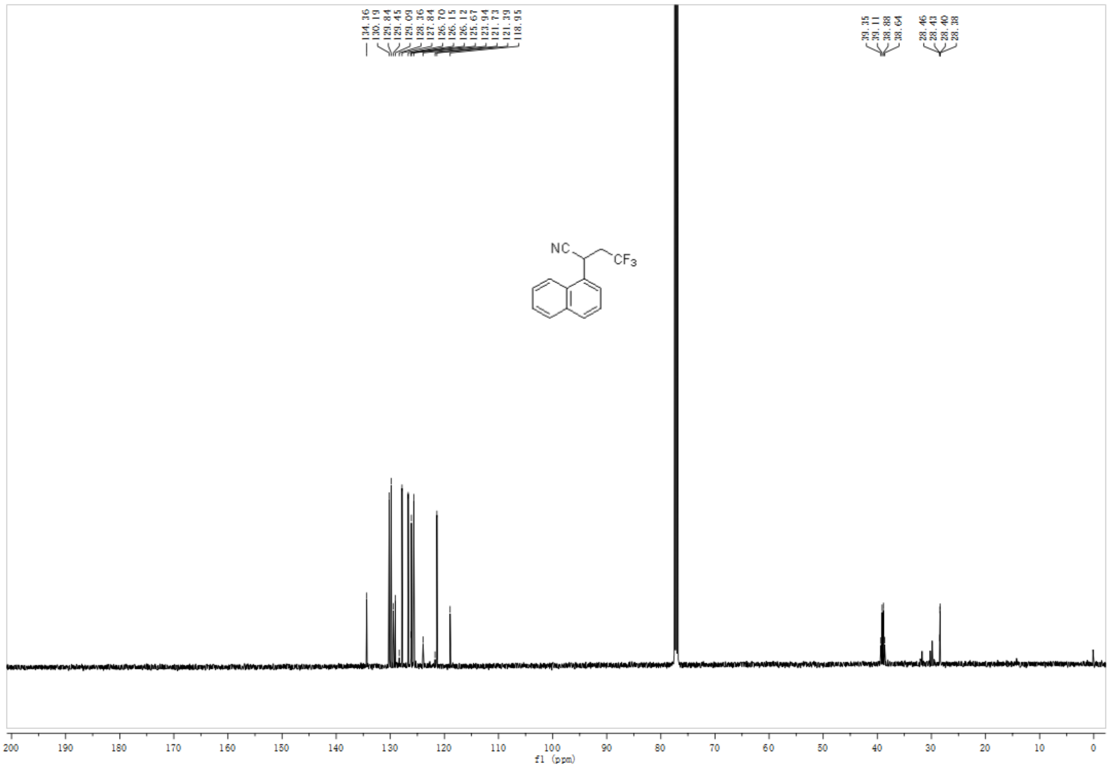

Figure S18 2f-^{13}C NMR

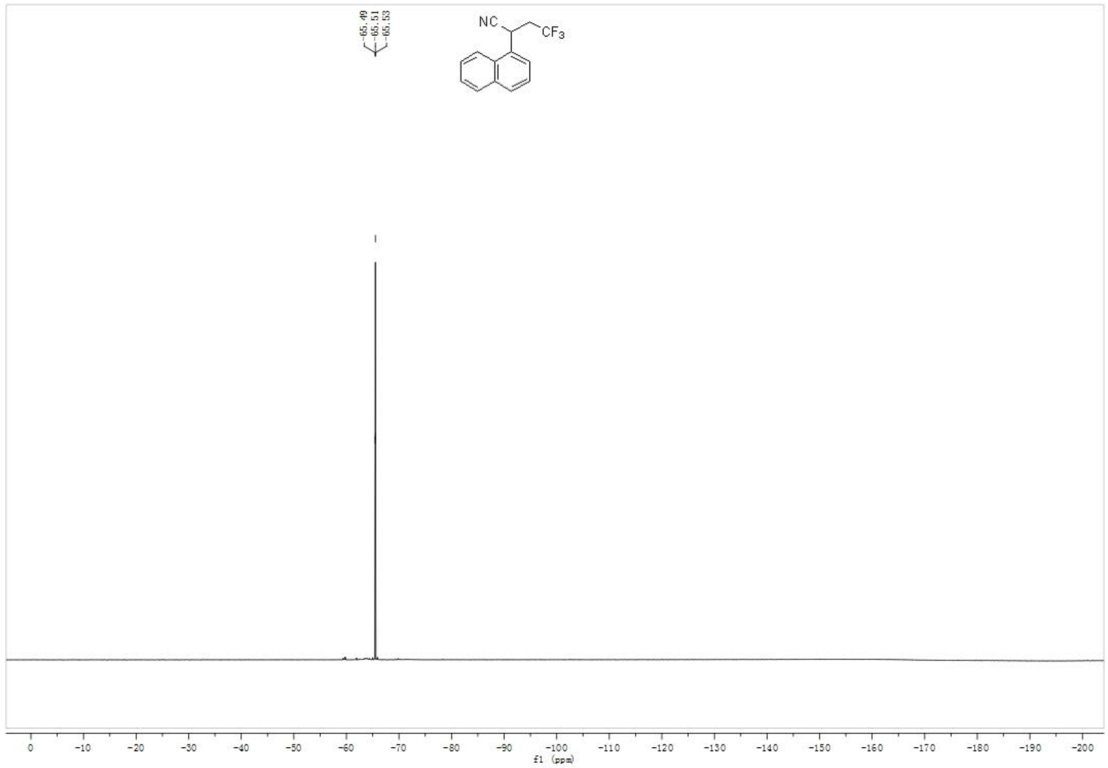

Figure S19 2f-^{19}F NMR

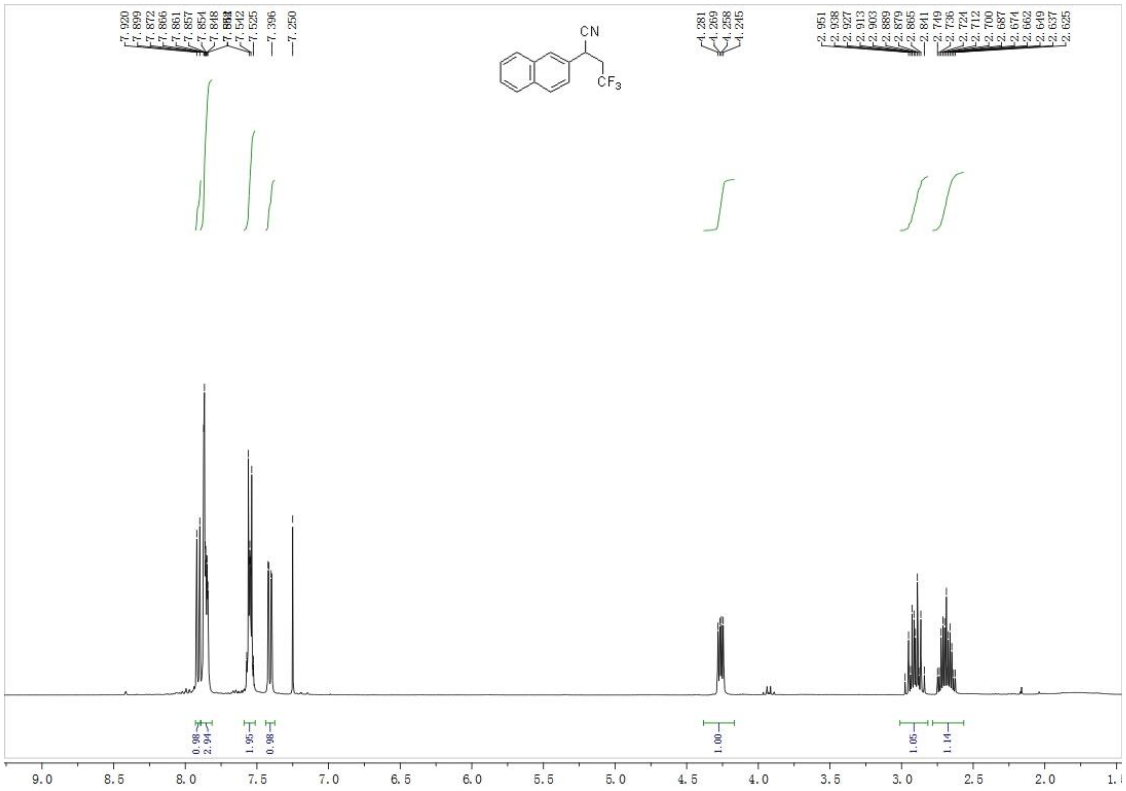

Figure S20 2g-^{1}H NMR

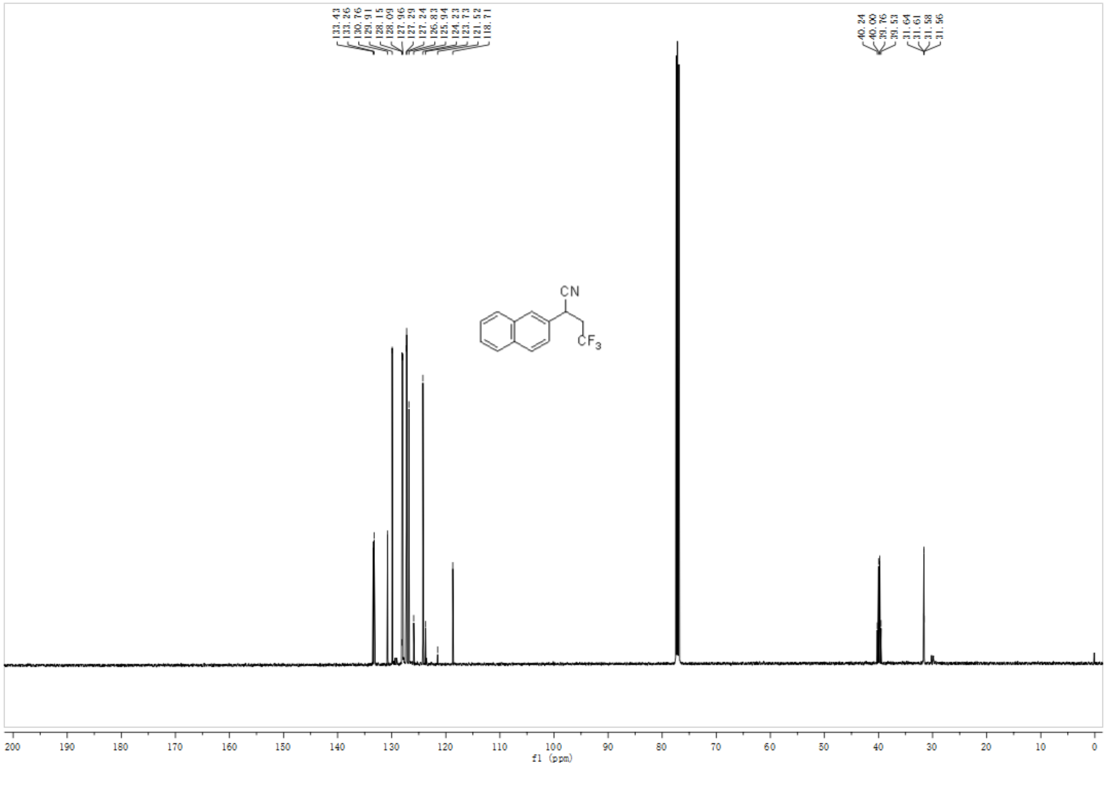

Figure S21 2g-^{13}C NMR

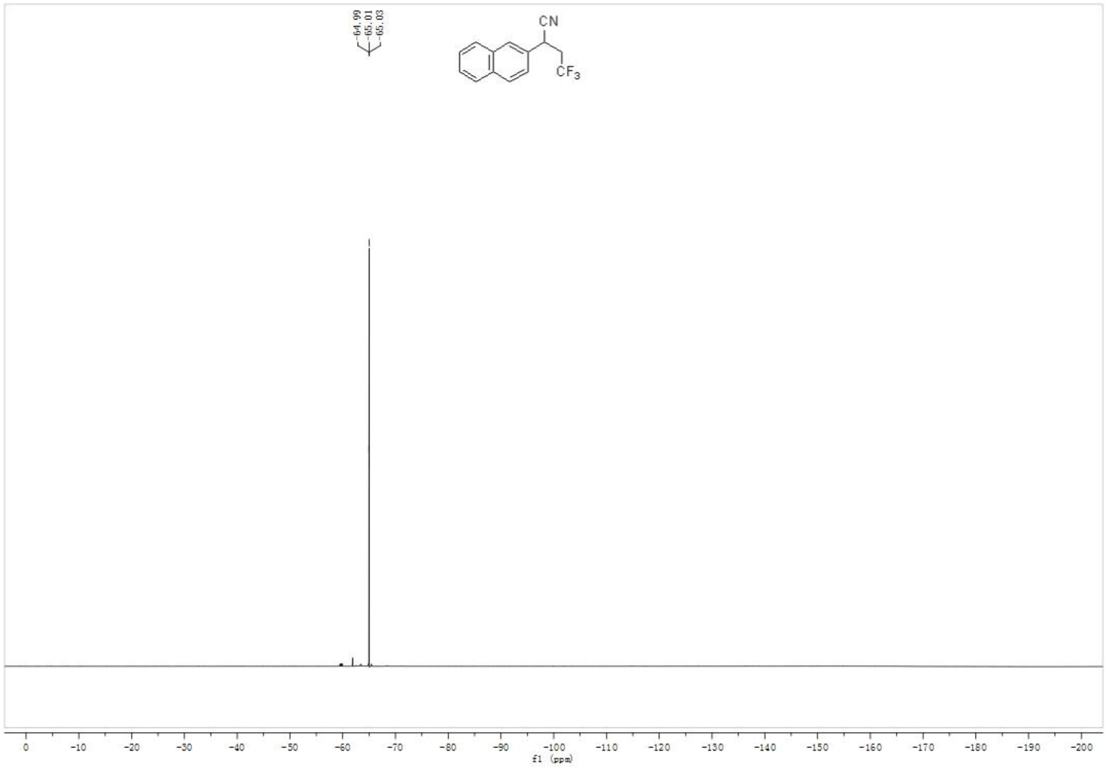

Figure S22 2g-^{19}F NMR

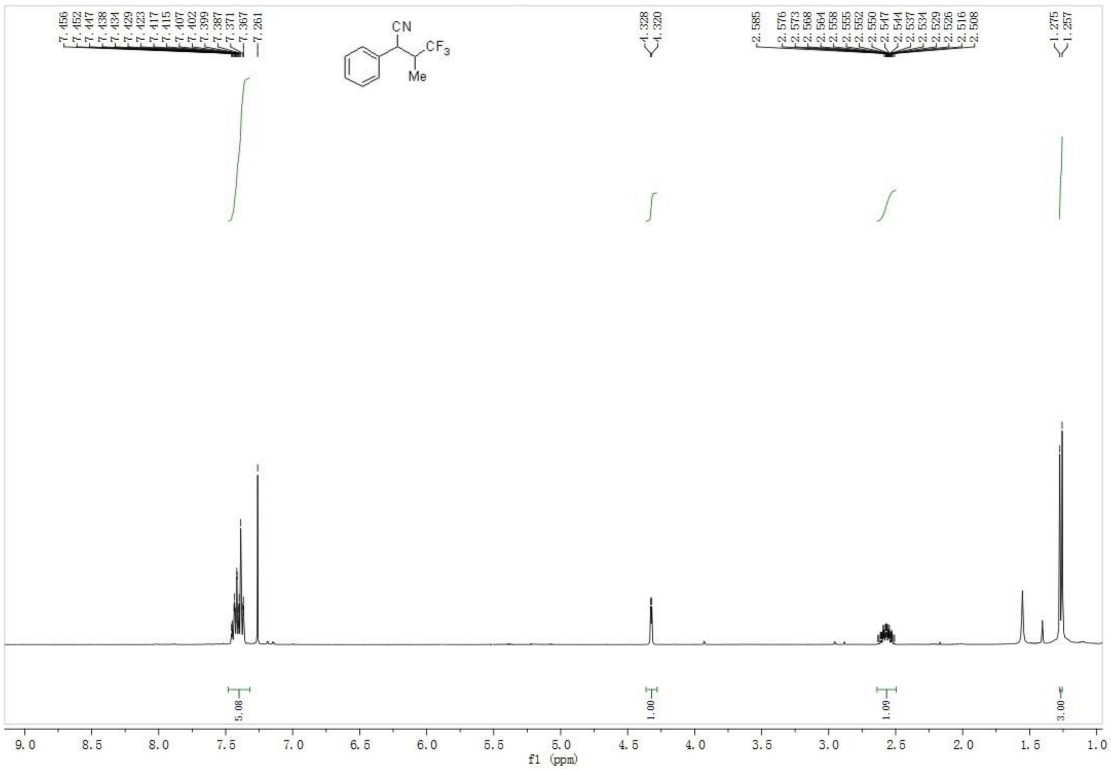

Figure S23 2h-^{1}H NMR

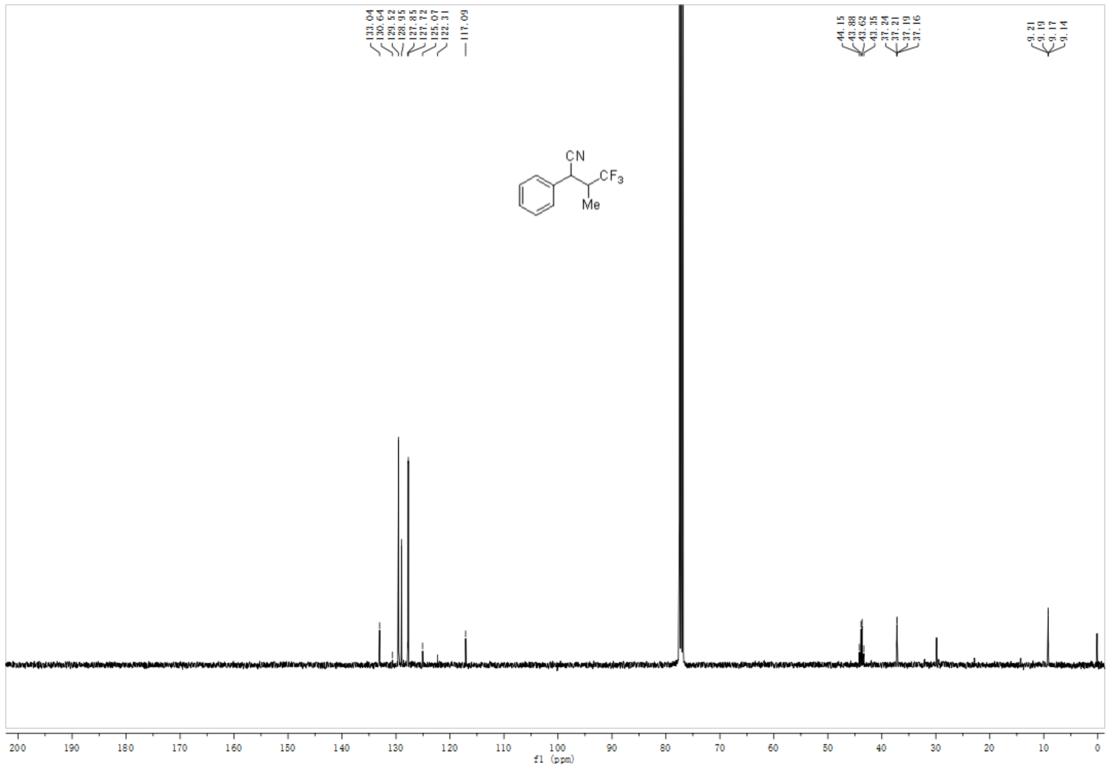

Figure S24 2h-^{13}C NMR

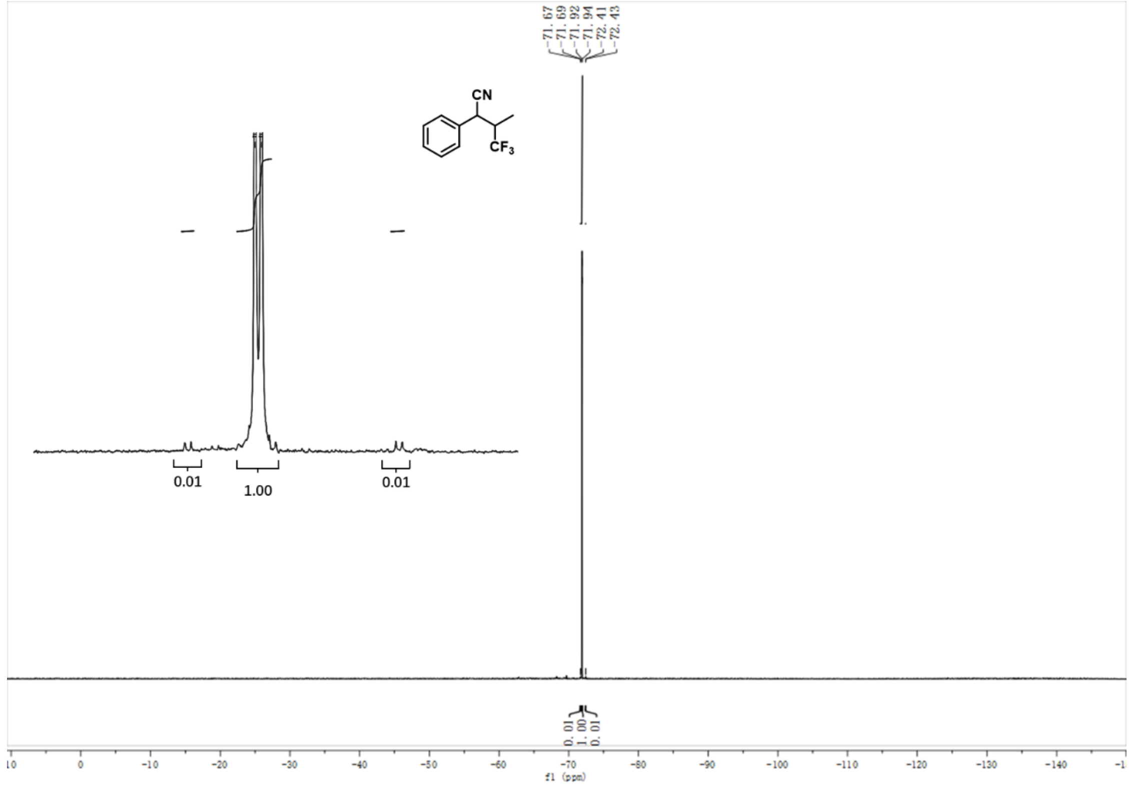

Figure S25 2h-^{19}F NMR

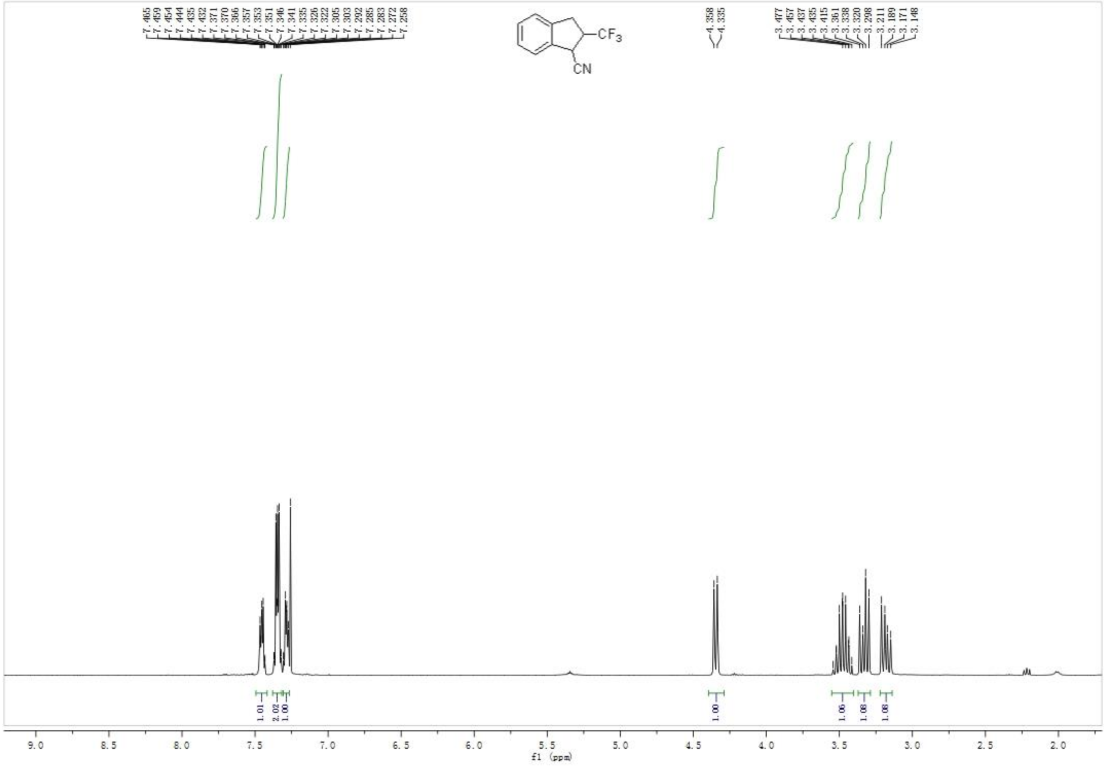

Figure S26 2i-^{1}H NMR

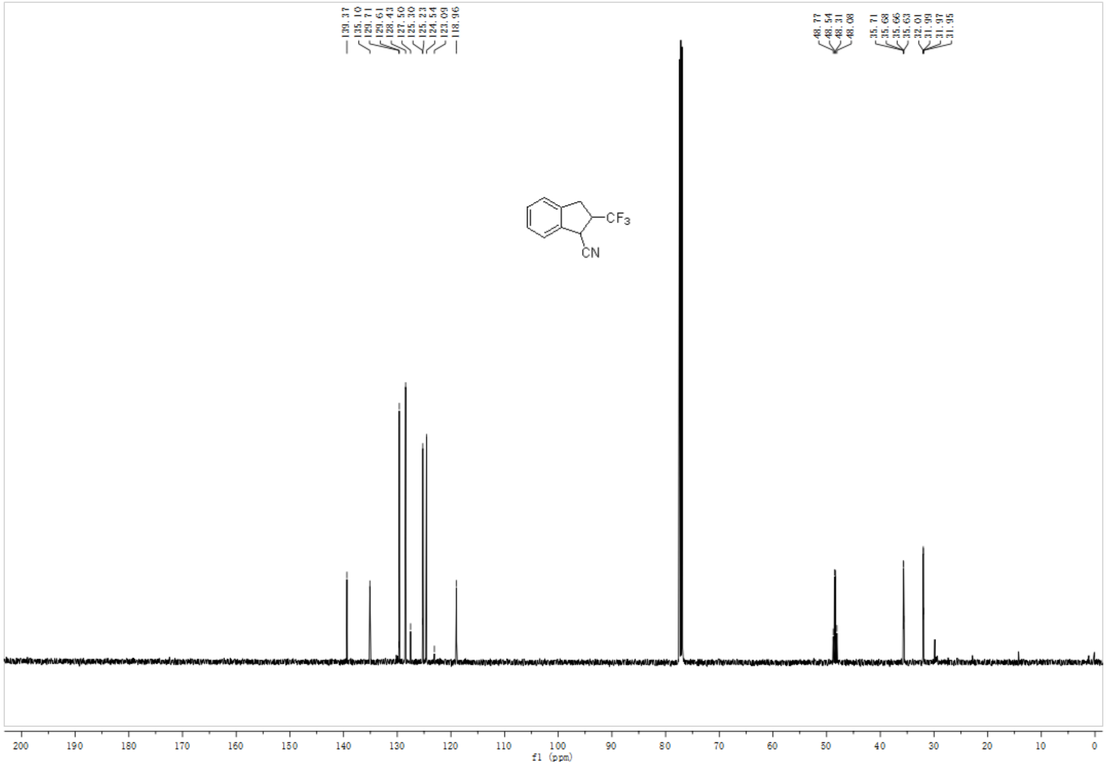

Figure S27 2i-^{13}C NMR

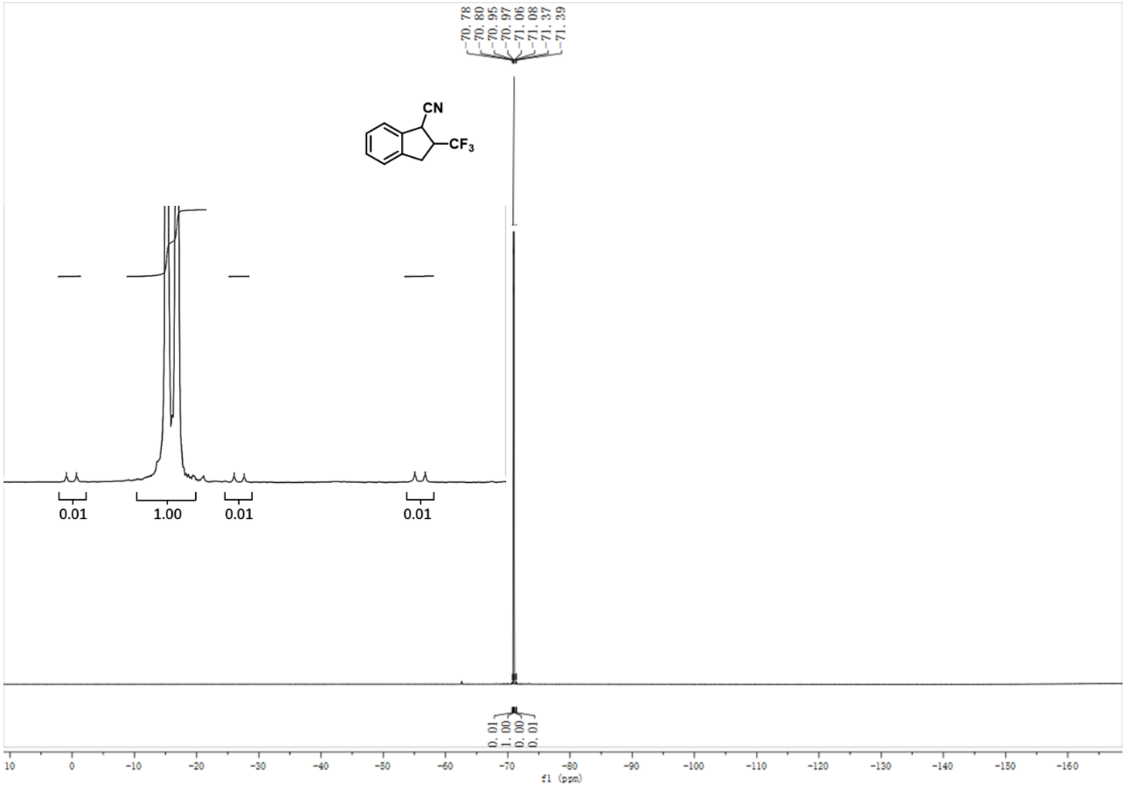

Figure S28 2i-^{19}F NMR

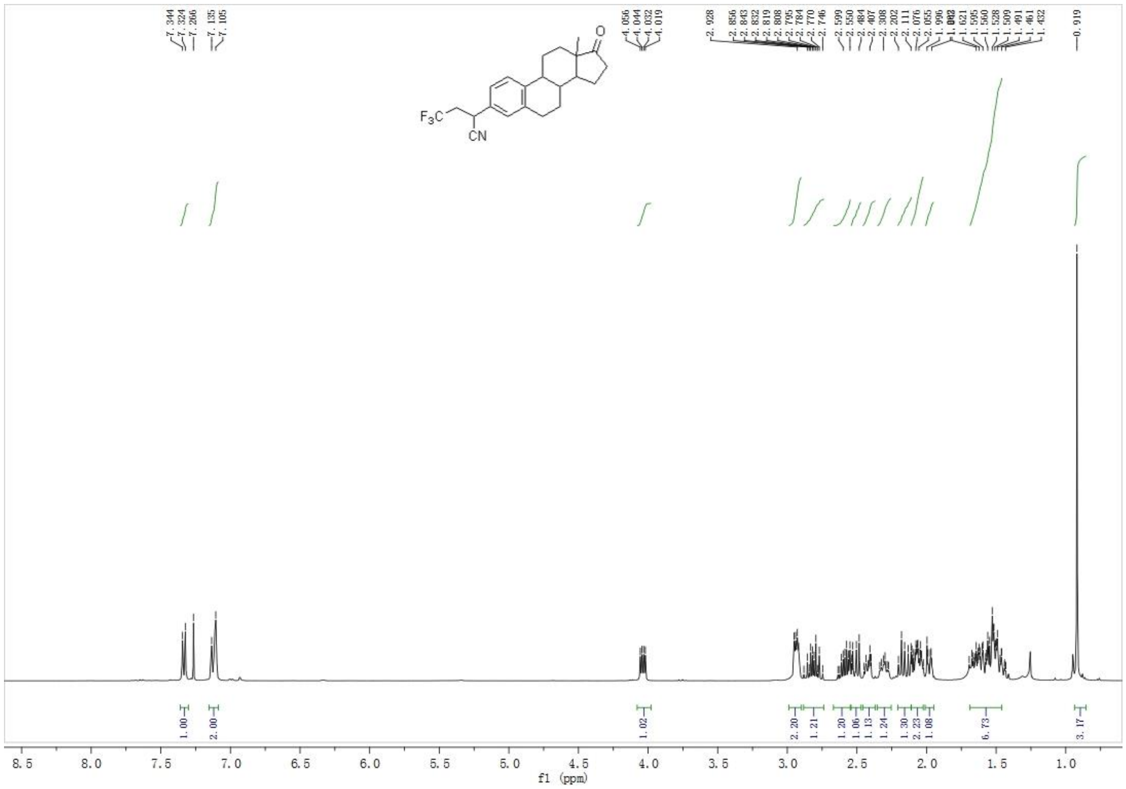

Figure S29 2k-^{1}H NMR

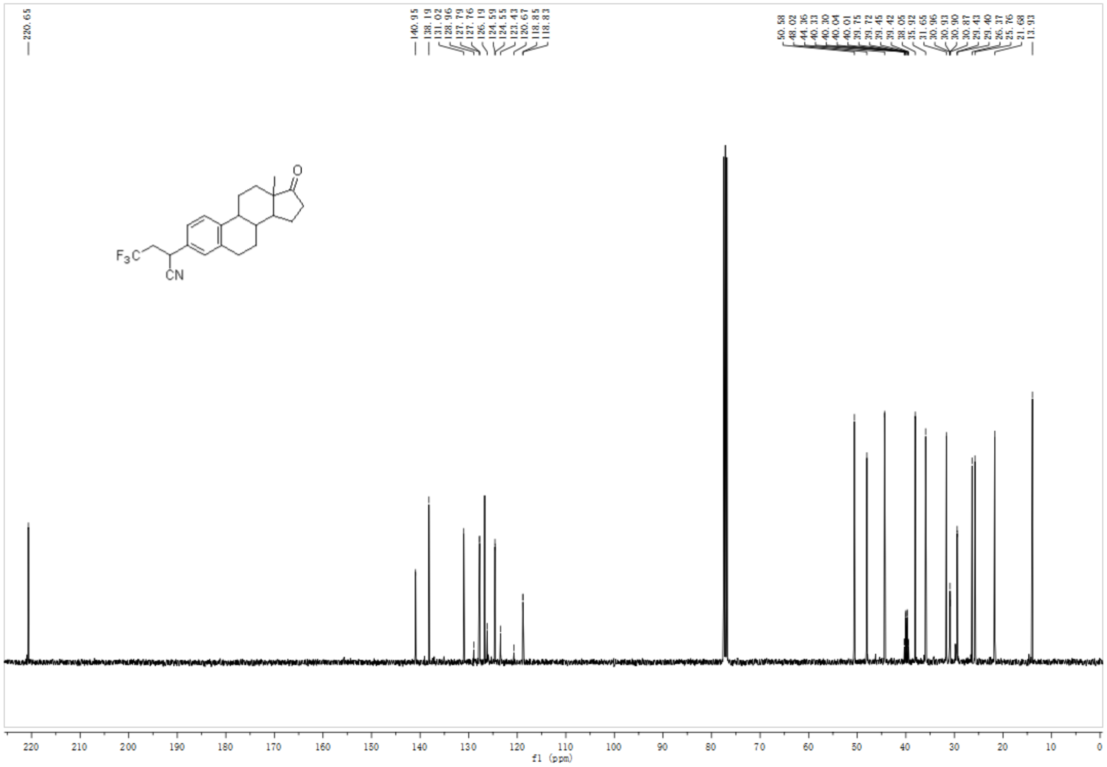

Figure S30 2k-^{13}C NMR

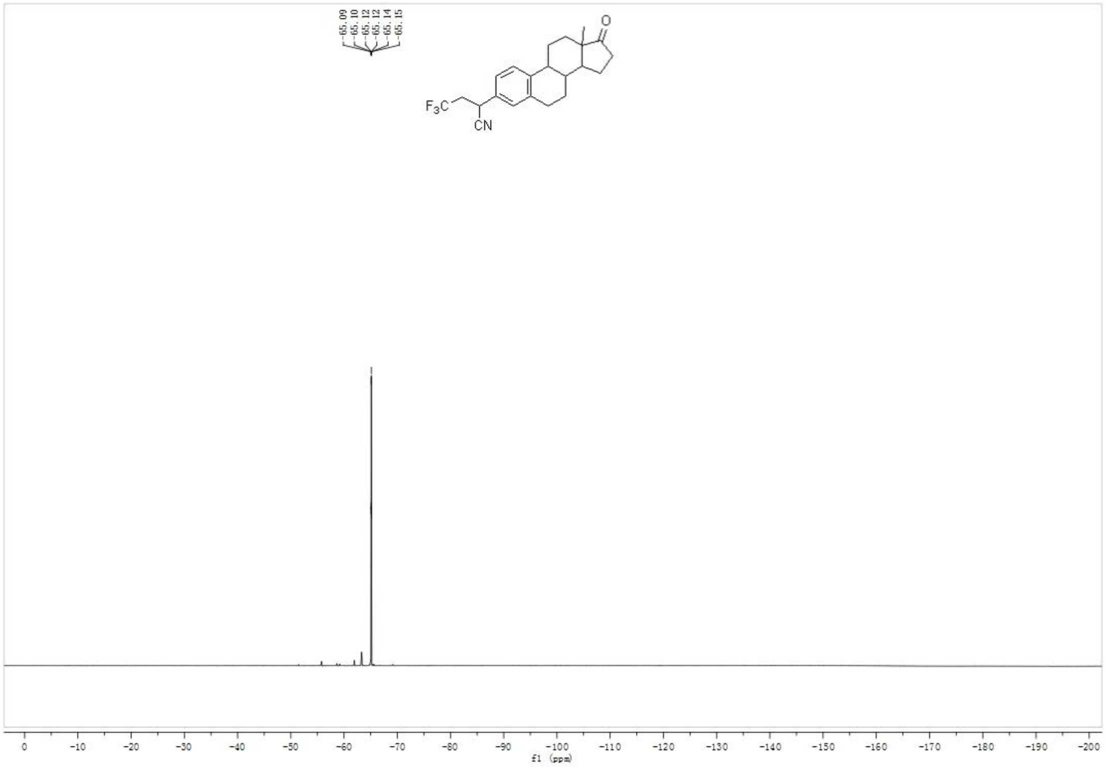

Figure S31 2k-^{19}F NMR

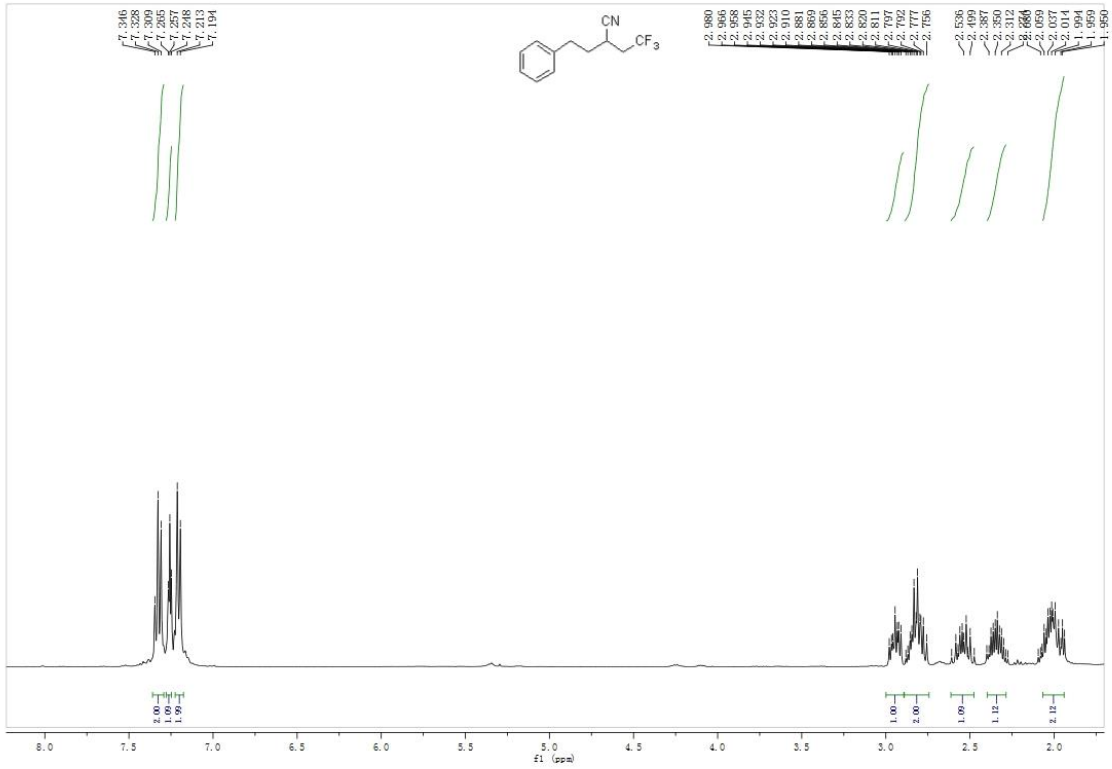

Figure S32 2l-^{1}H NMR

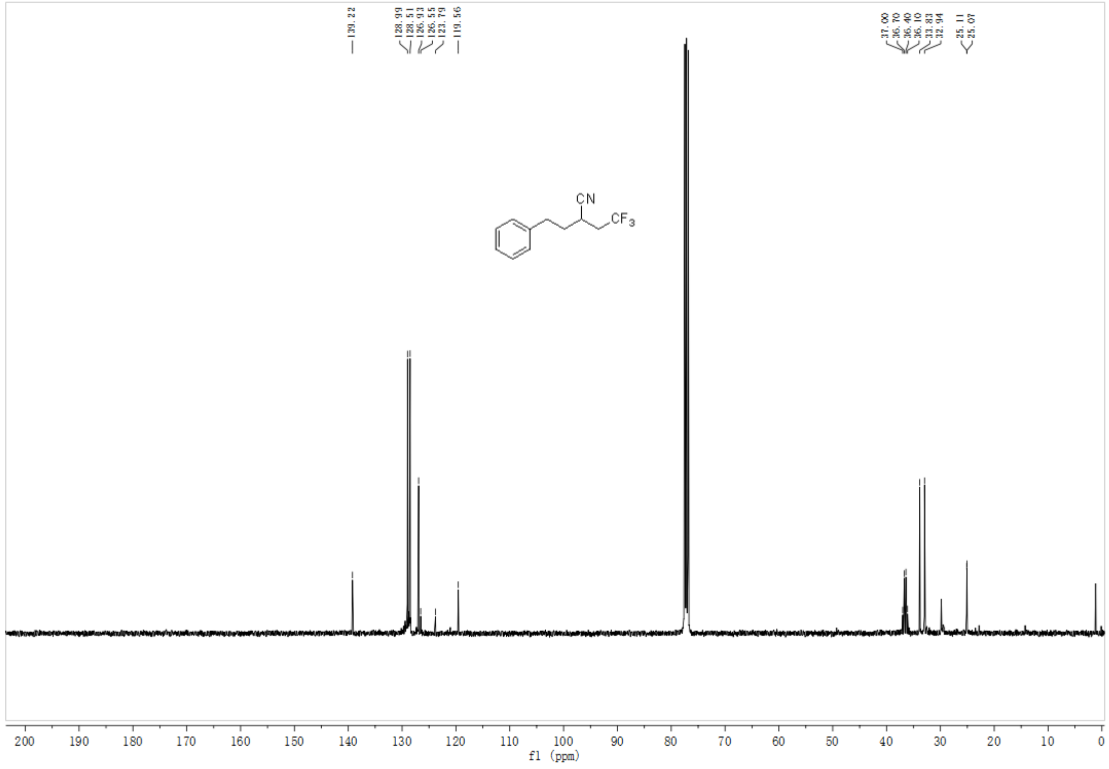

Figure S33 2l-^{13}C NMR

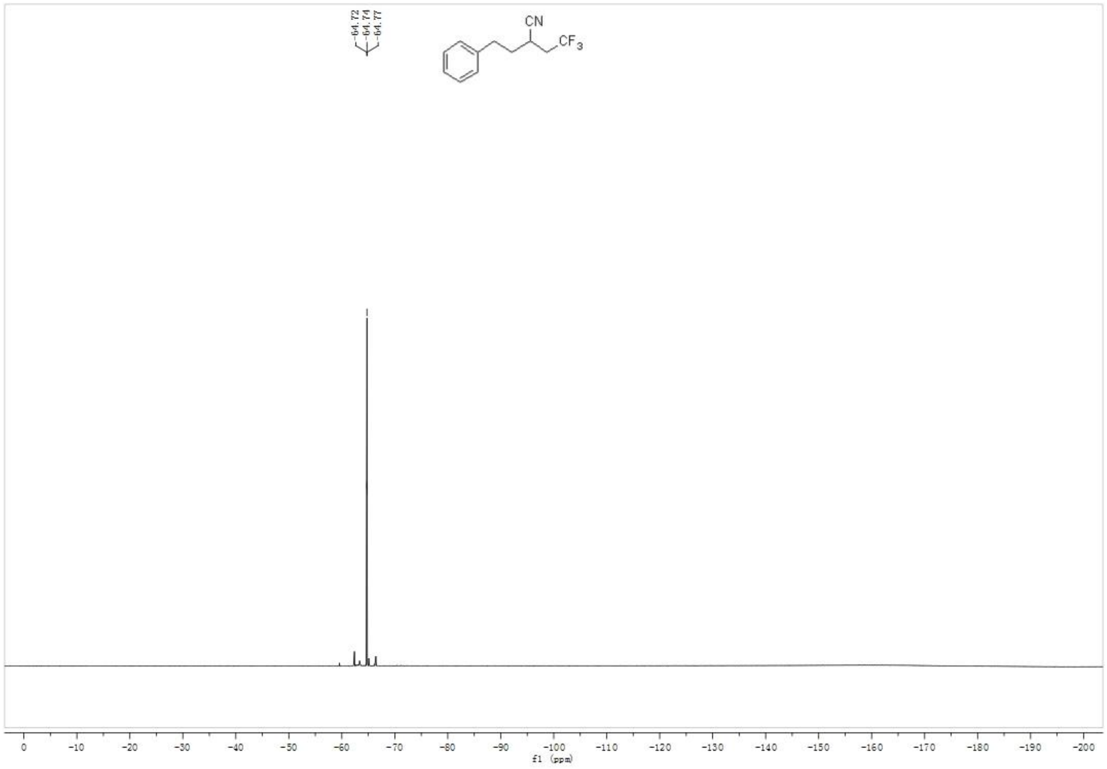

Figure S34 2l-^{19}F NMR

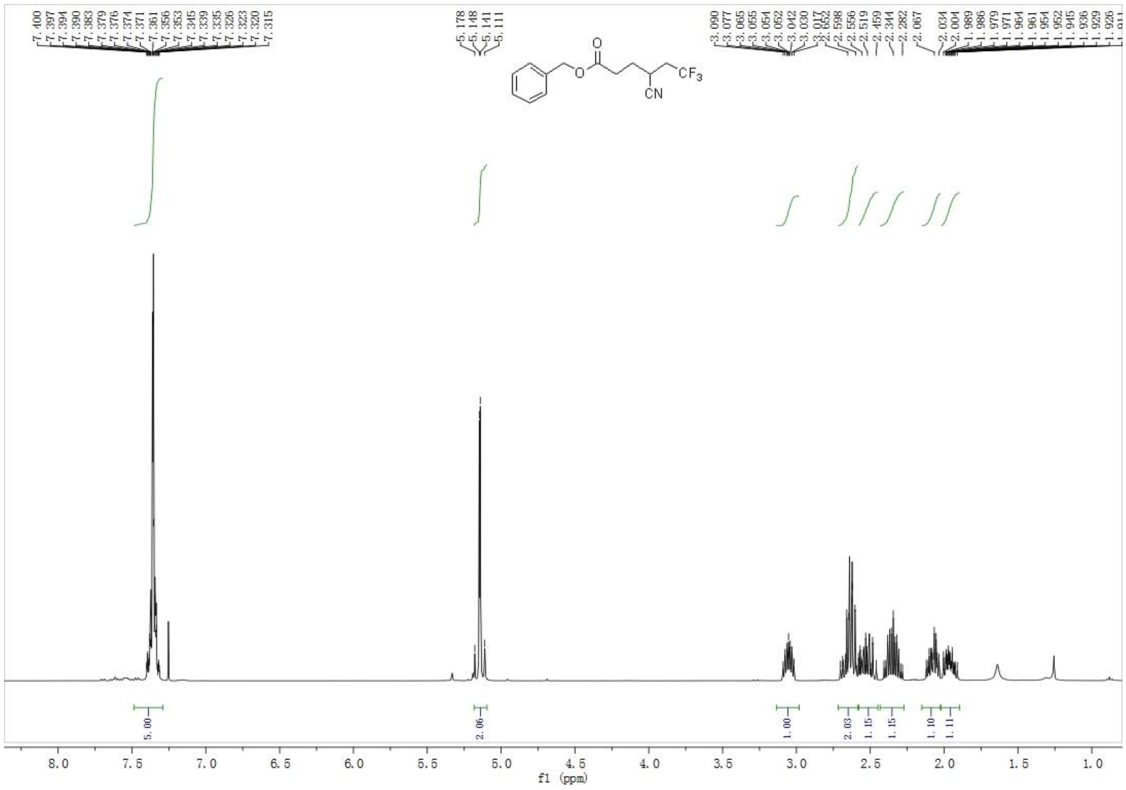

Figure S35 2m-^{1}H NMR

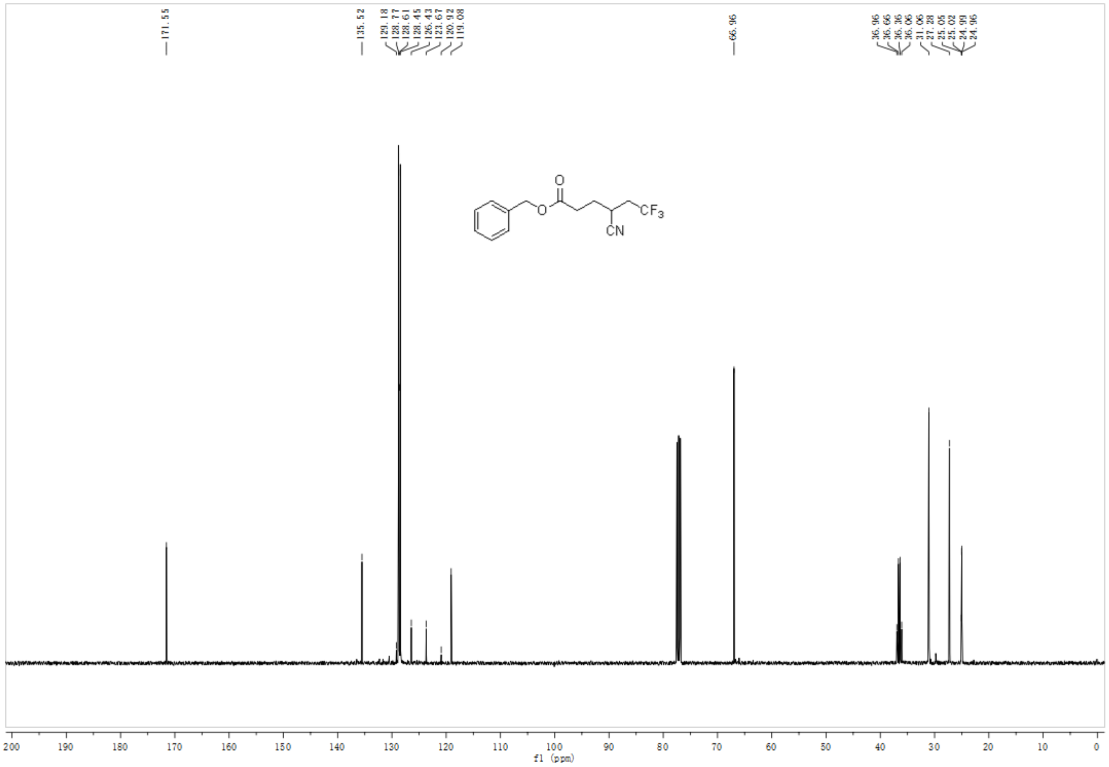

Figure S36 2m-^{13}C NMR

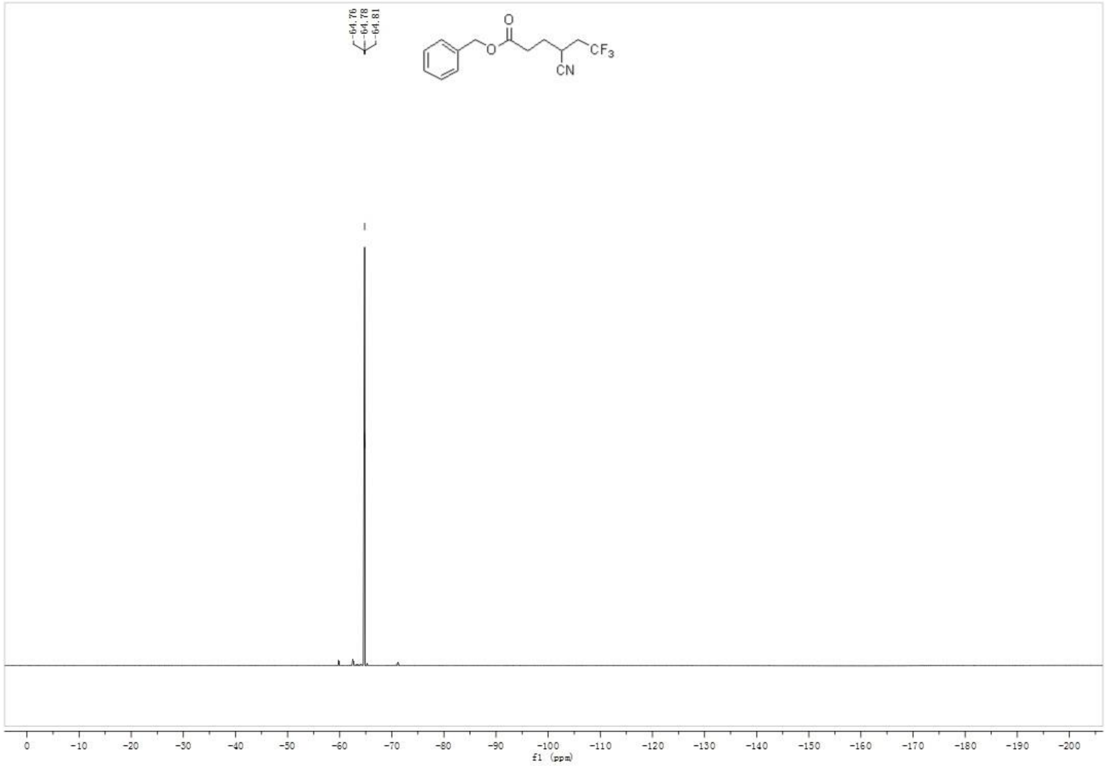

Figure S37 2m-^{19}F NMR

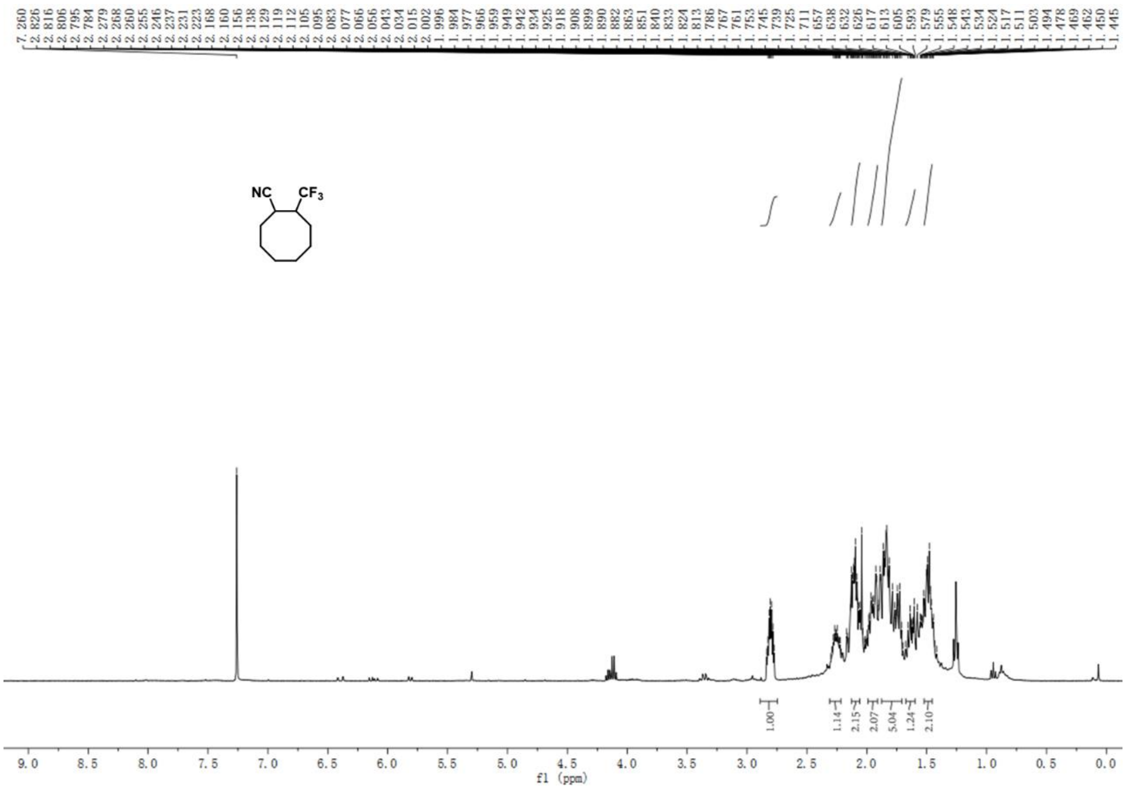

Figure S38 2n-^{1}H NMR

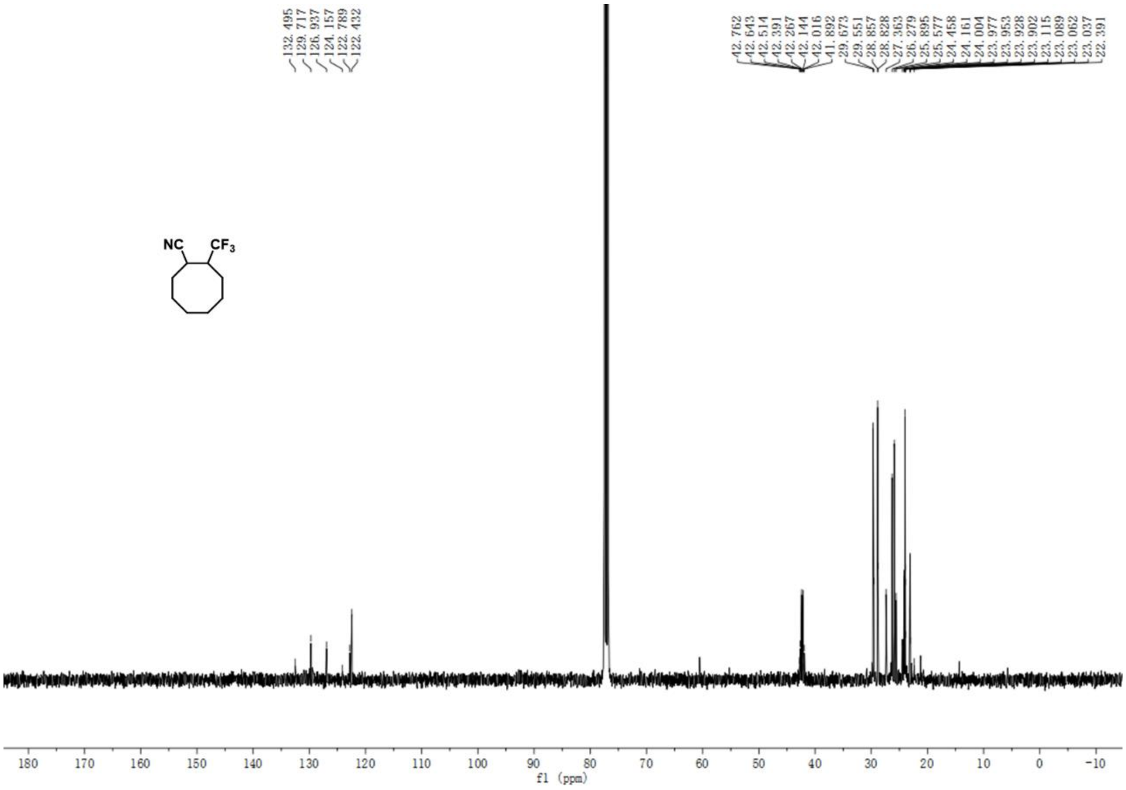

Figure S39 2n-^{13}C NMR

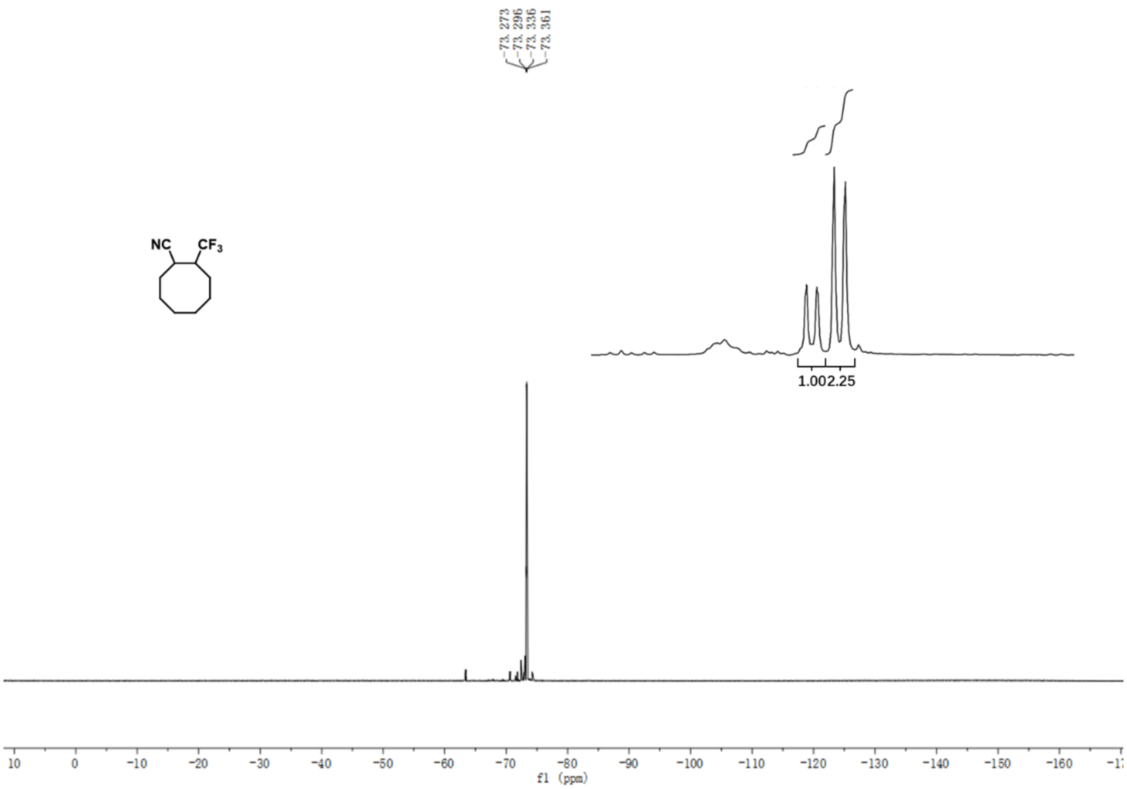

Figure S40 2n-^{19}F NMR

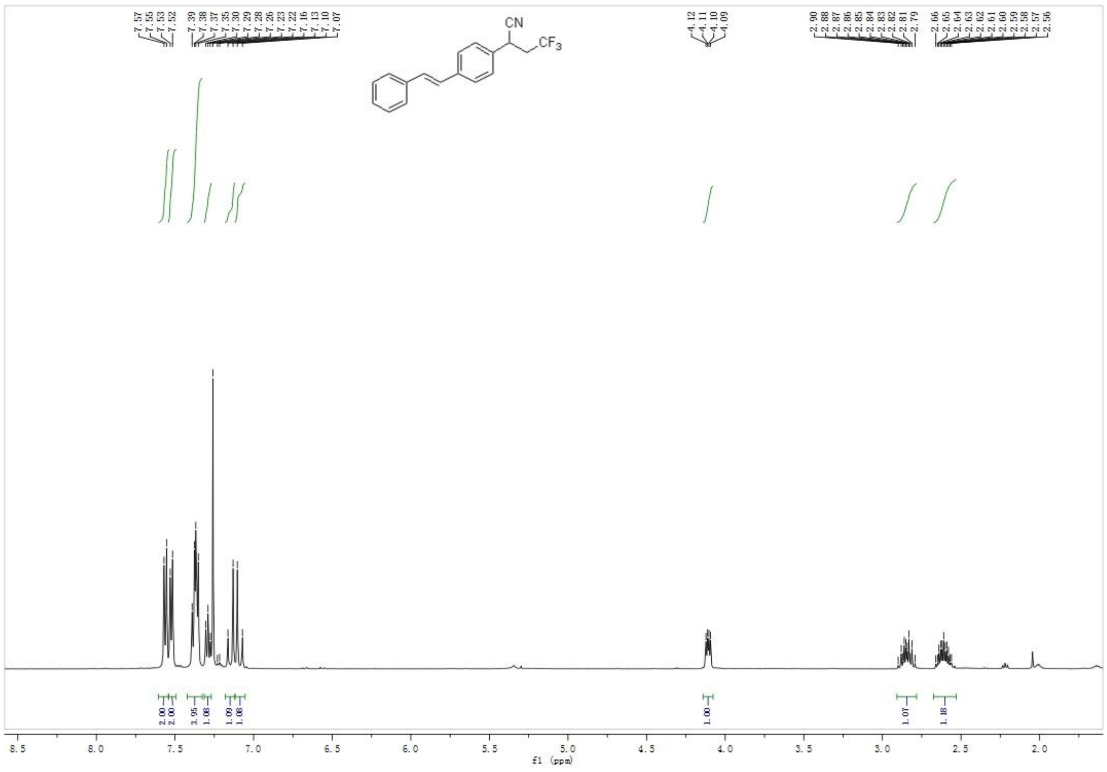

Figure S41 2p-^{1}H NMR

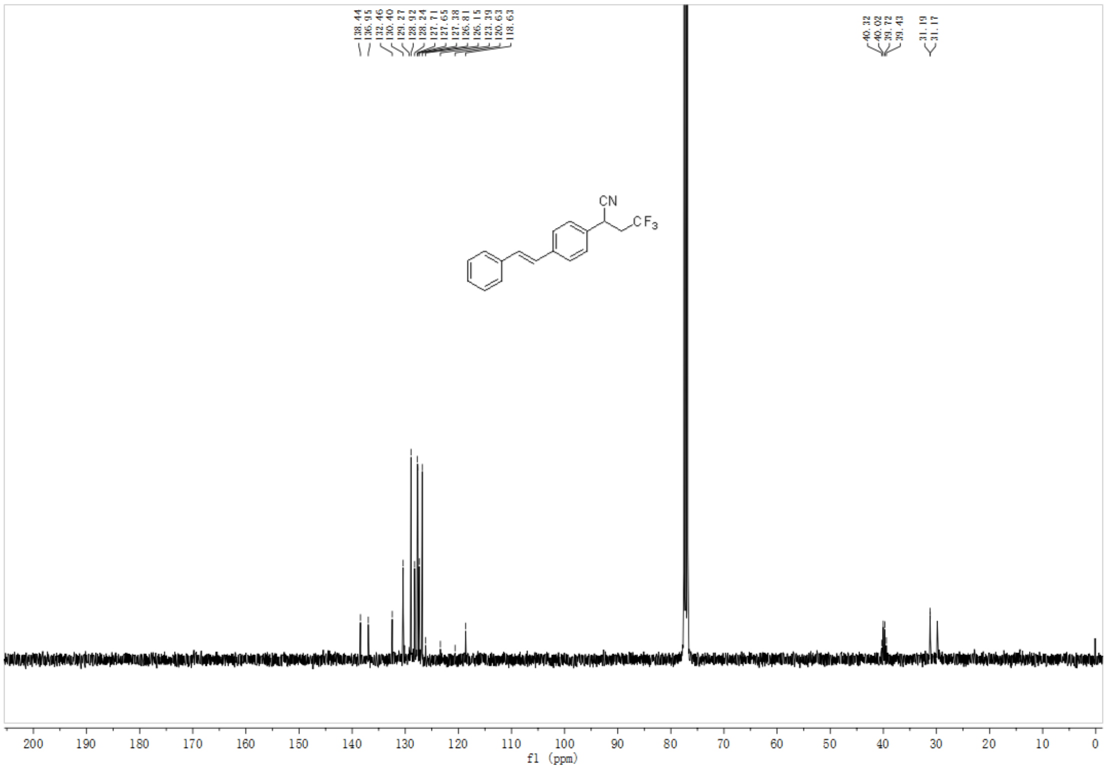

Figure S42 2p-^{13}C NMR

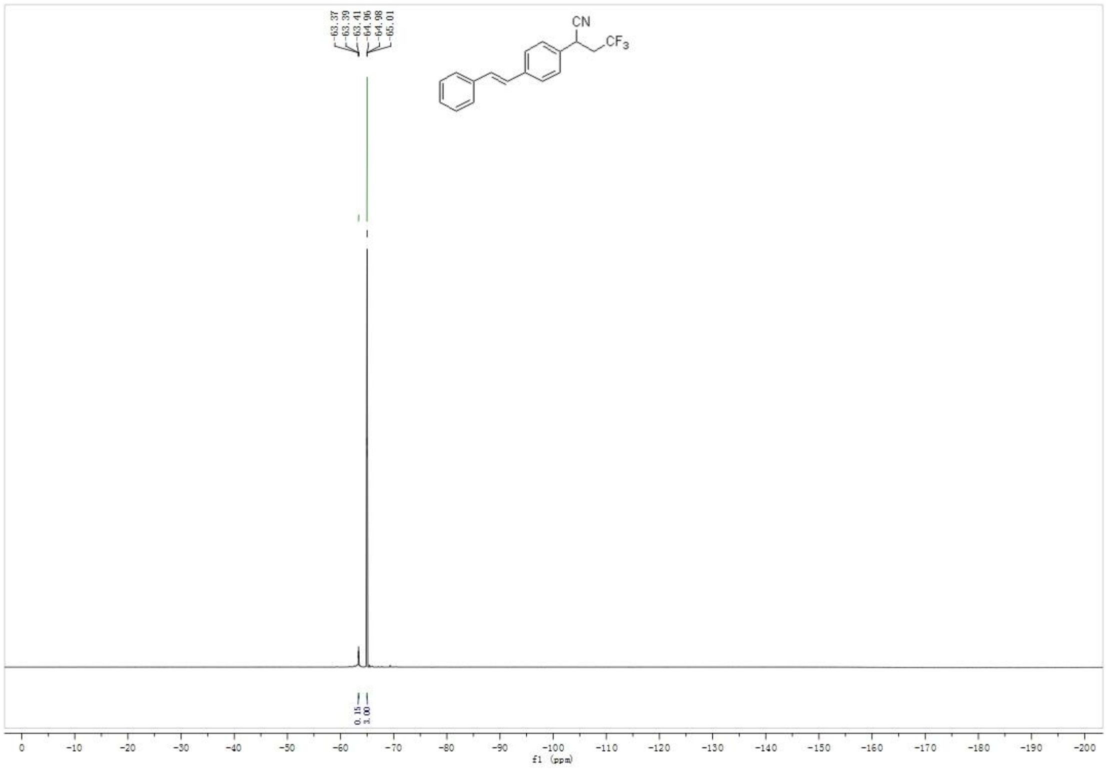

Figure S43 2p-^{19}F NMR

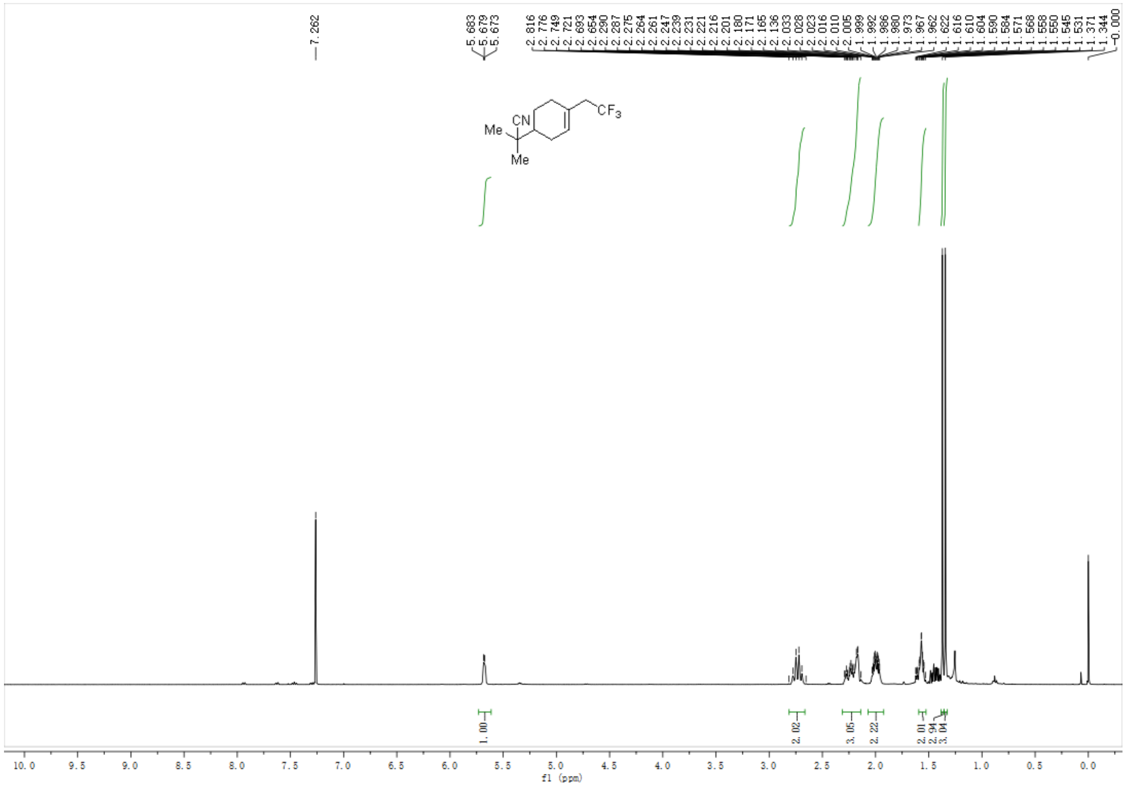

Figure S44 2q-^{1}H NMR

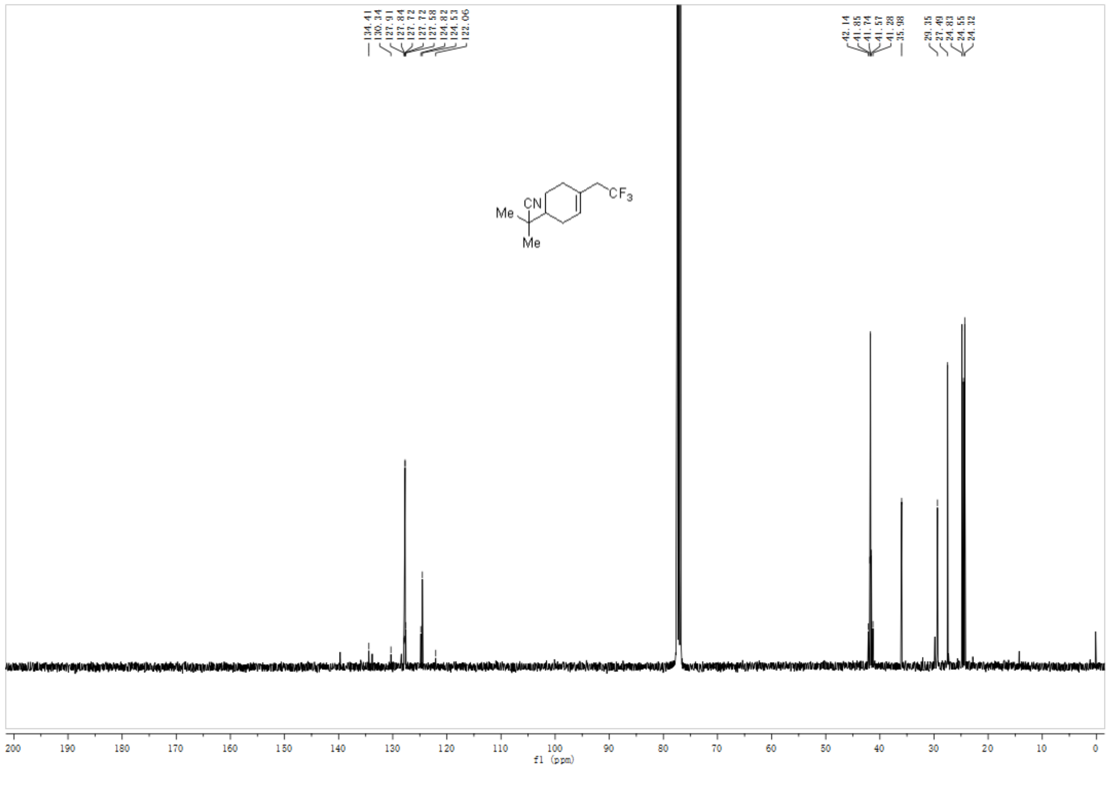

Figure S45 2q-^{13}C NMR

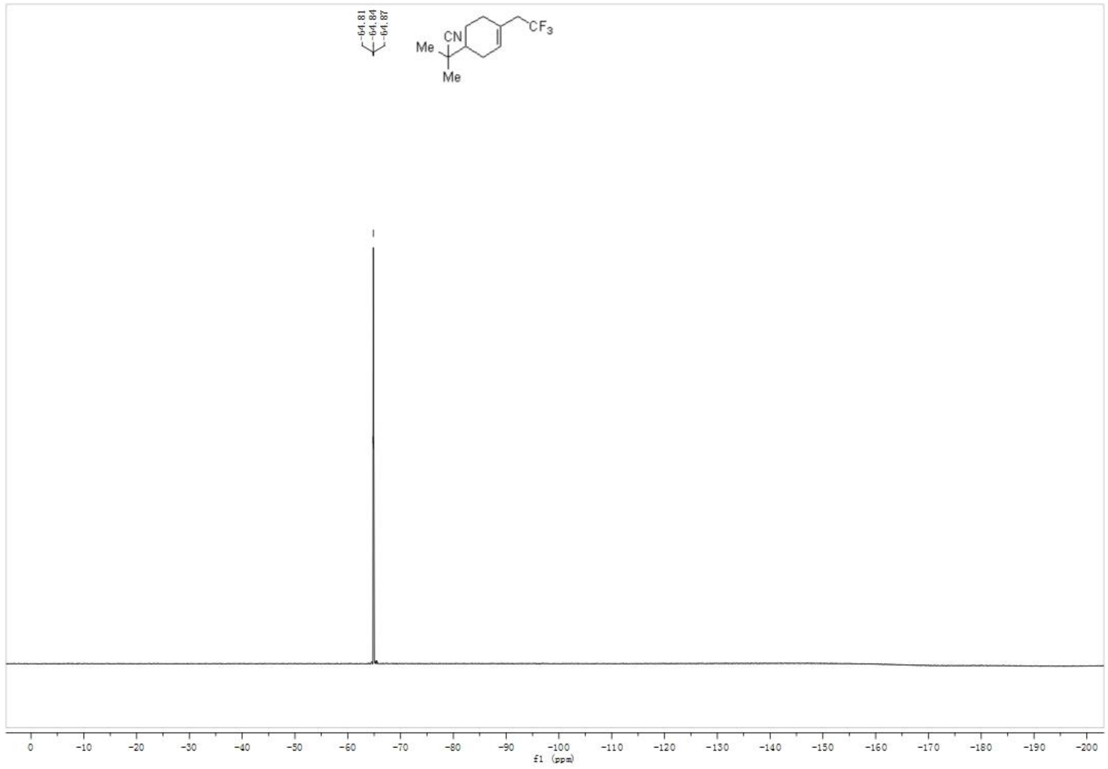

Figure S46 2q-^{19}F NMR

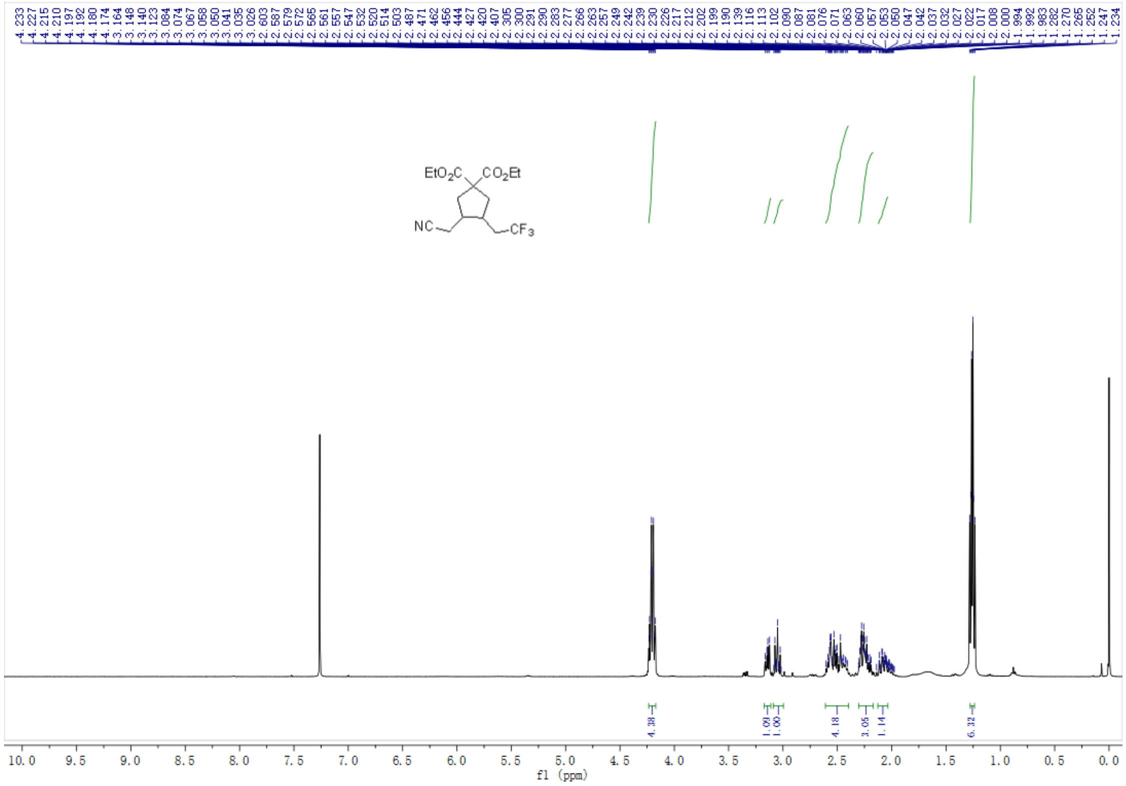

Figure S47 2r-^{1}H NMR

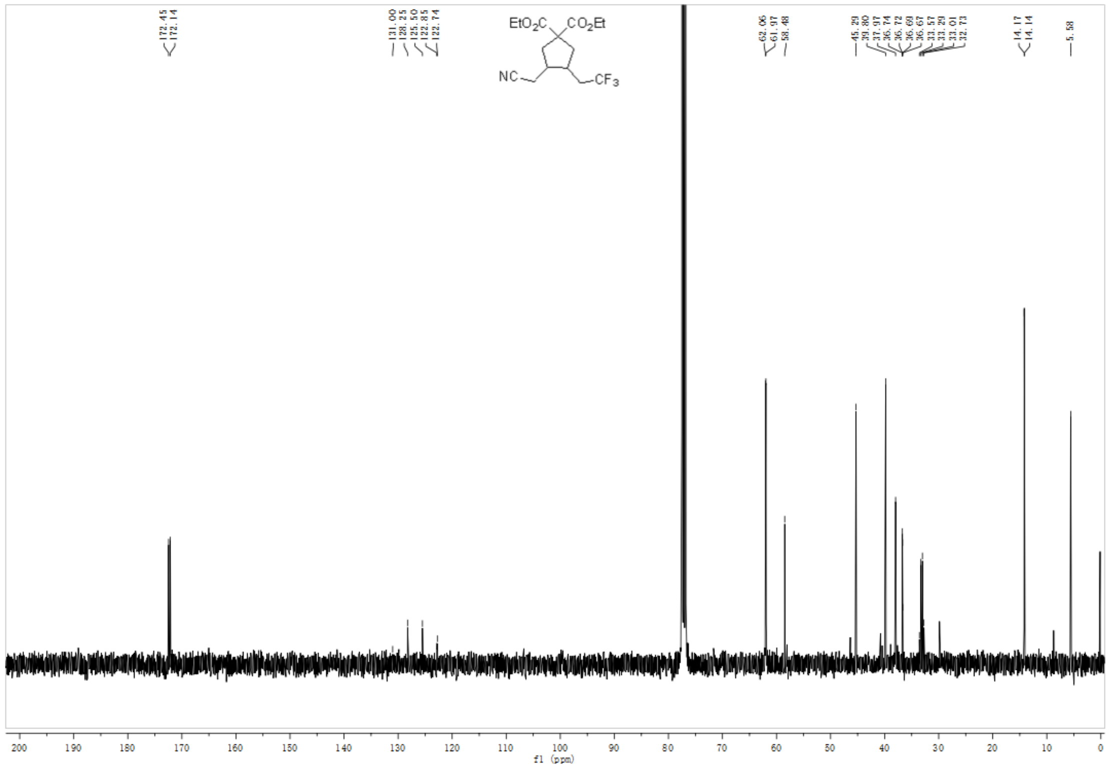

Figure S48 2r-^{13}C NMR

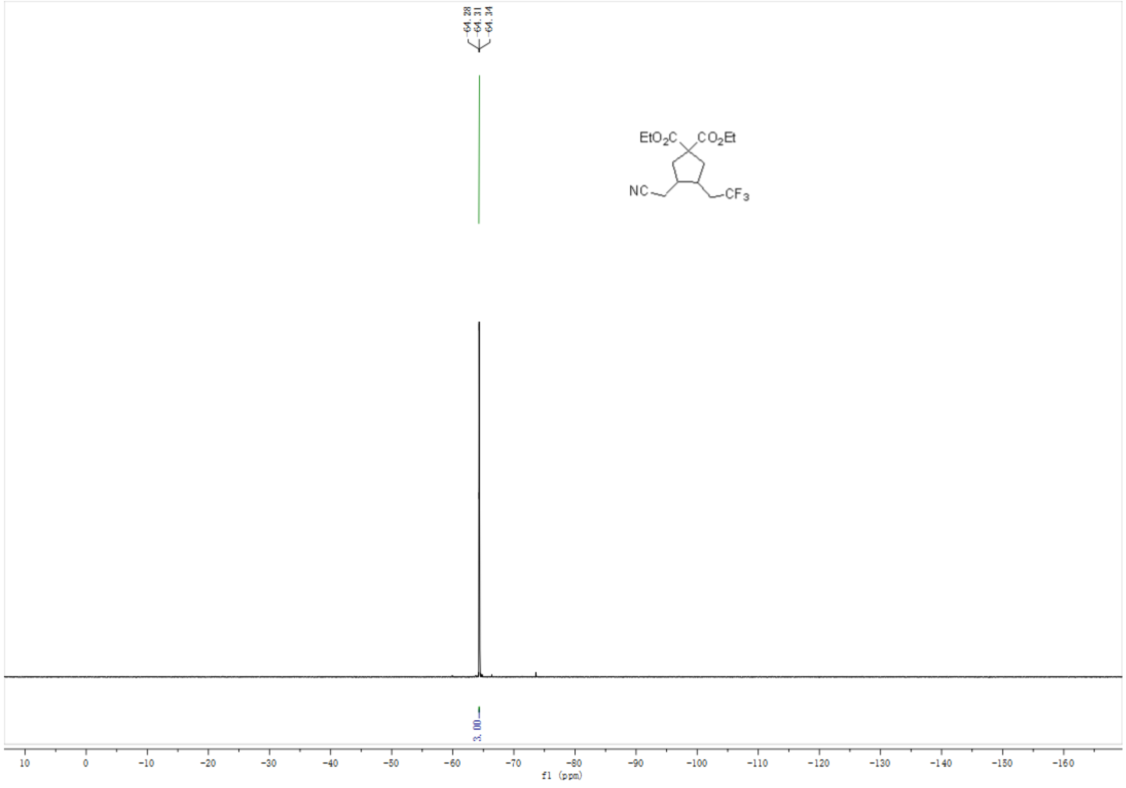

Figure S49 2r-^{19}F NMR

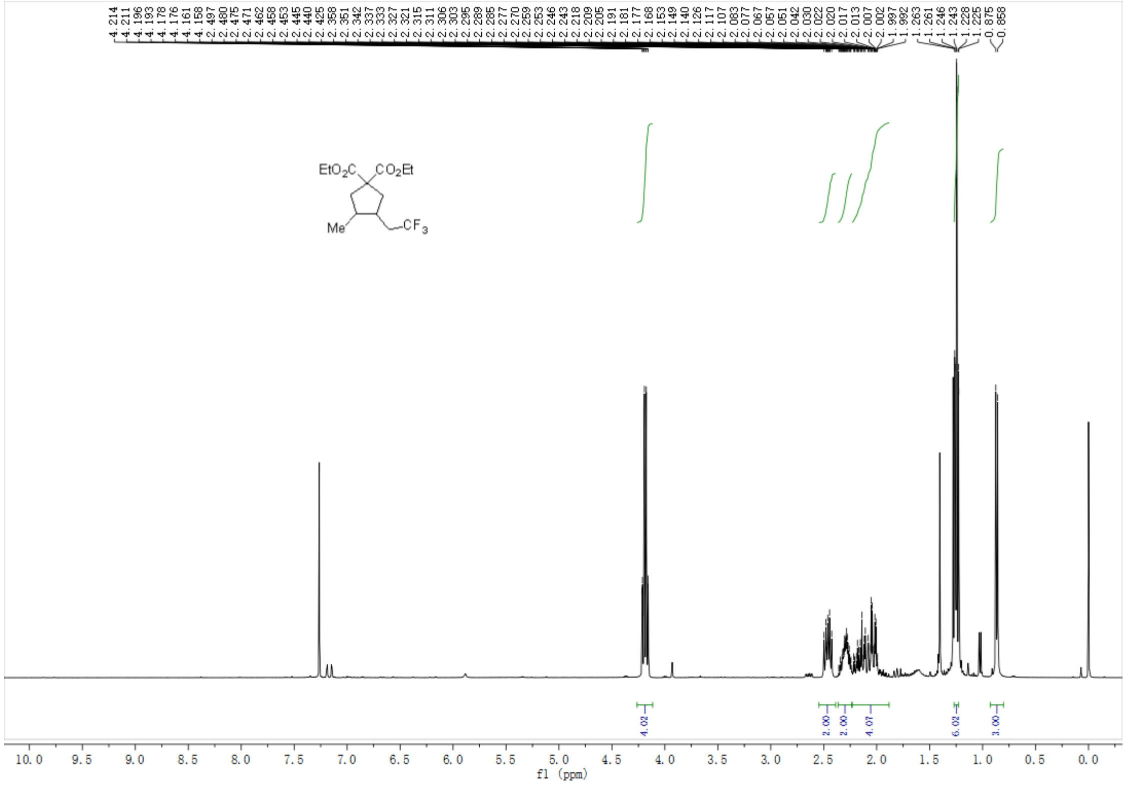

Figure S50 2r’-^{1}H NMR

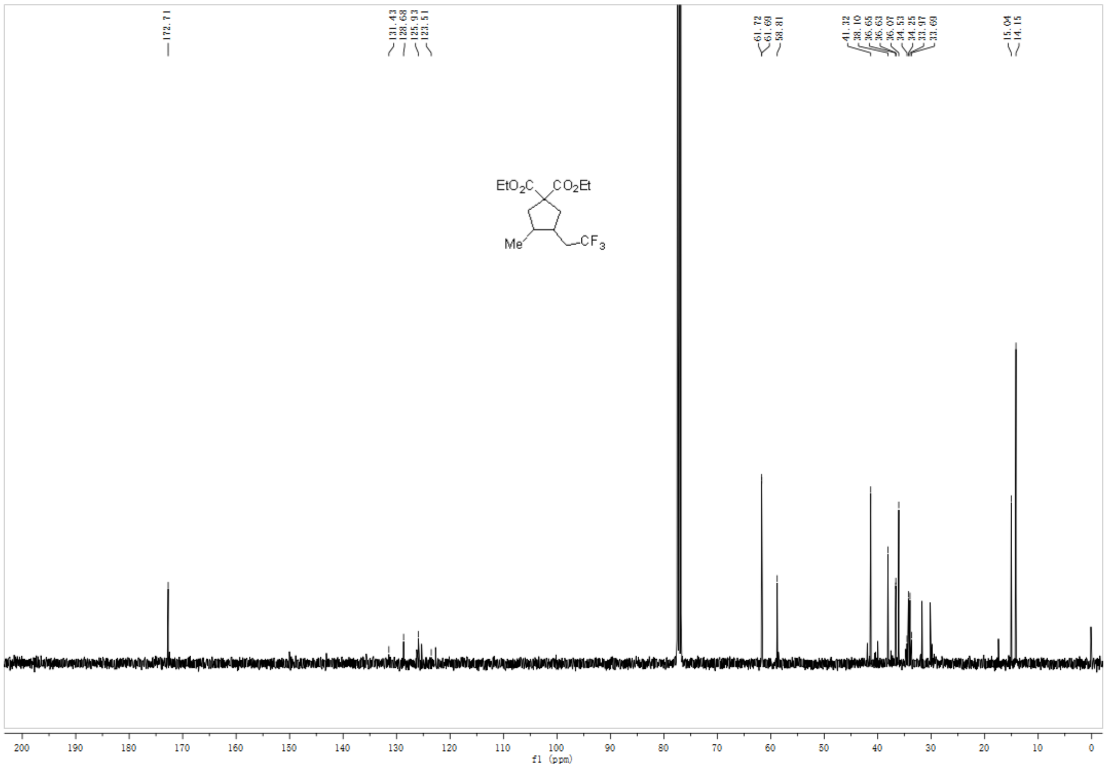

Figure S51 2r’-^{13}C NMR

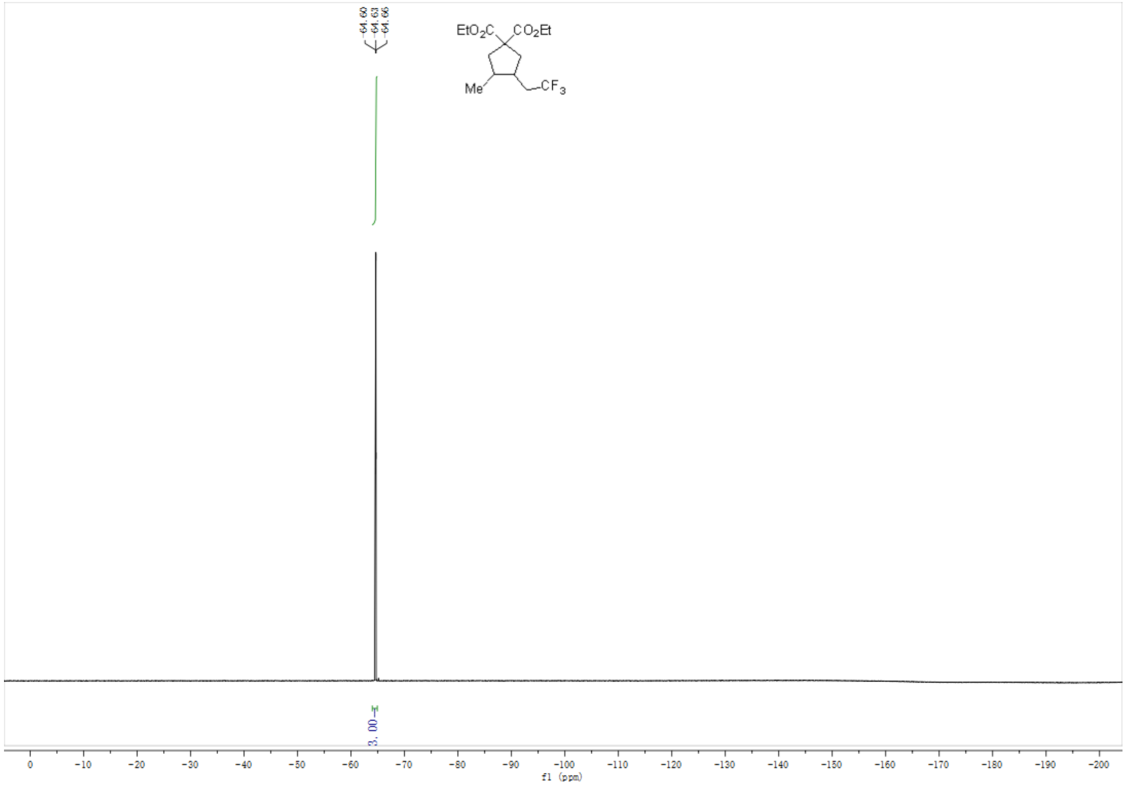

Figure S52 2r'-^{19}F NMR

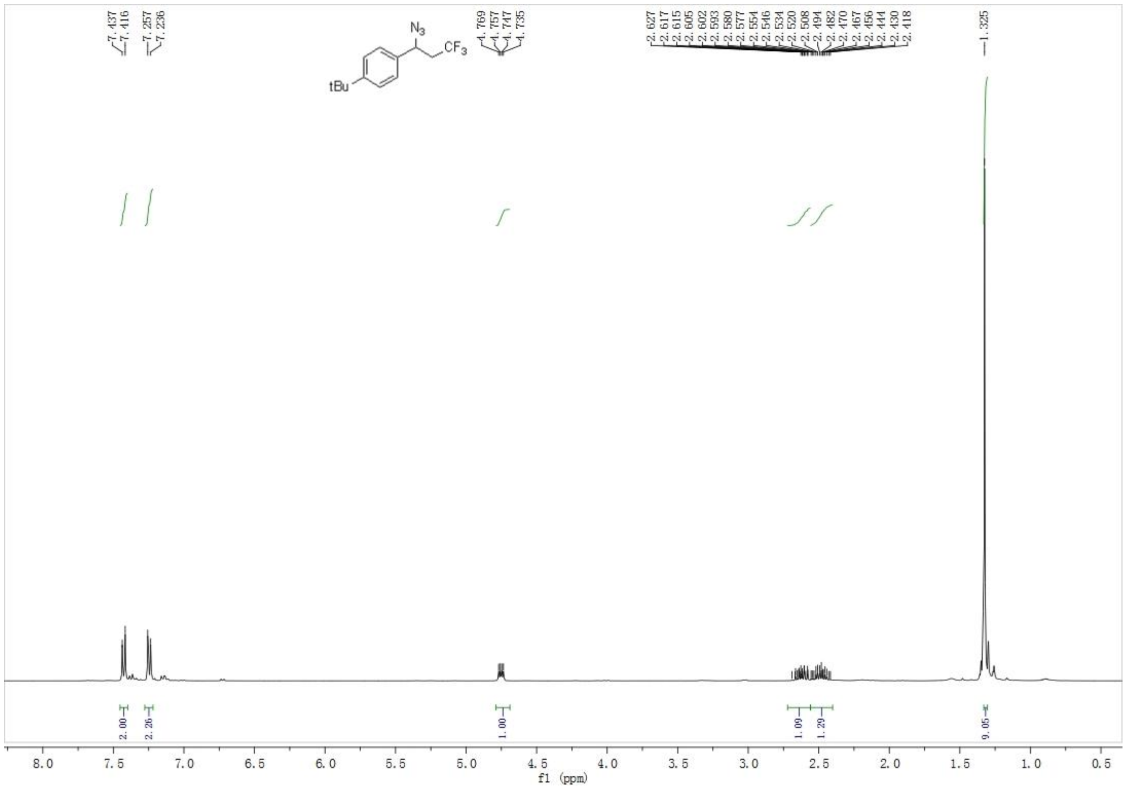

Figure S53 3a-^{1}H NMR

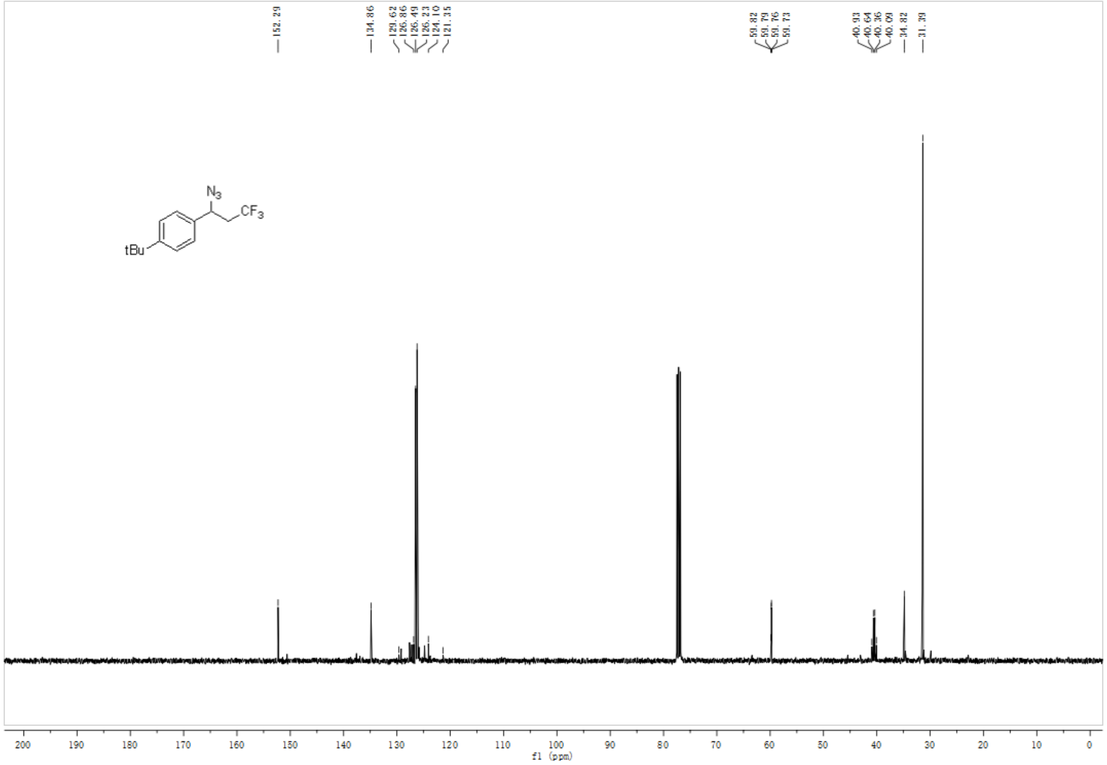

Figure S54 3a-^{13}C NMR

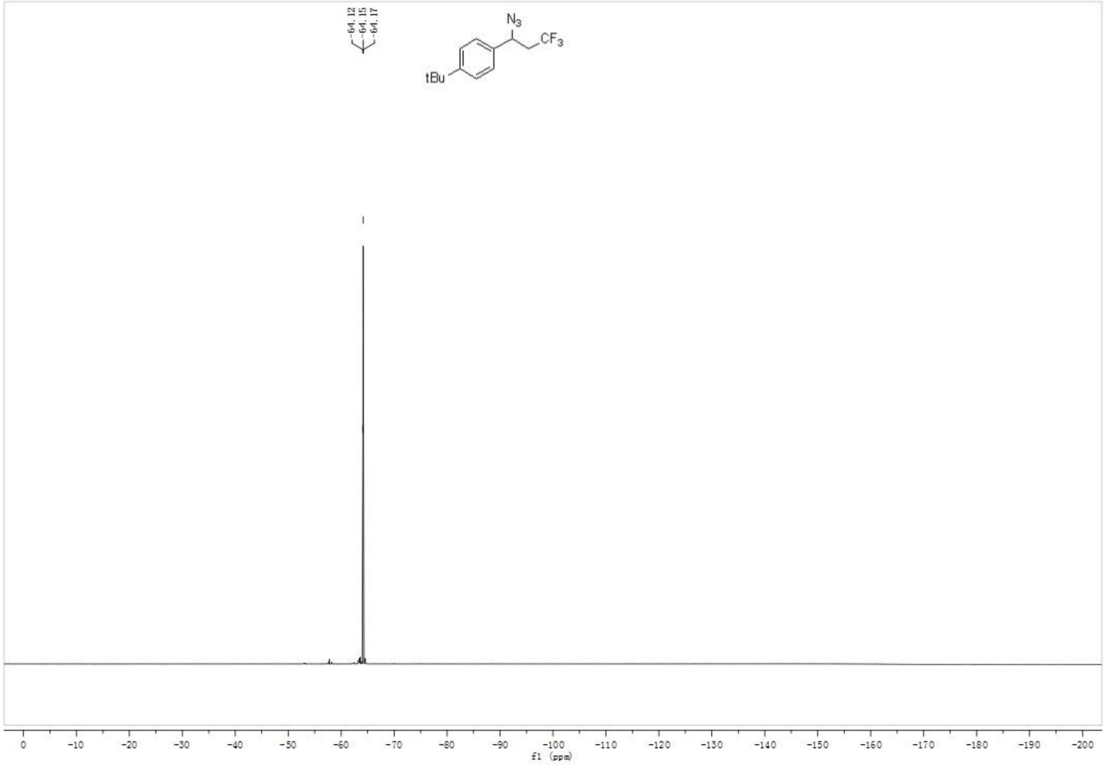

Figure S55 3a-^{19}F NMR

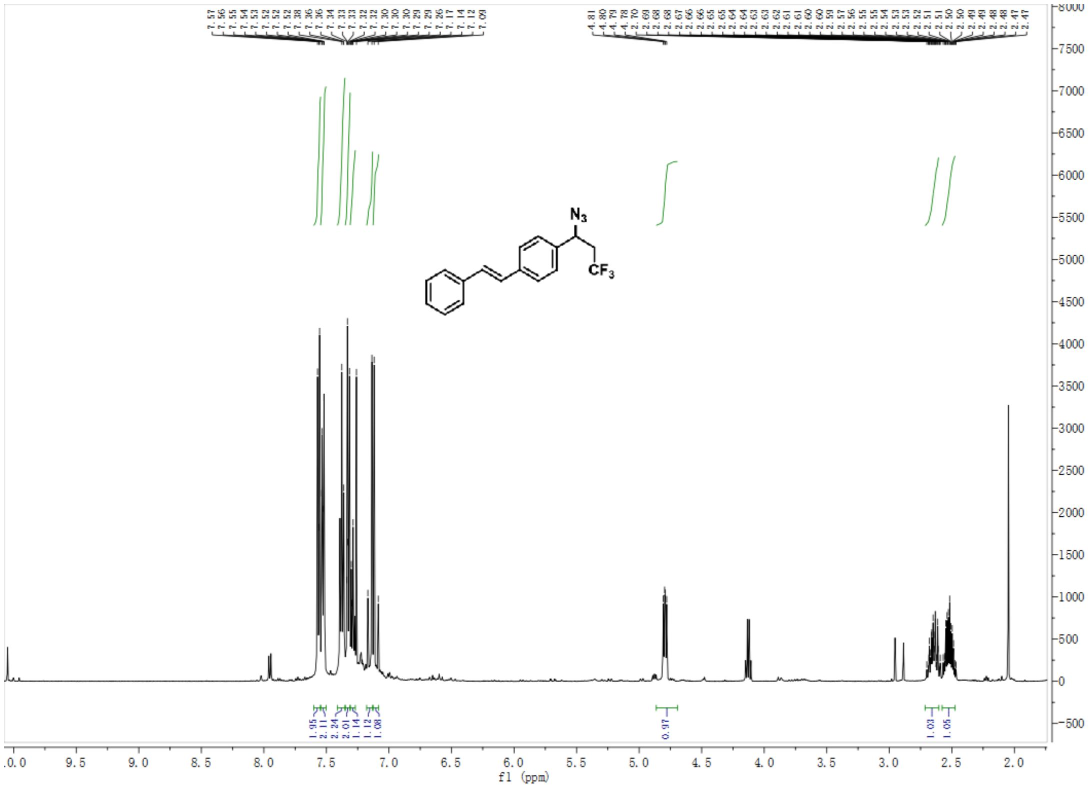

Figure S56 3b-^{1}H NMR

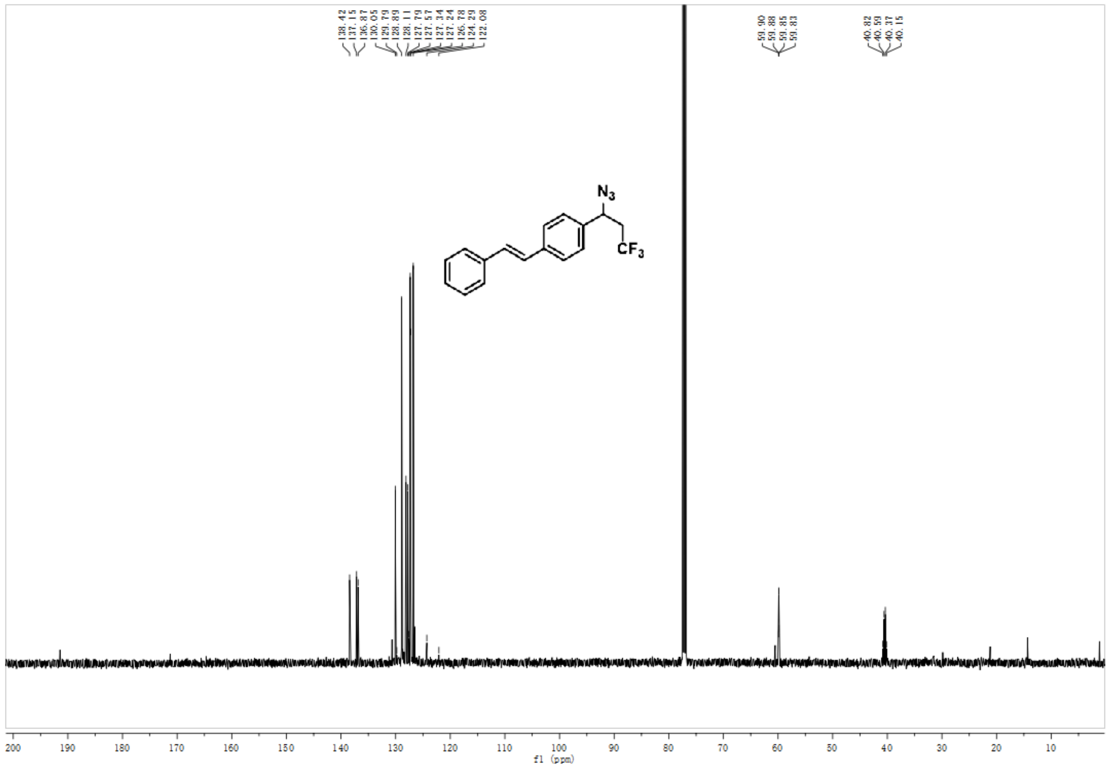

Figure S57 3b-^{13}C NMR

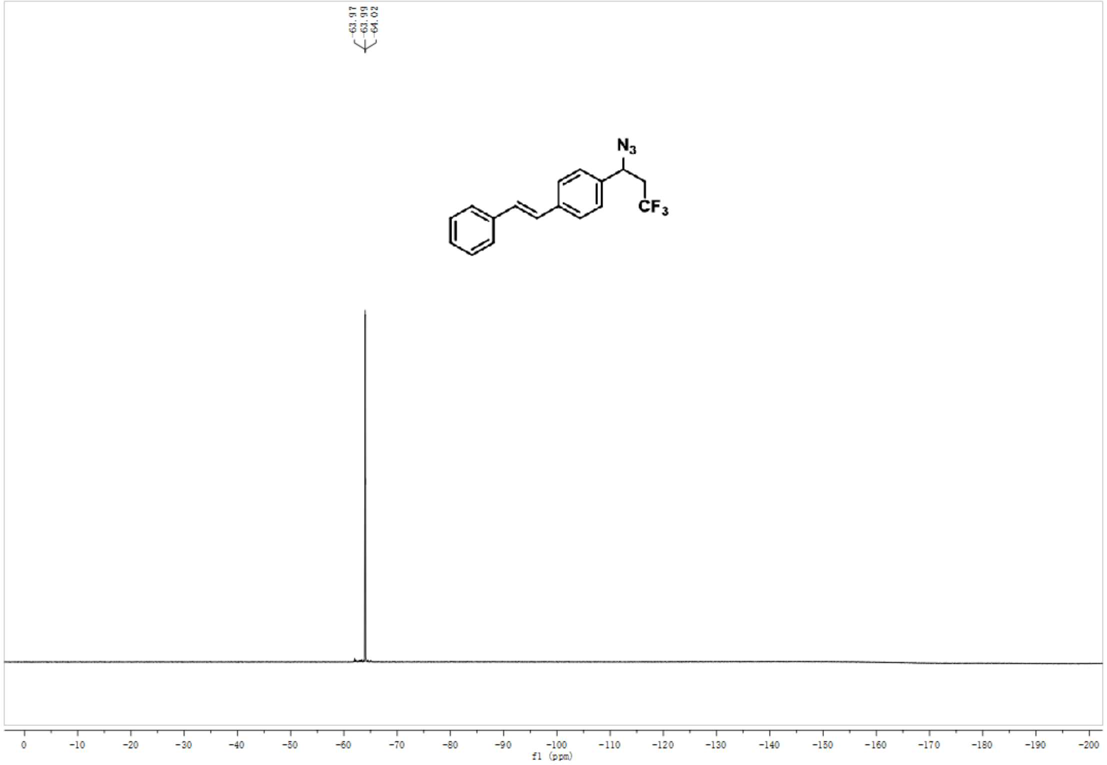

Figure S58 3b-^{19}F NMR

References.

(1) Diring, S.; Furukawa, S.; Takashima, Y.; Tsuruoka, T.; Kitagawa, S., Controlled Multiscale Synthesis of Porous Coordination Polymer in Nano/Micro Regimes. *Chem. Mater.* **2010,** *22* (16), 4531-4538.

(2) Carson, C. G.; Hardcastle, K.; Schwartz, J.; Liu, X.; Hoffmann, C., et al., Synthesis and Structure Characterization of Copper Terephthalate Metal–Organic Frameworks. *Eur. J. Inorg. Chem* **2009,** *2009* (16), 2338-2343.

(3) Wang, F.; Wang, D.; Wan, X.; Wu, L.; Chen, P., et al., Enantioselective Copper-Catalyzed Intermolecular Cyanotrifluoromethylation of Alkenes via Radical Process. *J. Am. Chem. Soc.* **2016,** *138* (48), 15547-15550.

(4) Guo, Q.; Wang, M.; Peng, Q.; Huo, Y.; Liu, Q., et al., Dual-Functional Chiral Cu-Catalyst-Induced Photoredox Asymmetric Cyanofluoroalkylation of Alkenes. *ACS Catal.* **2019,** *9* (5), 4470-4476.

(5) Zhang, M.; Lin, J.-H.; Xiao, J.-C., A Readily Available Trifluoromethylation Reagent and Its Difunctionalization of Alkenes. *Org. Lett.* **2021,** *23* (15), 6079-6083.

(6) Qi, B.; Zhang, T.; Li, M.; He, C.; Duan, C., Highly shape- and regio-selective peroxy–trifluoromethylation of styrene by metal–organic framework Cu3(BTC)2. *Catal. Sci. Technol.* **2017,** *7* (24), 5872-5881.

(7) Zhou, S.; Zhang, G.; Fu, L.; Chen, P.; Li, Y., et al., Copper-Catalyzed Asymmetric Cyanation of Alkenes via Carbonyl-Assisted Coupling of Alkyl-Substituted Carbon-Centered Radicals. *Org. Lett.* **2020,** *22* (16), 6299-6303.

(8) Mizuta, S.; Verhoog, S.; Engle, K. M.; Khotavivattana, T.; O'Duill, M., et al., Catalytic Hydrotrifluoromethylation of Unactivated Alkenes. *J. Am. Chem. Soc.* **2013,** *135* (7), 2505-2508.
